# Point estimation following two‐stage adaptive threshold enrichment clinical trials

**DOI:** 10.1002/sim.7831

**Published:** 2018-05-31

**Authors:** Peter K. Kimani, Susan Todd, Lindsay A. Renfro, Nigel Stallard

**Affiliations:** ^1^ Warwick Medical School University of Warwick Coventry CV4 7AL UK; ^2^ Department of Mathematics and Statistics University of Reading Reading RG6 6AX UK; ^3^ Division of Biomedical Statistics and Informatics Mayo Clinic Rochester MN 55905 USA

**Keywords:** biomarker, multistage, personalized medicine, subgroup or subpopulation selection, targeted therapy

## Abstract

Recently, several study designs incorporating treatment effect assessment in biomarker‐based subpopulations have been proposed. Most statistical methodologies for such designs focus on the control of type I error rate and power. In this paper, we have developed point estimators for clinical trials that use the two‐stage adaptive enrichment threshold design. The design consists of two stages, where in stage 1, patients are recruited in the full population. Stage 1 outcome data are then used to perform interim analysis to decide whether the trial continues to stage 2 with the full population or a subpopulation. The subpopulation is defined based on one of the candidate threshold values of a numerical predictive biomarker. To estimate treatment effect in the selected subpopulation, we have derived unbiased estimators, shrinkage estimators, and estimators that estimate bias and subtract it from the naive estimate. We have recommended one of the unbiased estimators. However, since none of the estimators dominated in all simulation scenarios based on both bias and mean squared error, an alternative strategy would be to use a hybrid estimator where the estimator used depends on the subpopulation selected. This would require a simulation study of plausible scenarios before the trial.

## 1 INTRODUCTION

An area of recent interest in development of new therapies is stratified medicine, which involves using a biomarker to stratify patients into subgroups to distinguish those with the best likelihood of responding to particular treatments. If a biomarker has two levels, it is common to refer to one level as biomarker negative and the other as biomarker positive. We consider predictive biomarkers that allow the possibility of differences in treatment effects in different subpopulations, that is, a treatment by biomarker interaction effect.^1^

Advances in genetics have played a key role in stratified medicine, where biomarkers are based on genes. This has led to targeted therapies, where investigators determine a target subset of patients (subpopulation) and develop a drug (a targeted therapy) expected to be more efficacious than the control for these patients, and is possibly not beneficial to others. The target subpopulation may consist of patients with a certain gene (specifically a gene containing a certain allele) or platform of genes (specifically certain alleles corresponding to multiple genes). However, genes are not the only characteristics that are used to define a subset of patients. Examples of other biomarkers in the cancer setting include the size of tumor, protein level in the blood, and graded scores. When the clinical utility of the biomarker is not very strong or clear from previous studies, the biomarker stratified design may be used to test the effect of an experimental treatment. In this design, a trial enrolls patients from the full population but with provision for analyses of outcomes from the subpopulation.

One methodological challenge in stratified medicine is how to design and analyze efficient clinical trials that incorporate identification of the subpopulation that will benefit from the experimental treatment. An efficient design in late phase clinical trials is the two‐stage adaptive enrichment design.^2^ In stage 1, patients are recruited from the full population and data are used to perform an interim analysis to decide whether, in stage 2, enrollment will be from the full population or the subpopulation. The final confirmatory analysis uses data from both stages. Although the design is efficient because stage 1 data are used for subpopulation selection and confirmatory analysis, the latter is complex because of inclusion of subpopulation selection data.

We consider the case of a continuous (or a graded score) biomarker where the cut‐off value to distinguish between biomarker positive and negative patients is not definite from previous trials. Consequently, several candidate cut‐off values are possible, with trial data used to determine the cut‐off value. Simon and Simon^2^ refer to such a design that includes threshold determination as an adaptive threshold enrichment design. We give examples of clinical trials where this design can be used in Section 2.1.

Subpopulation selection based on the treatment effect can be advantageous because using an appropriate rule, the subgroup is selected in the case where there is apparent benefit in the subgroup and not in its complement (qualitative interaction) such as was observed by Mok et al.^3^ The full population is selected if there is apparent benefit in the full population including when the drug benefits the subgroup and its complement with different magnitudes (quantitative interaction) such as was observed in Tran et al.^4^ A subpopulation selection based on a hypothesis test for interaction only would not be able to distinguish between the two types of interactions.

Previous research that considers analysis of adaptive threshold enrichment trials focuses on control of type I error rate and power with less emphases on point estimation.^2^, ^5^ Recently, Li et al^6^ have derived expressions for the biases of estimators that ignore the adaptation but do not propose point estimators that account for subpopulation selection. Kimani et al^7^ and Kunzmann et al^8^ have developed estimators for a setting analogous to a single fixed cut‐off value. However, these estimators do not allow for using stage 1 data to determine the cut‐off value in an adaptive threshold enrichment trial.

A setting similar to an adaptive threshold enrichment design is that of treatment selection, where a control is compared to multiple experimental treatments, with stage 1 data used to select the experimental treatment to test further in stage 2.^9^, ^10^, ^11^, ^12^, ^13^, ^14^, ^15^, ^16^ Although several point estimators for this setting exist, they cannot be applied directly in adaptive threshold enrichment clinical trials because the correlation structure of the stage 1 sample means used for selection is different.

In this paper, we develop estimators that account for subpopulation selection following adaptive threshold enrichment trials using the principles that have been used to obtain point estimators that account for treatment selection. Two unbiased estimators build on the works by Kimani et al^7^ and Robertson et al.^17^ Two estimators build on the works by Whitehead^18^ and Stallard and Todd^10^ and involve deriving the bias function to calculate bias and subtracting bias from the naive estimator. The last is a shrinkage estimator and builds on the works by Hwang^19^ and Carreras and Brannath.^14^

## 2 DESCRIPTION OF THE SETTING AND NAIVE ESTIMATION

### 2.1 Motivation and notation

A condition where continuous biomarkers are tested and so the adaptive threshold design may be used is depression. Examples of continuous predictive biomarkers in depression are protein levels in the blood and an electrophysiological measure.^20^ While introducing notation, we describe features of clinical trials that are key in our methodology based on the setting of depression.

Patients' outcomes will be assumed to be normally distributed with a known standard deviation σ. In the context of depression, Uher et al^20^ perform simulations to give a guidance of the treatment effect size to be sought when predictive biomarkers are evaluated. One outcome measure they consider that is widely used in trials is the Hamilton Rating Scale for Depression (HRSD) score and is usually assumed to be normally distributed. For a trial of a prespecified duration of treatment, the aim may be to estimate the mean difference (experimental arm minus control arm) in HRSD scores between two interventions at the final follow‐up visit. Based on two trials,^21^, ^22^ the standard deviation of HRSD scores may be taken to be 7, that is, σ = 7.

We will consider trials that allow stopping for futility at an interim analysis if the observed treatment difference is less than some value b that we refer to as the futility boundary. The UK NICE guidelines recommend that an intervention for depression should demonstrate a difference of at least 3 HRSD points^20^ to be considered superior to its comparator. Therefore, at an interim analysis, the treatment may be deemed not to warrant further testing if the observed mean difference <2 (slightly less than the recommended value of 3), that is, b = 2.

We assume that a single continuous biomarker is used to identify the patients who benefit from a new intervention. We assume that in regard to biomarker values, there is monotonicity in treatment effect so that a higher biomarker value leads to a bigger treatment effect or a higher biomarker value leads to a smaller treatment effect. For ease of notation, we use the latter to develop methodology. Note that, if a higher biomarker value leads to a bigger treatment effect, the biomarker values can be transformed by multiplying by −1.

Using some biomarker threshold values, the full population (F) is partitioned into distinct partitions. For example, if F is subdivided into four partitions, the candidate threshold values c_1_, c_2_, c_3_, and c_4_ are such that patients in partitions 1, 2, 3, and 4 have biomarker values less than c_1_, between c_1_ and c_2_, between c_2_ and c_3_, and between c_3_ and c_4_, respectively. The true mean differences in partitions 1 to 4 are denoted by δ_1_, δ_2_, δ_3_, and δ_4_, respectively. We denote the number of partitions by K so that, in this case, K = 4. We refer to the parts of F below threshold values c_1_, c_2_, c_3_, and c_4_ as subpopulations S_1_, S_2_, S_3_, and S_4_. Note for K = 4, S_K_ = S_4_ = F, and S_1_, S_2_, S_3_, and S_4_ consist of partition 1, partitions 1 and 2, partitions 1 to 3, and partitions 1 to 4, respectively. The true mean differences in S_1_, S_2_, S_3_, and S_4_ are denoted by θ_1_, θ_2_, θ_3_, and θ_4_, respectively. If, as expected, a higher biomarker value leads to a smaller treatment effect, then δ_1_ ≥ δ_2_ ≥ δ_3_ ≥ δ_4_ and θ_1_ ≥ θ_2_ ≥ θ_3_ ≥ θ_4_.

**Figure 1:**
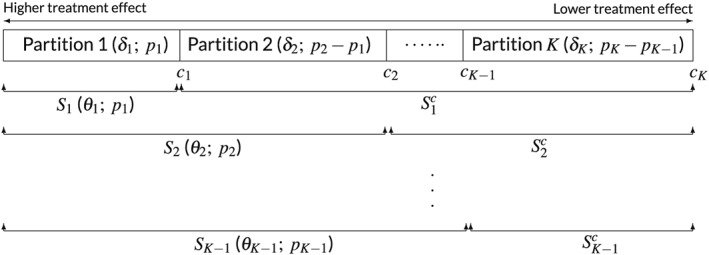
Partitioning of the full population. Partitions to the left are expected to have bigger treatment effects. The pairs in the brackets are true mean differences and prevalences for partitions and candidate subpopulations

We assume that the threshold values c_1_,…,c_K_ are prespecified. There are different ways for the choice of the thresholds values. For K = 4, quartiles may be used so that the prevalences for S_1_ to S_4_ are p_1_ = 0.25, p_2_ = 0.50, p_3_ = 0.75, and p_4_ = 1, respectively. Consequently, the partitions have equal prevalence (0.25) since if we set p_0_ = 0, p_i_ − p_i − 1_ = 0.25(i = 1,…,4). In some instances, the threshold values are chosen based on aspects such as biological activity so that the prevalences for partitions are not equal. Figure 1 summarizes the partitioning of F for any K ≥ 3.

### 2.2 Hypothetical two‐stage adaptive threshold enrichment clinical trial

Predictive assessment of continuous biomarkers can been done in single‐stage clinical trials.^23^, ^24^ The alternative is to use the two‐stage adaptive threshold enrichment design, which is more efficient as more resources can be focused on the subpopulation that is most likely to benefit from the new treatment.^24^ The design has been used in recent trials with time‐to‐event (progression‐free survival) outcome data.^5^, ^25^, ^26^ As we propose in this paper, the design can be similarly used in trials with normally distributed outcome data. We note in Section 6 that the methods developed in this paper can be adapted for time‐to‐event outcome data.

We describe the form of the adaptive threshold enrichment design that we consider based on a hypothetical trial for depression, where for example protein level is used to partition *F* into quartiles. In stage 1, the trial recruits *n*_11_ = 90, *n*_12_ = 90, *n*_13_ = 90, and *n*_1*K*_ = *n*_14_ = 90 patients in partitions 1 to 4. The number of patients in *S*_1_ to *S*_4_ are *m*_11_ = 90, *m*_12_ = 180, *m*_13_ = 270, and *m*_1*K*_ = *n*_14_ = 360, respectively, since $m_{1i}=\sum_{i^{\prime }=1}^{i}n_{1i^{\prime }}$ (*i* = 1,…,4). For simplicity, we assume that, in each partition, the 90 patients are equally split between the control and the experimental treatment. The outcome of interest is HRSD score and is assumed to be normally distributed with *σ* = 7. Let $\tau_{11}^{2}=4\sigma^{2}/n_{11}$, $\tau_{12}^{2}=4\sigma^{2}/n_{12}$, $\tau_{13}^{2}=4\sigma^{2}/n_{13}$, and $\tau_{14}^{2}=4\sigma^{2}/n_{14}$, the stage 1 sample mean differences in partitions 1 to 4 are ${\bar{X}}_{11}∼N(\delta_{1},\tau_{11}^{2})$, ${\bar{X}}_{12}∼N(\delta_{2},\tau_{12}^{2})$, ${\bar{X}}_{13}∼N(\delta_{2},\tau_{13}^{2})$, and ${\bar{X}}_{14}∼N(\delta_{4},\tau_{14}^{2})$, respectively. Let $\sigma_{11}^{2}=4\sigma^{2}/m_{11}$, $\sigma_{12}^{2}=4\sigma^{2}/m_{12}$, $\sigma_{13}^{2}=4\sigma^{2}/m_{13}$, and $\sigma_{14}^{2}=4\sigma^{2}/m_{14}$, the stage 1 sample means in *S*_1_ to *S*_4_ are ${\bar{Y}}_{11}∼N(\theta_{1},\sigma_{11}^{2})$, ${\bar{Y}}_{12}∼N(\theta_{2},\sigma_{12}^{2})$, ${\bar{Y}}_{13}∼N(\theta_{3},\sigma_{13}^{2})$, and ${\bar{Y}}_{14}∼N(\theta_{4},\sigma_{14}^{2})$, respectively. If the number of patients in a partition is not equally split between the control and the experimental treatment, the expressions for $\tau_{11}^{2}$ to $\tau_{14}^{2}$ and $\sigma_{11}^{2}$ to $\sigma_{14}^{2}$ are different. Note that, in this hypothetical trial, $\tau_{11}^{2}=\text{⋯}=\tau_{14}^{2}=\sigma_{11}^{2}=2.178$, $\sigma_{12}^{2}=1.089$, $\sigma_{13}^{2}=0.726$, and $\sigma_{14}^{2}=0.544$. The random vectors ${\bar{X}}_{1}={\left({\bar{X}}_{11},{\bar{X}}_{12},{\bar{X}}_{13},{\bar{X}}_{14}\right)}^{\prime }$ and ${\bar{Y}}_{1}={\left({\bar{Y}}_{11},{\bar{Y}}_{12},{\bar{Y}}_{13},{\bar{Y}}_{14}\right)}^{\prime }$ have a linear relationship and are multivariate normal with mean vectors ***δ***=(*δ*_1_,*δ*_2_,*δ*_3_,*δ*_4_)*′* and ***θ***=(*θ*_1_,*θ*_2_,*θ*_3_,*θ*_4_)*′*, respectively (see supplementary material). Hence, selection rules based on observed values for ${\bar{X}}_{1}$ can be restated using the observed values for ${\bar{Y}}_{1}$ and vice versa.

Since a higher biomarker value is expected to lead to lower treatment effect, the largest subpopulation for which the observed stage 1 sample mean difference (in HRSD scores) is ≥*b* is selected to continue to stage 2. If the observed stage 1 sample mean differences in *S*_1_, *S*_2_, *S*_3_, and *S*_4_ = *F* are all less than *b*, the trial stops for futility. Note that the selected subpopulation is a random variable determined by observed stage 1 data. We use lower case *s* $\left(s\in \{1,\text{⋯},4\}\right)$ as the index for the “observed” selected subpopulation, with *S*_*s*_ $\left(s\in \{1,\text{⋯},4\}\right)$ denoting the selected subpopulation. At the end of stage 2, the primary objective is to obtain an estimate for *θ*_*s*_, using an estimator that has good properties such as being mean unbiased and having small mean squared error (MSE).

Suppose that the stage 1 observed sample mean differences in partitions 1 to 4 are ${\bar{x}}_{11}=3$, ${\bar{x}}_{12}=2$, ${\bar{x}}_{13}=0.8$, and ${\bar{x}}_{14}=0$ so that *S*_1_ to *S*_4_ stage 1 observed sample mean differences are ${\bar{y}}_{11}=3$, ${\bar{y}}_{12}=2.5$, ${\bar{y}}_{13}=1.93$, and ${\bar{y}}_{14}=1.45$. Subpopulation 2 would be selected, that is, *S*_*s*_ = *S*_2_, since it is the largest subpopulation with observed mean difference of at least 2 points, so that *θ*_*s*_ = *θ*_2_.

In stage 2, the trial recruits *n*_21_ = 120 and *n*_22_ = 120 patients in partitions 1 and 2, respectively. The number of patients in *S*_1_ and *S*_2_ are *m*_21_ = 120 and *m*_22_ = 240, respectively, since $m_{2i}=\sum_{i^{\prime }=1}^{i}n_{2i^{\prime }}$ (*i* = 1,…,*s*). The sample sizes *n*_21_ and *n*_22_ and, hence, *m*_21_ and *m*_22_, should be prespecified in advance for example by fixing the total stage 2 sample size and the ratio of allocation to the selected partitions. Let $\tau_{21}^{2}=4\sigma^{2}/n_{21}$ and $\tau_{22}^{2}=4\sigma^{2}/n_{22}$, the stage 2 sample mean differences in partitions 1 and 2 are ${\bar{X}}_{21}∼N(\delta_{1},\tau_{21}^{2})$ and ${\bar{X}}_{22}∼N(\delta_{2},\tau_{22}^{2})$, respectively. Let $\sigma_{21}^{2}=4\sigma^{2}/m_{21}$ and $\sigma_{22}^{2}=4\sigma^{2}/m_{22}$, the stage 2 sample means in *S*_1_ and *S*_2_ are ${\bar{Y}}_{21}∼N(\theta_{1},\sigma_{21}^{2})$ and ${\bar{Y}}_{22}∼N(\theta_{2},\sigma_{22}^{2})$, respectively. For this hypothetical trial, $\tau_{21}^{2}=\tau_{22}^{2}=\sigma_{21}^{2}=1.633$ and $\sigma_{22}^{2}=0.817$. Table 1 summarizes the notation we have introduced for any *K* ≥ 3. When a subscript in a notation includes two indices, the first corresponds to stage and the second to partition or subpopulation.

**Table 1:** Summary of notation

		Stage 1 Partitions					Stage 2 Partitions		
Measure	Subgroup	1	2	…	*K* − **1**	*K*	1	…	*s* **∈** **{1**,…,*K* **}**
Upper threshold		*c*_1_	*c*_2_	…	*c*_*K* − 1_	*c*_*K*_	*c*_1_	…	*c*_*s*_
Sample size	Partition	*n*_11_	*n*_12_	…	*n*_1,*K* − 1_	*n*_1*K*_	*n*_21_	…	*n*_2*S*_
	Subpopulation	*m*_11_	*m*_12_	…	*m*_1,*K* − 1_	*m*_1*K*_	*m*_21_	…	*m*_2*s*_
Sample variance	Partition	$\tau_{11}^{2}$	$\tau_{12}^{2}$	…	$\tau_{1,K-1}^{2}$	$\tau_{1K}^{2}$	$\tau_{21}^{2}$	…	$\tau_{2s}^{2}$
	Subpopulation	$\sigma_{11}^{2}$	$\sigma_{12}^{2}$	…	$\sigma_{1,K-1}^{2}$	$\sigma_{1K}^{2}$	$\sigma_{21}^{2}$	…	$\sigma_{2s}^{2}$
True mean	Partition	*δ*_1_	*δ*_2_	…	*δ*_*K* − 1_	*δ*_*K*_	*δ*_1_	…	*δ*_*s*_
	Subpopulation	*θ*_1_	*θ*_2_	…	*θ*_*K* − 1_	*θ*_*K*_	*θ*_1_	…	*θ*_*s*_
Sample mean	Partition	${\bar{X}}_{11}$	${\bar{X}}_{12}$	…	${\bar{X}}_{1,K-1}$	${\bar{X}}_{1K}$	${\bar{X}}_{21}$	…	${\bar{X}}_{2s}$
	Subpopulation	${\bar{Y}}_{11}$	${\bar{Y}}_{12}$	…	${\bar{Y}}_{1,K-1}$	${\bar{Y}}_{1K}$	${\bar{Y}}_{21}$	…	${\bar{Y}}_{2s}$

Suppose that, in stage 2, the observed sample mean differences in partitions 1 and 2 are ${\bar{x}}_{21}=3.0$ and ${\bar{x}}_{22}=2.4$. Consequently, the stage 2 observed sample mean difference for *S*_2_ is ${\bar{y}}_{22}=2.7$. The naive estimate for *θ*_2_ is the two‐stage sample mean difference for *S*_2_ given by ${\hat{\theta }}_{2,N}=(m_{12}{\bar{y}}_{12}+m_{22}{\bar{y}}_{22})/(m_{21}+m_{22})=2.614$. We describe in Section 2.4 that the naive estimates are biased because they ignore subpopulation selection. The aim of this paper is to develop estimators that adjust for subpopulation selection. The estimators are based on the selection rule described for the hypothetical trial, which we state for any *K* ≥ 3 partitions in the next section, and are conditional on the observed ordering of stage 1 data.

### 2.3 Selection rule

We derive estimators that are unbiased or with small bias conditional on the following specific selection rule. Other selection rules are considered in the discussion. Let *b* denote a futility boundary. The trial stops after stage 1 if ${\bar{y}}_{1i}<b$ for all *i*(*i* = 1,…,*K*). The trial continues to stage 2 with the full population (*S*_*K*_) if ${\bar{y}}_{1K}\geq b$ and with subpopulation *S*_*s*_ ∈ {1,…,*K* − 1} if ${\bar{y}}_{1s}\geq b$ and ${\bar{y}}_{1i}<b$ for all *i* ∈ {*s* + 1,…,*K*}. Thus, as shown in the supplementary material, subpopulation *S*_*s*_ $\left(s\in \{1,\text{⋯},K-1\}\right)$ is selected if $b\leq {\bar{y}}_{1s}<u$, where

$$u=\min\left\{\frac{p_{s+1}b-(\;p_{s+1}-p_{s}){\bar{x}}_{1,s+1}}{p_{s}},\frac{p_{s+2}b-\sum_{i=s+1}^{s+2}(\;p_{i}-p_{i-1}){\bar{x}}_{1i}}{p_{s}},\text{⋯},\frac{p_{K}b-\sum_{i=s+1}^{K}(\;p_{i}-p_{i-1}){\bar{x}}_{1i}}{p_{s}}\right\}.$$

Equivalently, subpopulation *S*_*s*_ $\left(s\in \{1,\text{⋯},K-1\}\right)$ is selected if for all *i* *′* ∈ {1,…,*s*}, $v_{i^{\prime }}\leq {\bar{x}}_{1i^{\prime }}<w_{i^{\prime }}$, where $v_{i^{\prime }}=\frac{1}{p_{i^{\prime }}-p_{i^{\prime }-1}}\left(p_{s}\cdot b-\sum_{\underset{i\neq i^{\prime }}{i=1}}^{s}(\;p_{i}-p_{i-1}){\bar{x}}_{1i}\right)$ and

$$w_{i^{\prime }}=\min\left\{\frac{p_{s+1}b-\sum_{\underset{i\neq i^{\prime }}{i=1}}^{s+1}(\;p_{i}-p_{i-1}){\bar{x}}_{1i}}{p_{i^{\prime }}-p_{i^{\prime }-1}},\frac{p_{s+2}b-\sum_{\underset{i\neq i^{\prime }}{i=1}}^{s+2}(\;p_{i}-p_{i-1}){\bar{x}}_{1i}}{p_{i^{\prime }}-p_{i^{\prime }-1}},\text{⋯},\frac{p_{K}b-\sum_{\underset{i\neq i^{\prime }}{i=1}}^{K}(\;p_{i}-p_{i-1}){\bar{x}}_{1i}}{p_{i^{\prime }}-p_{i^{\prime }-1}}\right\}.$$

### 2.4 Naive estimation

For the selected subpopulation *S*_*s*_ $\left(s\in \{1,\text{⋯},K\}\right)$, define *t*_*s*_ = *m*_1*s*_/(*m*_1*s*_ + *m*_2*s*_). The naive estimator for *θ*_*s*_ that ignores subpopulation selection is

(1) $${\hat{\theta }}_{s,N}=t_{s}{\bar{Y}}_{1s}+(1-t_{s}){\bar{Y}}_{2s}.$$

This is biased because the first term in (1) includes data used in the selection. Let $1_{\left[S_{s}\right]}$ and Prob(*S*_*s*_) denote the indicator and probability of selecting *S*_*s*_, respectively. The conditional bias is

(2) $$\text{Bias}({\hat{\theta }}_{s,N})=t_{s}\left\{\frac{\sum_{i=1}^{s}(\;p_{i}-p_{i-1})E\left[{\bar{X}}_{1i}1_{\left[S_{s}\right]}\right]}{p_{s}\cdot \text{Prob}(S_{s})}-\theta_{s}\right\}.$$

Using the joint density for ${\bar{X}}_{1}$ or ${\bar{Y}}_{1}$ to compute Prob(*S*_*s*_) and $\sum_{i=1}^{s}(\;p_{i}-p_{i-1})E\left[{\bar{X}}_{1i}1_{\left[S_{s}\right]}\right]$ is computationally time consuming because the limits of integration for each element in the vector depend on the values of the other elements. To overcome this, we use **Z** = (*Z*_1_,…,*Z*_*K*_)*′*, where $Z_{1}={\bar{X}}_{11}$ and $Z_{i^{\prime }}=\sum_{i=1}^{i^{\prime }}(\;p_{i}-p_{i-1}){\bar{X}}_{1i}$ (*i* *′* = 2,…,*K*). The density for **Z** and the expressions for Prob(*S*_*s*_) and $\sum_{i=1}^{s}(\;p_{i}-p_{i-1})E\left[{\bar{X}}_{1i}1_{\left[S_{s}\right]}\right]$ are provided in the supplementary material.

## 3 ESTIMATORS THAT ACCOUNT FOR SUBPOPULATION SELECTION

### 3.1 Unbiased estimators

#### 3.1.1 General principles of obtaining unbiased estimators

One technique to account for subpopulation selection is Rao‐Blackwellization. By the Rao‐Blackwell theorem, conditional on a sufficient and complete statistic based on stages 1 and 2 data, the expected value of a conditionally unbiased estimator from the stage 2 data is the uniformly minimum variance conditional unbiased estimator (UMVCUE). We consider two methods for obtaining unbiased estimators for *θ*_*s*_: deriving an UMVCUE for *θ*_*s*_ directly or, because the relationship between ***θ*** and ***δ*** is linear, deriving the UMVCUE for each *δ*_*i*_(*i* = 1,…,*s*) and using a linear function to obtain an unbiased (though not necessarily minimum variance) estimator for *θ*_*s*_. The latter builds on the work by Kimani et al.^7^ The former would involve correlated stage 1 statistics in the vector ${\bar{Y}}_{1}$ and builds on the work by Robertson et al.^17^

#### 3.1.2 Uniformly minimum variance unbiased estimator following the work of Robertson et al (2016a)

The UMVCUE for *θ*_*s*_ is the expected value of ${\bar{Y}}_{2s}$ conditional on a sufficient and complete statistic. As before, let ${\hat{\theta }}_{s,N}$ denote the naive estimator for *θ*_*s*_ given by expression (1) and *U* be as *u* in Section 2.3 with ${\bar{x}}_{1,s+1},\text{⋯},{\bar{x}}_{1K}$ replaced with ${\bar{X}}_{1,s+1},\text{⋯},{\bar{X}}_{1K}$. Following the work of Robertson et al,^17^ the UMVCUE for *θ*_*s*_ is

(3) $${\hat{\theta }}_{s,UMV}={\hat{\theta }}_{s,N}-\frac{\sigma_{2s}^{2}}{\sqrt{\sigma_{1s}^{2}+\sigma_{2s}^{2}}}\frac{\varphi \left(f(b)\right)-\varphi \left(f(U)\right)}{\Phi \left(f(b)\right)-\Phi \left(f(U)\right)},$$

where $f(b)=\frac{\sqrt{\sigma_{1s}^{2}+\sigma_{2s}^{2}}}{\sigma 1_{s}^{2}}\left({\hat{\theta }}_{s,N}-b\right)$, $f(U)=\frac{\sqrt{\sigma_{1s}^{2}+\sigma_{2s}^{2}}}{\sigma 1_{s}^{2}}\left({\hat{\theta }}_{s,N}-U\right)$, and *φ*(.) and Φ(.) denote the density and distribution functions of a standard normal, respectively.

#### 3.1.3 Unbiased estimator following the work of Kimani et al (2015)

The UMVCUE for $\delta_{i^{\prime }}$ (*i* *′* = 1,…,*s*) is the expected value of ${\bar{X}}_{2i^{\prime }}$ conditional on a sufficient and complete statistic. Let ${\hat{\delta }}_{i^{\prime },N}=(n_{1i^{\prime }}{\bar{X}}_{1i^{\prime }}+n_{2i^{\prime }}{\bar{X}}_{2i^{\prime }})/(n_{1i^{\prime }}+n_{2i^{\prime }})$ (*i* *′* = 1,…,*s*) denote the naive estimator for $\delta_{i^{\prime }}$. Furthermore, let $V_{i^{\prime }}$ and $W_{i^{\prime }}$ be as $v_{i^{\prime }}$ and $w_{i^{\prime }}$ in Section 2.3 with ${\bar{x}}_{11},\text{⋯},{\bar{x}}_{1K}$ replaced with ${\bar{X}}_{11},\text{⋯},{\bar{X}}_{1K}$. Following the work of Kimani et al,^7^ the UMVCUE for $\delta_{i^{\prime }}$ (*i* *′* = 1,…,*s*) is

$${\hat{\delta }}_{i^{\prime },UMVCUE}={\hat{\delta }}_{i^{\prime },N}-\frac{\tau_{2i^{\prime }}^{2}}{\sqrt{\tau_{1i^{\prime }}^{2}+\tau_{2i^{\prime }}^{2}}}\frac{\varphi \left(f(V_{i^{\prime }})\right)-\varphi \left(f(W_{i^{\prime }})\right)}{\Phi \left(f(V_{i^{\prime }})\right)-\Phi \left(f(W_{i^{\prime }})\right)},$$

where $f(V_{i^{\prime }})=\frac{\sqrt{\tau_{1i^{\prime }}^{2}+\tau_{2i^{\prime }}^{2}}}{\tau_{1i^{\prime }}^{2}}\left({\hat{\delta }}_{i^{\prime },N}-V_{i^{\prime }}\right)$ and $f(W_{i^{\prime }})=\frac{\sqrt{\tau_{1i^{\prime }}^{2}+\tau_{2i^{\prime }}^{2}}}{\tau_{1i^{\prime }}^{2}}\left({\hat{\delta }}_{i^{\prime },N}-W_{i^{\prime }}\right)$. Consequently, the unbiased estimator for *θ*_*s*_ is

(4) $${\hat{\theta }}_{s,U}=\sum_{i=1}^{s}\frac{(\;p_{i}-p_{i-1}){\hat{\delta }}_{i,UMVCUE}}{p_{s}}.$$

### 3.2 Bias‐adjusted estimators

#### 3.2.1 An overview of bias‐adjusted estimation

Another technique to account for subpopulation selection would be to utilize the fact that we can calculate bias of the naive estimate using expression (2). The naive estimate is then adjusted by subtracting the bias. However, expression (2) is a function of ***δ*** (or equivalently ***θ***), the vector of the unknown treatment effects. To overcome this, we estimate bias, and hence, bias‐adjusted estimators obtained in this way are not necessarily mean unbiased.

#### 3.2.2 Single‐iteration bias‐adjusted estimator

We consider two bias‐adjusted estimators. For the first one, the bias is estimated based on the observed sample mean differences ${\hat{\delta }}_{i,N}=\left(n_{1i}{\bar{x}}_{1i}+n_{2i}{\bar{x}}_{2i}1_{\left[i\leq s\right]}\right)/\left(n_{1i}+n_{2i}1_{\left[i\leq s\right]}\right)$ (*i* = 1,…,*K*). Let $\hat{\delta }={\left({\hat{\delta }}_{1,N},\text{⋯},{\hat{\delta }}_{K,N}\right)}^{\prime }$ and $b_{\theta_{s}}(\hat{\delta })$ denote the bias estimator for *θ*_*s*_ obtained by replacing ***δ*** with $\hat{\delta }$ in expression (2) to get an adjusted estimator for *θ*_*s*_ of

(5) $${\hat{\theta }}_{s,SI}={\hat{\theta }}_{s,MLE}-b_{\theta_{s}}(\hat{\delta }).$$

We will refer to this estimator as the single‐iteration bias‐adjusted estimator.

#### 3.2.3 Multiple‐iteration bias‐adjusted estimator

For the second bias‐adjusted estimator, the bias is estimated iteratively.^10^, ^13^, ^18^ Let ${\hat{\theta }}_{i}$ (*i* = 1,…,*K*) denote the naive estimator for *θ*_*i*_ and $\hat{\theta }={\left({\hat{\theta }}_{1},\text{⋯},{\hat{\theta }}_{K}\right)}^{\prime }$. The biases for the naive estimators depend on ***θ*** and we denote bias for ${\hat{\theta }}_{i}$ (*i* = 1,…,*K*) by *b*_*i*_(***θ***) and the vector $\left(b_{1}(\theta ),\text{⋯},b_{K}(\theta )\right)$ by ***b***(***θ***). The second adjusted estimator, which we refer to as multiple‐iteration bias‐adjusted estimator is obtained by solving $\tilde{\theta }=\hat{\theta }-b(\tilde{\theta })$ iteratively. Using similar notation, alternatively, one could solve $\tilde{\delta }=\hat{\delta }-b(\tilde{\delta })$ and then use the relationship between ***θ*** and ***δ*** to obtain a bias‐adjusted estimate for *θ*_*s*_. For the simulations in Section 5, we solve $\tilde{\delta }=\hat{\delta }-b(\tilde{\delta })$ and with an accuracy of 0.001, convergence was achieved in almost all simulated trials. Suppose that the solution is obtained at iteration ***r*** and let $b_{i}({\tilde{\delta }}_{r})$ denote the bias for ${\hat{\delta }}_{i}$ when ***δ*** is taken to be ${\tilde{\delta }}_{r}$, then the multiple‐iteration adjusted estimate for *δ*_*i*_ is ${\hat{\delta }}_{i,MI}={\hat{\delta }}_{i}-b_{i}({\tilde{\delta }}_{r})$ and the multiple‐iteration bias‐adjusted estimator for *θ*_*s*_ is

(6) $${\hat{\theta }}_{s,MI}=\sum_{i=1}^{s}\frac{(\;p_{i}-p_{i-1}){\hat{\delta }}_{i,MI}}{p_{s}}.$$

The details of calculating $b_{i}({\tilde{\delta }}_{r})$ are given in the supplementary materials.

### 3.3 Shrinkage estimators

#### 3.3.1 General principles for shrinkage estimation

A third technique for accounting for subpopulation selection is to use shrinkage methods. Hwang^19^ considered the case of estimating a treatment mean after ordering independent sample means in a single‐stage trial for *K* ≥ 4. A subpopulation selection rule that corresponds to Hwang's case is that of selecting only one partition based on some ordering of ${\bar{x}}_{11},\text{⋯},{\bar{x}}_{1K}$. We initially consider Hwang's selection rule and denote the selected partition by *s*_*H*_ $\left(s_{H}\in \{1,\text{⋯},K\}\right)$. Hwang assigns a common normal prior distribution *N*(*μ*,*ν* ^2^) to each *δ*_*i*_(*i* = 1,…,*K*). The posterior mean for $\delta_{s_{H}}$, its Bayes estimator, is $C{\bar{X}}_{1s_{H}}+(1-C)\mu$, where *C* = 1 − 2*σ* ^2^/(2*σ* ^2^ + *n* *ν* ^2^) and *n* is stage 1 sample size in each intervention in each partition. Replacing the unknown *μ* and *C* with their unbiased estimators ${\bar{Y}}_{1K}=\sum_{i=1}^{K}{\bar{X}}_{1j}/K$ and $Ĉ=1-2(K-3)\sigma^{2}/[n\sum_{j=1}^{K}{\left({\bar{X}}_{1j}-{\bar{Y}}_{1K}\right)}^{2}]$, respectively, gives the empirical Bayes estimator. Let ${Ĉ}_{+}=\max\{0,Ĉ\}$, Hwang indicates that a better estimator, which we refer to as the shrinkage estimator, is ${\hat{\delta }}_{s_{H},B_{1}}={Ĉ}_{+}{\bar{X}}_{1s_{H}}+(1-{Ĉ}_{+}){\bar{Y}}_{1K}$.

Carreras and Brannath^14^ extended the work to two‐stage trials. Define $t_{s_{H}}=n_{1s_{H}}/(n_{1s_{H}}+n_{2s_{H}})$ to be the proportion of stage 1 data. The two‐stage shrinkage estimator for $\delta_{s_{H}}$ is ${\hat{\delta }}_{s_{H},B}=t_{s_{H}}{\hat{\delta }}_{s_{H},B_{1}}+(1-t_{s_{H}}){\bar{X}}_{2S}$. For *K* < 4, Carreras and Brannath propose defining $Ĉ=1-2(K-1)\sigma^{2}/[n\sum_{i=1}^{K}{\left({\bar{X}}_{1i}-{\bar{Y}}_{1K}\right)}^{2}]$. Using the fact that the estimator of Hwang^19^ applies for all parameters *δ*_*i*_(*i* = 1,…,*K*) and that its examination by Carreras and Brannath showed that it works for any rule used to pick the parameters on which to make inference, in Sections 3.3.2 and 3.3.3, we extend this work to give two shrinkage estimators for the subpopulation selection rule in Section 2.3.

#### 3.3.2 First shrinkage estimator

As in unbiased estimation, we consider both combining shrinkage estimators for treatment effects in partitions to obtain an estimator for *θ*_*s*_ and directly obtaining a shrinkage estimator for *θ*_*s*_. From Section 3.3.1, the shrinkage estimator for *δ*_*i*_(*i* = 1,…,*s*) is ${\hat{\delta }}_{i,L}=t_{s}\left[{Ĉ}_{+}{\bar{X}}_{1i}+(1-{Ĉ}_{+}){\bar{Y}}_{1K}\right]+(1-t_{s}){\bar{X}}_{2i}$, where ${Ĉ}_{+}=\max\{0,Ĉ\}$ and for *K* ≥ 4, $Ĉ=1-2(K-3)\sigma^{2}/[n\sum_{j=1}^{K}{\left({\bar{X}}_{1j}-{\bar{Y}}_{1K}\right)}^{2}]$, whereas for *K* < 4, $Ĉ=1-2(K-1)\sigma^{2}/[n\sum_{j=1}^{K}{\left({\bar{X}}_{1j}-{\bar{Y}}_{1K}\right)}^{2}]$. The first shrinkage estimator for *θ*_*s*_ is

(7) $${\hat{\theta }}_{s,L_{1}}=\sum_{i=1}^{s}\frac{(\;p_{i}-p_{i-1}){\hat{\delta }}_{i,L}}{p_{s}}.$$

#### 3.3.3 Second shrinkage estimator

The second shrinkage estimator, which we denote by ${\hat{\theta }}_{s,L_{2}}$, involves using the entire parameter vector ***θ***. A multivariate normal prior for ***θ*** is specified and updated with the data ${\bar{Y}}_{1}$. The resulting posterior is multivariate normal with nonzero covariance, and hence, the iterative procedure of Morris^27^ and Brüncker et al^28^ is utilized to obtain ${\hat{\theta }}_{s,L_{2}}$ (see supplementary material).

## 4 WORKED EXAMPLE

We use data from the hypothetical trial for depression in Section 2.2 to demonstrate how to compute the naive ( ${\hat{\theta }}_{2,N}$), the UMVCUE ( ${\hat{\theta }}_{2,UMV}$), the unbiased ( ${\hat{\theta }}_{2,U}$), the single‐iteration bias‐adjusted ( ${\hat{\theta }}_{2,SI}$), the multiple‐iteration bias‐adjusted ( ${\hat{\theta }}_{2,MI}$), the first shrinkage ( ${\hat{\theta }}_{2,L_{1}}$), and the second shrinkage ( ${\hat{\theta }}_{2,L_{2}}$) estimates. We also use the example to demonstrate differences among the various estimates in a single trial. The data and the various estimates are summarized in Table 2. The explicit computations for the various estimates and the R program used are provided in the supplementary material. Here, we only give explicit details of computing ${\hat{\theta }}_{2,UMV}$ and ${\hat{\theta }}_{2,U}$ as they are easier to compute, and since based on the simulations in the next section, we recommend ${\hat{\theta }}_{2,UMV}$.

**Table 2:** Worked example data and estimates

Data and Summary Measures									
		Stage 1 Partitions				Stage 2 Partitions		Estimating ***θ***_2_	
Measure	Subgroup	1	2	3	4	1	*s* = 2	Estimator	Estimate
Sample	Partition	*n*_11_ = 90	*n*_12_ = 90	*n*_13_ = 90	*n*_14_ = 90	*n*_21_ = 120	*n*_22_ = 120	${\hat{\theta }}_{2,N}$	2.614
size	Subgroup	*m*_11_ = 90	*m*_12_ = 180	*m*_13_ = 270	*m*_14_ = 360	*m*_21_ = 120	*m*_22_ = 240	${\hat{\theta }}_{2,UMV}$	2.839
Sample	Partition	$\tau_{11}^{2}=2.178$	$\tau_{12}^{2}=2.178$	$\tau_{13}^{2}=2.178$	$\tau_{14}^{2}=2.178$	$\tau_{21}^{2}=1.633$	$\tau_{22}^{2}=1.633$	${\hat{\theta }}_{2,U}$	2.965
variance	Subgroup	$\sigma_{11}^{2}=2.178$	$\sigma_{12}^{2}=1.089$	$\sigma_{13}^{2}=0.726$	$\sigma_{14}^{2}=0.544$	$\sigma_{21}^{2}=1.633$	$\sigma_{22}^{2}=0.817$	${\hat{\theta }}_{2,SI}$	2.633
Sample	Partition	${\bar{x}}_{11}=3$	${\bar{x}}_{12}=2\;$	${\bar{x}}_{13}=0.8\;$	${\bar{x}}_{14}=0\;$	${\bar{x}}_{21}=3$	${\bar{x}}_{22}=2.4$	${\hat{\theta }}_{2,MI}$	2.666
mean	Subgroup	${\bar{y}}_{11}=3$	${\bar{y}}_{12}=2.5$	${\bar{y}}_{13}=1.93$	${\bar{y}}_{14}=1.45$	${\bar{y}}_{21}=3$	${\bar{y}}_{22}=2.7$	${\hat{\theta }}_{2,L_{1}}$	2.164
	
								${\hat{\theta }}_{2,L_{2}}$	2.194

For the UMVCUE ( ${\hat{\theta }}_{2,UMV}$) given by expression (3), the first term ${\hat{\theta }}_{s,N}={\hat{\theta }}_{2,N}=2.614$. Furthermore, $\sigma_{1s}^{2}=\sigma_{12}^{2}=1.089$ and $\sigma_{2s}^{2}=\sigma_{22}^{2}=0.817$ so that $\frac{\sigma_{2s}^{2}}{\sqrt{\sigma_{1s}^{2}+\sigma_{2s}^{2}}}=\frac{\sigma_{22}^{2}}{\sqrt{\sigma_{12}^{2}+\sigma_{22}^{2}}}=0.592$ and $\frac{\sqrt{\sigma_{1s}^{2}+\sigma_{2s}^{2}}}{\sigma_{1s}^{2}}=\frac{\sqrt{\sigma_{12}^{2}+\sigma_{22}^{2}}}{\sigma_{12}^{2}}=1.268$. Since ( *p*_*i*_ − *p*_*i* − 1_) = 0.25 for all *i* = 1,…,4, then *u* (the observed value for *U*) is given by

$$\begin{array}{cc} u & =\min\left\{\frac{p_{3}b-(\;p_{3}-p_{2}){\bar{x}}_{13}}{p_{2}},\frac{p_{4}b-\sum_{i=3}^{4}(\;p_{i}-p_{i-1}){\bar{x}}_{1i}}{p_{2}}\right\}\\ & =\min\left\{\frac{\left(0.75\times 2)-(0.25\times 0.8\right)}{0.5},\frac{2-[0.25\times (0.8+0)]}{0.5}\right\}=2.6. \end{array}$$

Note that *f* (*b*) = 1.268 × (2.614 − 2) = 0.779 and *f* (*u*) = 1.268 × (2.614 − 2.6) = 0.018, so that substituting into expression (3), ${\hat{\theta }}_{2,UMV}=2.839$.

For the unbiased estimator ( ${\hat{\theta }}_{2,U}$) given by expression (4), we make the following calculations. The naive estimates for partitions 1 and 2 are ${\hat{\delta }}_{1,N}=[(90\times 3)+(120\times 3)]/210=3$ and ${\hat{\delta }}_{2,N}=[(90\times 2)+(120\times 2.4)]/210=2.229$, respectively. Note that $\frac{\tau_{21}^{2}}{\sqrt{\tau_{11}^{2}+\tau_{21}^{2}}}=\frac{\tau_{22}^{2}}{\sqrt{\tau_{21}^{2}+\tau_{22}^{2}}}=0.837$ and $\frac{\sqrt{\tau_{11}^{2}+\tau_{21}^{2}}}{\tau_{11}^{2}}=\frac{\sqrt{\tau_{21}^{2}+\tau_{22}^{2}}}{\tau_{21}^{2}}=0.896$. Since *p*_*i*_ − *p*_*i* − 1_ = 0.25(*i* = 1,…,4), $v_{1}=\frac{1}{0.25}\left(p_{2}b-0.25\sum_{\underset{i\neq 1}{i=1}}^{2}{\bar{x}}_{1i}\right)$ = 4 × [(0.5 × 0.2) − (0.25 × 2)] = 2 and $v_{2}=\frac{1}{0.25}\left(p_{2}b-0.25\sum_{\underset{i\neq 2}{i=1}}^{2}{\bar{x}}_{1i}\right)=4\times [(0.5\times 0.2)-(0.25\times 3)]=1$, respectively. For partition 1,

$$\begin{array}{cc} w_{1} & =\min\left\{\frac{p_{3}b-0.25\sum_{\underset{i\neq 1}{i=1}}^{3}{\bar{x}}_{1i}}{0.25},\frac{p_{4}b-0.25\sum_{\underset{i\neq 1}{i=1}}^{4}{\bar{x}}_{1i}}{0.25}\right\}\\ & =\min\left\{\frac{\left(0.75\times 2)-0.25\times (2+0.8\right)}{0.25},\frac{2-0.25\times (2+0.8+0)}{0.25}\right\}=3.2. \end{array}$$

Similarly, for partition 2, *w*_2_ = 2.2. Then, for partition 1, *f* (*v*_1_) = 0.896 × (3 − 2) = 0.896 and *f* (*w*_1_) = 0.896 × (3 − 3.2) = −0.179, and for partition 2, *f* (*w*_2_) = 0.896 × (2.229 − 1) = 1.101 and *f* (*w*_2_) = 0.896 × (2.229 − 2.2) = 0.026. Now, we have all components required to obtain UMVCUEs for the effects in partitions 1 and 2, which give ${\hat{\delta }}_{1,UMVCUE}=3.272$ and ${\hat{\delta }}_{2,UMVCUE}=2.657$, respectively. The unbiased estimate is the weighted sum of the UMVCUEs in the partitions giving ${\hat{\theta }}_{2,U}=2.965$.

The estimates ${\hat{\theta }}_{2,UMV}$, ${\hat{\theta }}_{2,U}$, ${\hat{\theta }}_{2,SI}$, and ${\hat{\theta }}_{2,MI}$ are greater than ${\hat{\theta }}_{2,N}$ (see Table 2). This may be explained by the observation in Section 5.2 that, in some scenarios, the naive estimator is negatively biased. The estimate ${\hat{\theta }}_{2,SI}$ is slightly smaller than ${\hat{\theta }}_{2,MI}$. Again, this may be explained by an observation in Section 5.2 that, for all scenarios in the simulation study, on average, the single‐iteration bias‐adjusted estimator gives a smaller estimate than the multiple‐iteration estimator.

## 5 SIMULATIONS TO COMPARE THE VARIOUS ESTIMATORS

### 5.1 Simulations setting

To evaluate the properties of the various estimators, we conducted simulations with *σ* ^2^ = 1 and *b* = 0. We initially consider the case of *K* = 4 and *p*_*i*_ − *p*_*i* − 1_ = 0.25(*i* = 1,…,4). In all simulations, if the trial continues to stage 2, the combined stages 1 and 2 sample size is set to be 800. For example, if the stage 1 sample size is 400 patients, the stage 2 sample size is 400. The available patients in stage 1 are equally split among the four partitions and treatment arms. For example, with 400 patients in stage 1, in each partition, 50 patients are randomly allocated to each of the control and experimental treatment. Similarly, the patients available for testing in stage 2 are equally split among the partitions that continue to stage 2 and among the treatment arms. Hence, with 400 patients available in stage 2, if *F* is selected, the patient allocation in stage 2 is as in stage 1 with 400 patients. If *S*_2_ is selected so that two partitions are tested in stage 2, in each partition, 100 patients are randomly allocated to each of the control and experimental treatment. We perform simulations for three cases of stage 1 sample size (200, 400, and 600 patients). Taking the combined stages 1 and 2 to be 800 patients is justified in the supplementary material.

We consider seven scenarios with true treatment effects as summarized in Table 3. The selection rule and estimators developed are aimed at identifying predictive effects, but since we are estimating mean differences, the methods are valid with or without prognostic effects. If the biomarker has no predictive effect but has a prognostic effect, we are in a scenario of equal treatment effects in all partitions. Scenarios 1, 3, and 7 could be such cases. If there are prognostic and predictive effects, we are in a scenario of unequal treatment effects in partitions. Scenarios 2, 4, 5, and 6 could be such cases. In Scenarios 1 to 3, the right decision is to continue to stage 2 with *F*, but with decreasing probability of selecting *F*. The right decisions for Scenarios 4 to 6 are to continue with *S*_3_, *S*_2_, and *S*_1_, respectively. The ideal decision for Scenario 7 is to stop at stage 1. The probabilities for various decisions for different scenarios when stage 1 includes 200 patients (25 in each treatment arm in each partition) are also given in Table 3. These have been calculated using expressions in Section 2.4 and in the supplementary material. As expected, the probability of stopping the trial at stage 1 (last column) increases as the treatment effects in partitions become less than *b* in more partitions (from 0.007 for Scenario 1 to 0.482 for Scenario 7). In each of Scenarios 4 to 6, the probability of continuing with *F* is substantially larger than the probability of making the right decision, demonstrating that, in some configurations, decision making is challenging. In Section 5.2.1, simulations show that incorrect decisions tend to be made when observed means are substantially different from the true means and hence lead to bias.

**Table 3:** Treatment effects and probabilities of different decisions for the various scenarios in the simulation study (probabilities of correct decisions are in bold)

	Treatment Effect					Probability of a Decision (*n*_1_ = **200**)				
Scenario	***δ***_1_	***δ***_2_	***δ***_3_	***δ***_4_	Ideal Selection	*F*	*S*_3_	*S*_2_	*S*_1_	Stop
1	0.3	0.3	0.3	0.3	*F*	**0.983**	0.005	0.003	0.002	0.007
2	0.2	0.1	0.1	0.1	*F*	**0.812**	0.049	0.035	0.034	0.070
3	0.0	0.0	0.0	0.0	*F*	**0.500**	0.083	0.070	0.073	0.274
4	0.1	0.0	0.0	−0.2	*S*_3_	0.430	**0.179**	0.093	0.093	0.205
5	0.1	0.0	−0.2	−0.1	*S*_2_	0.362	0.112	**0.179**	0.115	0.232
6	0.1	−0.2	−0.1	−0.1	*S*_1_	0.298	0.098	0.104	**0.214**	0.286
7	−0.1	−0.1	−0.1	−0.1	Stop	0.240	0.083	0.087	0.108	**0.482**

Table 4 gives probabilities of various decisions when the stage 1 sample sizes are 400 and 600. For scenario 3, where treatment effects are equal in all partitions and equal to the futility boundary, the probabilities of various decisions are approximately equal for different stage 1 sample sizes. For the other scenarios, by comparing the probabilities in bold, the probability of making a correct decision increases with stage 1 sample size.

**Table 4:** Probabilities of different decisions for different stage 1 sample sizes for various scenarios in the simulation study (probabilities of correct decisions are in bold)

	Ideal	Probability of a Decision (*n*_1_ **=** **400**)					Probability of a Decision (*n*_1_ **=** **600**)				
Scenario	Selection	*F*	*S*_3_	*S*_2_	*S*_1_	Stop	*F*	*S*_3_	*S*_2_	*S*_1_	Stop
1	*F*	**0.9987**	0.0004	0.0002	0.0002	0.0005	**0.99988**	0.00004	0.00002	0.00002	0.00004
2	*F*	**0.8944**	0.0312	0.0212	0.0200	0.0332	**0.93711**	0.02033	0.01326	0.01213	0.01717
3	*F*	**0.5000**	0.0833	0.0698	0.0734	0.2735	**0.50000**	0.08333	0.06981	0.07342	0.27344
4	*S*_3_	0.4013	**0.2286**	0.0983	0.0971	0.1747	0.37973	**0.26859**	0.10095	0.09838	0.15235
5	*S*_2_	0.3085	0.1220	**0.2386**	0.1261	0.2048	0.27015	0.12853	**0.28802**	0.13147	0.18183
6	*S*_1_	0.2266	0.0977	0.1156	**0.2939**	0.2662	0.17916	0.09454	0.12250	**0.35893**	0.24487
7	Stop	0.1587	0.0724	0.0842	0.1157	**0.5690**	0.11034	0.06193	0.07895	0.11756	**0.63122**

For each of the seven scenarios and three different stage 1 sample sizes, we simulated stage 1 data for *N* = 1 000 000 trials. For each trial, the subpopulation with the largest simulated sample mean difference ≥0 continues to stage 2. If no subpopulation fulfills this, the trial stops. We consider estimation conditional on continuing to stage 2 and so bias and MSE for each estimator are evaluated based on simulated trials that continue to stage 2. Using ${\hat{\theta }}_{s,SI}$ for illustration, for each *s* $\left(s=1,\text{⋯},4\right)$, bias and MSE are calculated as $\text{bias}({\hat{\theta }}_{s,SI})=\sum_{i=1}^{N}({\hat{\theta }}_{i,SI}-\theta_{i})1_{\left[i=s\right]}/\sum_{i=1}^{N}1_{\left[i=s\right]}$ and $\text{MSE}({\hat{\theta }}_{s,SI})=\sum_{i=1}^{N}{\left({\hat{\theta }}_{i,SI}-\theta_{i}\right)}^{2}1_{\left[i=s\right]}/\sum_{i=1}^{N}1_{\left[i=s\right]}$.

### 5.2 Simulation results

#### 5.2.1 Comparing biases for the various estimators

**Figure 2:**
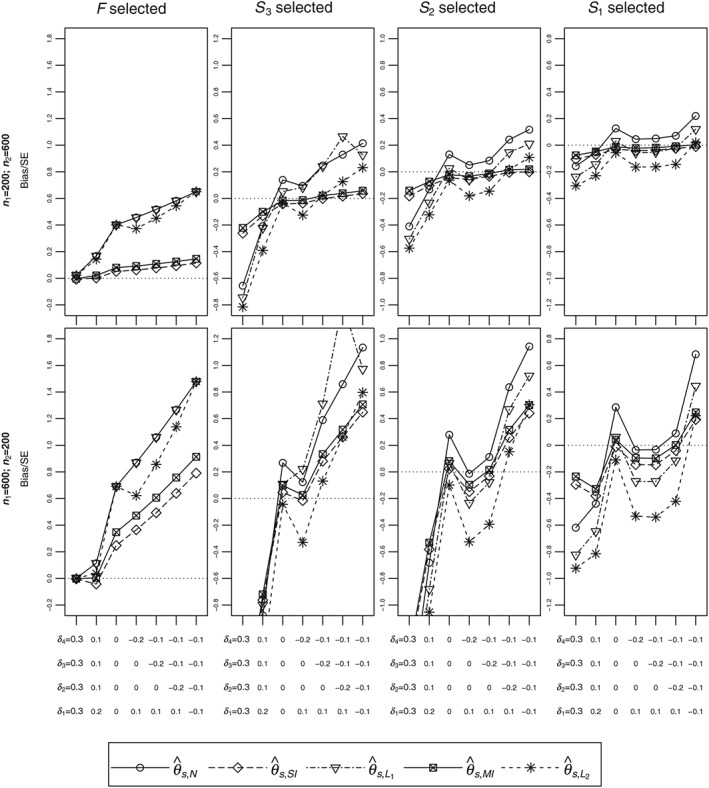
Biases in units of approximate standard error for different configurations. The dotted line is the point of no bias. Other line types correspond to different estimators. SE, standard error

Figure 2 summarizes biases when the stage 1 sample size is 200 (top plots) and 600 patients (bottom plots). Plots for the case where the stage 1 sample size is 400 patients are provided in the supplementary material. Plots in Columns 1 to 4 correspond to the cases of selecting *F*, *S*_3_, *S*_2_, and *S*_1_, respectively. The *y*‐axes correspond to biases divided by approximate standard errors (SEs). The approximate $\text{SE}=\sqrt{4/(m_{1s}+m_{2s})}$ and so SEs are only equal when *F* is selected (Column 1). Although SEs are not equal, we will later observe from the boxplots of the estimates that the trend for bias is the same when bias is not divided by SE. The *x*‐axes correspond to the seven scenarios. As per the legend, biases for different estimators are distinguished by different line types. Estimators ${\hat{\theta }}_{s,UMV}$ and ${\hat{\theta }}_{s,U}$ are not included in Figure 2 because they are mean unbiased. For Scenario 1, the probabilities for selecting *S*_3_, *S*_2_, and *S*_1_ are low and so simulations results are highly variable when *S*_3_, *S*_2_, or *S*_1_ is selected but this does not change the general findings in this paper.

We first describe the results for the case where the stage 1 sample size is 200 (top row). When F is selected, the naive estimator ( ${\hat{\theta }}_{s,N}$) and the first shrinkage estimator ( ${\hat{\theta }}_{s,L_{1}}$) are the same and correspond to the line showing the largest biases. Focusing on the naive estimator, the bias when F is selected (Column 1) is positive in all scenarios. For scenarios where the right decision is to continue with F (Scenarios 1 to 3, see Table 3), bias when F is selected is attributable to the futility rule with the bias negligible when the effect in F is substantially larger than the futility boundary (Scenario 1). When the right decision is not to continue with F (Scenarios 4 to 7) but F is selected, the impact of selection and futility on bias would increase and consequently give a larger bias. Still focusing on the top row, when S_3_ is selected (Column 2), the naive estimator for θ_3_ is negatively biased for some scenarios and positively biased for other scenarios. The explanation for this pattern is given in the supplementary material. Comparing the bias when F, S_3_, S_2_, and S_1_ are selected (Columns 1 to 4), the bias is smallest when S_1_ is selected. This can be attributed partly to the enrichment, where the stage 2 sample size is fixed regardless of the size of the population selected so that when S_1_ is selected, proportionally, there are more unbiased stage 2 data to estimate θ_1_ compared to when F, S_3_, or S_2_ is selected. In summary, note that, in some scenarios, the bias of the naive estimator is substantial and so it is essential to use an estimator that corrects for subpopulation selection.

Still focusing on the top row, when F is selected, practically, the single‐iteration bias corrected estimator ${\hat{\theta }}_{s,SI}$ is mean unbiased, especially for Scenarios 1 to 3 where the correct decision is to select F. When S_3_ is selected, ${\hat{\theta }}_{s,SI}$ almost eradicates bias in Scenarios 3 to 7 and is better than the naive estimator in Scenarios 1 and 2. When S_2_ or S_1_ is selected, ${\hat{\theta }}_{s,SI}$ eradicates almost all bias in Scenarios 2 to 7 but does not do so in Scenario 1. In all scenarios, the line for the multiple‐iteration bias‐adjusted estimator ( ${\hat{\theta }}_{s,MI}$) is always slightly above that of ${\hat{\theta }}_{s,SI}$. Hence, comparing ${\hat{\theta }}_{s,SI}$ and ${\hat{\theta }}_{s,MI}$, when ${\hat{\theta }}_{s,SI}$ is negatively biased, ${\hat{\theta }}_{s,MI}$ is preferable, whereas ${\hat{\theta }}_{s,SI}$ is preferable when it is positively biased.

Comparing biases for the naive estimator for different stage 1 sample sizes (top versus bottom plots), as also indicated by expression (2), the bias increases with the proportion of stage 1 data. Increase in bias is also seen for both the single‐iteration ( ${\hat{\theta }}_{s,SI}$) and multiple‐iteration ( ${\hat{\theta }}_{s,MI}$) bias‐adjusted estimators. From the bottom row, ${\hat{\theta }}_{s,SI}$ and ${\hat{\theta }}_{s,MI}$ perform worst when some partitions that should be dropped at stage 1 continue to stage 2 or when some partitions that should continue to stage 2 are dropped. As before, the line for ${\hat{\theta }}_{s,MI}$ is above that of ${\hat{\theta }}_{s,SI}$ with the distances between the lines increasing with stage 1 sample size.

The pattern of the shrinkage estimators is best understood by considering all results in Figure 2. In all cases, the line for the first shrinkage estimator ( ${\hat{\theta }}_{s,L_{1}}$) overlaps or is above that of the second shrinkage estimator ( ${\hat{\theta }}_{s,L_{2}}$). Estimator ${\hat{\theta }}_{s,L_{1}}$ performs similar to or better than ${\hat{\theta }}_{s,L_{2}}$ when the selected subpopulation consists of partitions that should continue to stage 2 such as when F is selected in Scenarios 1 to 3 and such as when S_3_ is selected in Scenarios 1 to 4. Estimator ${\hat{\theta }}_{s,L_{2}}$ performs better than ${\hat{\theta }}_{s,L_{1}}$ when the selected subpopulation consists of partitions that should not continue to stage 2 such as when F is selected in Scenarios 4 to 7 and such as when S_3_ is selected in Scenarios 5 to 7.

In almost all scenarios, the two shrinkage estimators perform worse than the other estimators that account for adaptation. One reason for this may be the fact that the shrinkage estimators do not account for stopping for futility. When F is selected, the naive estimator is the same as the first shrinkage estimator. This is because the stage 1 estimate in partition i is ${Ĉ}_{+}{\bar{X}}_{1i}+(1-{Ĉ}_{+}){\bar{Y}}_{1K}$ so that the shrinkage estimator shrinks to the effect in the full population, that is, to ${\bar{Y}}_{1K}=({\bar{X}}_{11}+\text{⋯}+{\bar{X}}_{1K})/K$. A reasonable alternative would be to use a weighted mean of ${\bar{Y}}_{11}$, ${\bar{Y}}_{12}$, …, ${\bar{Y}}_{1K}$. For example, if we shrink to $({\bar{Y}}_{11}+\text{⋯}+{\bar{Y}}_{1K})/K$, in terms of sample means in partitions, we are shrinking to a weighted sum such that for i < i ′, ${\bar{X}}_{1i}$ has more weight than ${\bar{X}}_{1i^{\prime }}$. In such a case, shrinkage estimators will be closer to the naive estimators when fewer partitions are selected (see additional simulations in the supplementary material).

#### 5.2.2 Comparing MSEs for the various estimators

**Figure 3:**
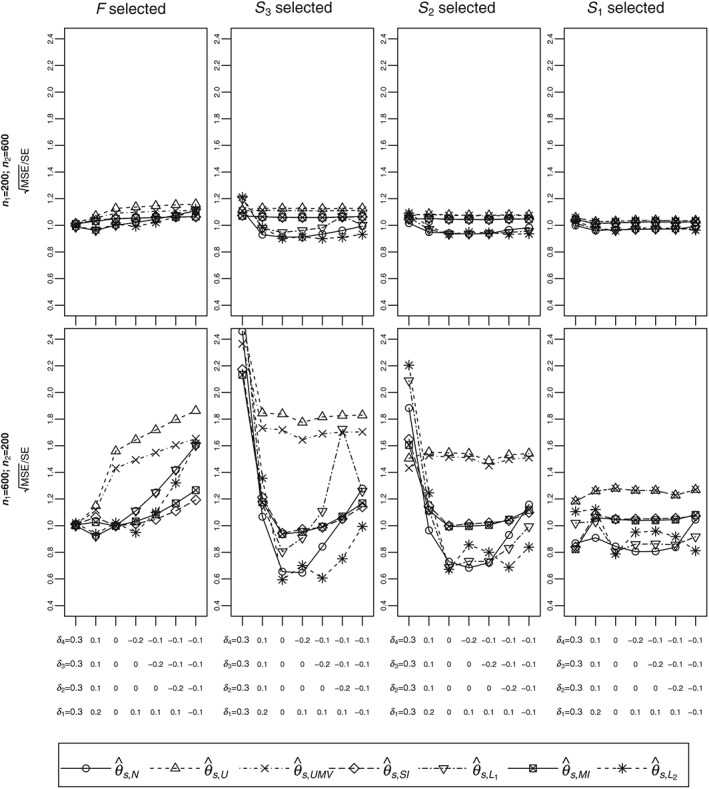
Root mean squares in units of approximate standard error for different configurations. Different line types correspond to different estimators. MSE, mean squared error; SE, standard error

Mean squared errors for the various estimators are given in Figure 3. The *y*‐axes are root mean squares ( $\text{RMSE}=\sqrt{\text{MSE}}$) divided by approximate SEs. The best shrinkage estimator in terms of bias (either ${\hat{\theta }}_{s,L_{1}}$ or ${\hat{\theta }}_{s,L_{2}}$ depending on the scenario) has smaller or practically the same MSEs as the naive estimator. Hence, the best shrinkage estimators may be considered to be better than the naive estimator in terms of MSE. The challenge, however, is determining the best shrinkage estimator since the true treatment means are unknown.

Since estimators that extend the works of Kimani et al ( ${\hat{\theta }}_{s,U}$) and of Robertson et al ( ${\hat{\theta }}_{s,UMV}$) are mean unbiased, their MSEs are variances. When *S*_1_ is selected, by derivation, the two estimators are the same and, hence, have equal MSE. For any other selection, as expected, ${\hat{\theta }}_{s,UMV}$ has smaller MSE than ${\hat{\theta }}_{s,U}$. The differences increase with stage 1 sample size (top versus bottom plots) and the size of the selected subpopulation (right to left panels). The MSEs of ${\hat{\theta }}_{s,U}$ and ${\hat{\theta }}_{s,UMV}$ are mostly larger than the MSEs for all the other estimators with the differences substantial when selection is performed later in the trial.

In general, the MSEs for the single‐iteration ( ${\hat{\theta }}_{s,SI}$) and multiple‐iteration ( ${\hat{\theta }}_{s,MI}$) bias‐adjusted estimators are practically the same. Hence, since their biases are also similar, the two estimators are approximately equivalent and so it is sufficient to compare one of them to the other estimators. The MSE for ${\hat{\theta }}_{s,SI}$ is larger than that of the naive estimator ( ${\hat{\theta }}_{s,N}$) in most cases while it is always smaller than the MSEs for the unbiased estimators ( ${\hat{\theta }}_{s,U}$ and ${\hat{\theta }}_{s,UMV}$).

#### 5.2.3 Comparing the estimators using both bias and MSE

Comparing the shrinkage estimators ( ${\hat{\theta }}_{s,L_{1}}$ and ${\hat{\theta }}_{s,L_{2}}$) to the naive estimator ( ${\hat{\theta }}_{s,N}$), we prefer ${\hat{\theta }}_{s,N}$. This is because although a shrinkage estimator sometimes has a smaller MSE, it can have substantially higher bias than ${\hat{\theta }}_{s,N}$ (for example, compare Columns 4 in Figures 2 and 3).

Comparing the single‐iteration bias‐adjusted estimator ( ${\hat{\theta }}_{s,SI}$) and the naive estimator ( ${\hat{\theta }}_{s,N}$), when *F* is selected, ${\hat{\theta }}_{s,SI}$ is preferable as it reduces bias substantially and has smaller MSE. However, when *S*_1_ is selected, ${\hat{\theta }}_{s,N}$ is better as it has smaller MSE and it does not differ from ${\hat{\theta }}_{s,SI}$ in terms of bias. When *S*_3_ or *S*_2_ is selected, ${\hat{\theta }}_{s,N}$ is better when bias is not substantial (Scenarios 3 and 4), whereas for Scenarios 5 to 7, ${\hat{\theta }}_{s,SI}$ is better as it reduces bias and its MSE is better or only slightly higher than that of ${\hat{\theta }}_{s,N}$. Overall, we consider ${\hat{\theta }}_{s,SI}$ as a better estimator than ${\hat{\theta }}_{s,N}$ as it performs better in cases with substantial bias.

When *F* is selected, the bias of the naive estimator ( ${\hat{\theta }}_{s,N}$) is substantial and compared to the UMVCUE ( ${\hat{\theta }}_{s,UMV}$), we prefer the latter since the difference in RMSE between the two estimators is smaller than the bias eradicated. When *S*_1_ is selected, we would also recommend ${\hat{\theta }}_{s,UMV}$ over ${\hat{\theta }}_{s,N}$ as the former is mean unbiased in all scenarios, with the only case where it is not clearly superior due to high RMSE being when *n*_1_ = 600. The conclusion when *S*_3_ or *S*_2_ is selected is the same as when *S*_1_ is selected, that is, ${\hat{\theta }}_{s,UMV}$ is better than ${\hat{\theta }}_{s,N}$.

Comparing the single‐iteration bias‐adjusted estimator ${\hat{\theta }}_{s,SI}$ to the UMVCUE ${\hat{\theta }}_{s,UMV}$, we recommend the latter since, when *F* is selected, ${\hat{\theta }}_{s,SI}$ has substantial bias that is larger than the difference in RMSE between it and ${\hat{\theta }}_{s,UMV}$. In addition, when *S*_1_ is selected, the difference in RMSE between the two estimators is smaller than the bias of ${\hat{\theta }}_{s,SI}$. Consequently, based on the performance across the scenarios in the simulation study, we recommend ${\hat{\theta }}_{s,UMV}$ when an adaptive threshold enrichment design is used.

**Figure 4:**
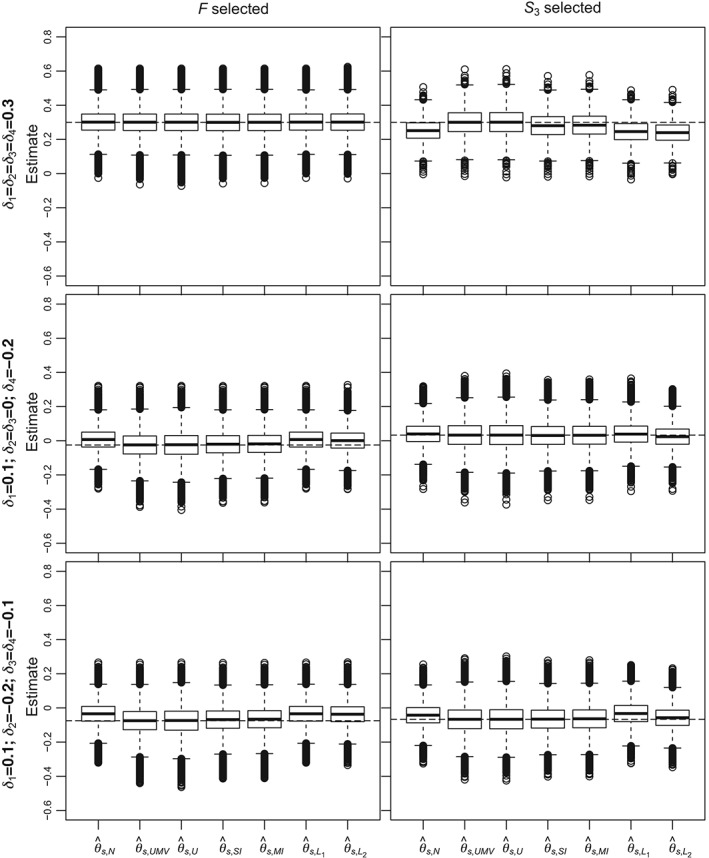
Boxplots of estimates for different estimators when n_1_ = 200. Results have been chosen when F and S_3_ were selected and for Scenarios 1 (top panels), 4 (middle panels), and 6 (bottom panels). The dashed lines correspond to the true means in the selected subpopulation

**Figure 5:**
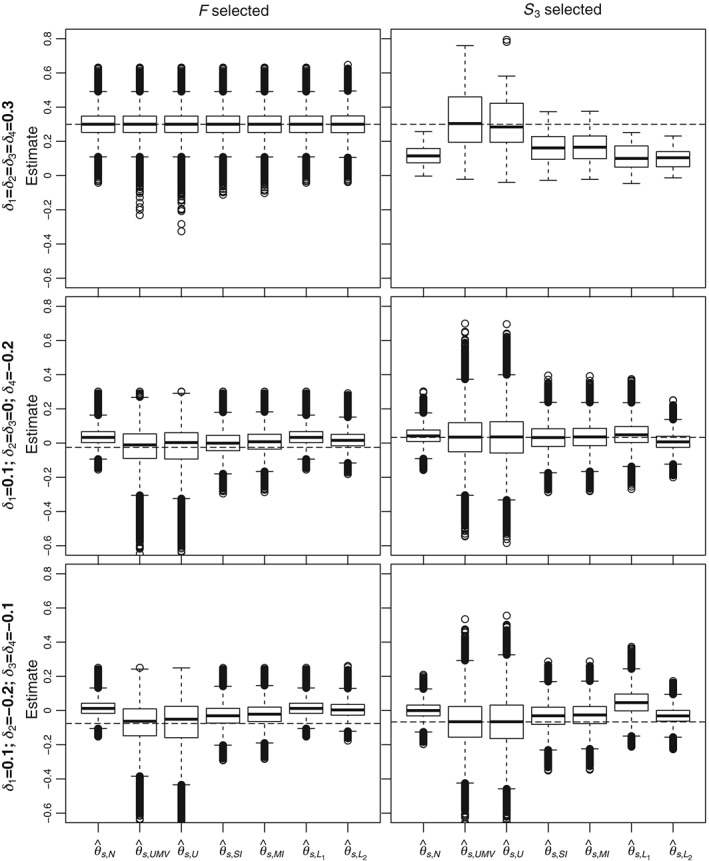
Boxplots of estimates for different estimators when n_1_ = 600. Results have been chosen when F and S_3_ were selected and for Scenario 1 (top panels), 4 (middle panels), and 6 (bottom panels). The dashed lines correspond to the true means in the selected subpopulation

For a more detailed comparison of the estimators, Figures 4 and 5 give boxplots of simulated estimates for Scenarios 1 (top plots), 4 (middle plots), and 6 (bottom plots) described in Table 3 when *F* and *S*_3_ are selected. The boxplots emphasize the findings summarized above. As an example, when *n*_1_ = 600(Figure 5), for Scenario 6 (bottom left panel), almost all naive estimates are above the true value and ${\hat{\theta }}_{s,UMV}$ performs well in that case. From the left panels, we note the unbiased estimators ( ${\hat{\theta }}_{s,UMV}$ and ${\hat{\theta }}_{s,U}$) have substantially higher variances compared to the other estimators.

#### 5.2.4 Summary findings and recommendations from the simulation study

The bias of the naive estimator can be substantial, and so it is essential to use an estimator that corrects for the decision made using stage 1 data. We recommend the estimator that follows the work of Robertson et al ( ${\hat{\theta }}_{s,UMV}$) since it is mean unbiased. Although it has larger MSE than some estimators, the bias eradicated in most cases was larger than the difference in RMSEs. Although the simulation study was based on four partitions and specific treatment effect scenarios, we expect similar findings for other configurations (that is, more candidate partitions and/or different effect sizes). Simulations for the case of 8 partitions are in the supplementary material.

We have recommended one estimator for all scenarios. An alternative is a hybrid estimator where the recommended estimator ( ${\hat{\theta }}_{s,SI}$ or ${\hat{\theta }}_{s,UMV}$) depends on the subpopulation selected. This is suitable if investigators are willing to sacrifice unbiasedness for more precision. In this case, before the trial, a simulation study based on plausible scenarios would be required to compare bias and MSE conditional on the selected subpopulation.

## 6 DISCUSSION

Acknowledging that different patients may require different care has led to trial designs that incorporate assessment of treatment effects in different subsets of the population. Most statistical methodologies for such designs focus on hypothesis testing.^2^, ^5^, ^24^, ^26^, ^29^, ^30^, ^31^, ^32^, ^33^ In this paper, we have considered point estimation following an adaptive threshold enrichment clinical trial. We have assessed bias for the naive estimator when different subpopulations are selected. Depending on the scenario, the bias of the naive estimator of the treatment effect in the selected subpopulation is substantial and can be negative or positive. There is thus a need for new estimators. Building on estimators that have been proposed for treatment selection, we have derived several estimators that account for subpopulation selection. By derivation, two estimators are mean unbiased. In this paper, we have recommended the best among these two, that is, the UMVCUE. An alternative is a hybrid estimator where different estimators are recommended based on the selected subpopulation. This would require a simulation study before the trial and is suitable if investigators can accept some unbiasedness for a more precise estimator.

We have considered a specific selection rule but the proposed estimators can be modified for other selection rules. For example, it may be desired that different subpopulations have different futility boundaries. Futility boundaries may be based on factors such as subpopulation prevalence, and sponsor and public health gains.^34^ Another factor is safety where the futility boundary may be chosen to reflect investigators' willingness to accept moderate efficacy if the new treatment is substantially safer than the control. The selection rule we have used specifies that a higher biomarker value leads to a smaller treatment effect. If this is a misspecification of the relationship between the biomarker and treatment effect, the unbiased estimators will remain so because we condition on the selection rule. However, the probability of making the right decision will be low and we anticipate that the naive estimator will have more bias and that the unbiased estimators will have higher MSE.

In the derivations, we have not required the prevalences in different partitions to be equal. If the biomarker values are approximately continuous, then it is reasonable to subdivide the full population into equal partitions as we have done in the example and the simulations. Other numerical biomarker values may be discrete with few possible values, leading to partitions with varying sizes.

We have assumed the number of patients in each partition, and hence prevalence, is known. For the case of two partitions and a fixed cut‐off value, taking the stage 1 number of patients in a partition to have a binomial distribution, Kimani et al^7^ showed that using stage 1 prevalence estimates in the expressions for the unbiased estimators provides unbiased estimates for the treatment effects. This extends to the case of more than two partitions, where numbers of patients in partitions are taken to have a multinomial distribution. The proof is based on the fact that the estimator in a partition is unbiased conditional on the number of patients in an interval and that the proportion of patients in a partition is unbiased for the prevalence in the partition. The proof for the case of estimating the cut‐off values using stage 1 data is similar.

Conditional on continuing to stage 2, we have derived estimators for the effect in the selected subpopulation. Continuing to stage 2 is necessary for the unbiased estimators. This is not the case for the other estimators as they involve obtaining stage 1 estimates in all partitions that correct for the subpopulation selection and then combine them with the stage 2 unbiased estimates. Hence, estimates for effects in the dropped partitions that correct for subpopulation selection can be obtained using the shrinkage and bias‐adjusted estimators. However, they are not necessarily mean unbiased.

Methods developed for normally distributed data following treatment selection have been adapted for time‐to‐event data.^28^ Even after assuming asymptotic normality of the log hazard ratio, some of the estimators we have derived such as the UMVCUE may not be valid for time‐to‐event data. For example, if there is a quantitative interaction with hazard ratios in different partitions being unequal, a model that accounts for this is required. In this case, obtaining separate estimates for each partition is the valid approach.

Finally, since in all simulations, the combined stages 1 and 2 sample size was 800, for the different stage 1 sample sizes considered, there would be no savings or losses in terms of the cost of treating patients. The saving/loss is only made in terms of costs associated with biomarker testing. Hence, the case for performing subpopulation selection with a small proportion of patients can be justified if the biomarker is expensive, leading to savings if *F* is selected. The case for performing subpopulation selection with a large proportion of patients is justifiable if the biomarker is not expensive. In this case, the resources loss is not substantial if *F* is selected and yet, if only a part of the population will benefit, there is a higher probability of making the right decision that may improve power. The setting of fixed total sample size is sometimes referred to as enrichment because if some partitions are dropped in stage 2, the number of patients recruited from partitions in stage 2 is higher than if more partitions were selected. To save money on treatment costs or reduce the total sample size, subpopulation selection could be performed early, with no enrichment in stage 2. With no enrichment, the number of patients in a partition in stage 2 is fixed. The statistical properties of the estimators for the setting with no enrichment can be evaluated as in the case of enrichment.

## Supporting information

SIM7831‐sup‐0001‐supplementary information.pdf
