# Supplementary material for: Point estimation following two‐stage adaptive threshold enrichment clinical trials
Source: Stat Med. 2018 May 31;37(22):3179–96. doi: 10.1002/sim.7831 (PMC6175016; doi:10.1002/sim.7831)
Supplement: Supplementary file 1 — SIM7831‐sup‐0001‐supplementary information.pdf [file SIM-37-3179-s001.pdf]

Point estimation following two-stage adaptive threshold enrichment clinical trials

Supplementary material

1 The relationship between $\bar{\mathbf{X}}_1$ and $\bar{\mathbf{Y}}_1$ and the density for $\bar{\mathbf{Y}}_1$

Note that since components of the vector of partition sample means $\bar{\mathbf{X}}_1 = (\bar{X}_{11}, \bar{X}_{12}, \cdots, \bar{X}_{1K})'$ are independent, $\bar{\mathbf{X}}_1$ is multivariate normal $MVN(\boldsymbol{\delta}, \Sigma_X)$, where $\boldsymbol{\delta} = (\delta_1, \delta_2, \cdots, \delta_K)'$ and Σ_X is the $K \times K$ diagonal matrix whose i^{th} diagonal element is $\tau_{1i}^2 = 4\sigma^2/n_{1i}$.

Let $\mathbf{A}$ be the $K \times K$ matrix where, if $i < i'$ or $i > i'+1$, the ii'th entry is zero, if $i = i'$, the ii'th entry is $\frac{p_{i'}}{p_{i'} - p_{i'-1}}$, and if $i = i'+1$, the ii'th entry is $\frac{-p_{i'}}{p_i - p_{i'}}$, that is

$$\mathbf{A} = \begin{pmatrix} 1 & 0 & 0 & 0 & \cdots & 0 & 0 & 0 \\ \frac{-p_1}{p_2-p_1} & \frac{p_2}{p_2-p_1} & 0 & 0 & \cdots & 0 & 0 & 0 \\ 0 & \frac{-p_2}{p_3-p_2} & \frac{p_3}{p_3-p_2} & 0 & \cdots & 0 & 0 & 0 \\ \cdot & \cdot & \cdot & \cdot & \cdots & \cdot & \cdot & \cdot \\ \cdot & \cdot & \cdot & \cdot & \cdots & \cdot & \cdot & \cdot \\ \cdot & \cdot & \cdot & \cdot & \cdots & \cdot & \cdot & \cdot \\ 0 & 0 & 0 & 0 & \cdots & \frac{-p_{K-2}}{p_{K-1}-p_{K-2}} & \frac{p_{K-1}}{p_{K-1}-p_{K-2}} & 0 \\ 0 & 0 & 0 & 0 & \cdots & 0 & \frac{-p_{K-1}}{1-p_{K-1}} & \frac{1}{1-p_{K-1}} \end{pmatrix}.$$

Then $\mathbf{B} = \mathbf{A}^{-1}$, is the $K \times K$ matrix where, if $i < i'$, the ii'th entry is zero, and if $i \geq i'$, the ii'th entry is $\frac{p_{i'} - p_{i'-1}}{p_i}$, that is

$$\mathbf{B} = \begin{pmatrix} 1 & 0 & 0 & 0 & \cdots & 0 & 0 & 0 \\ \frac{p_1}{p_2} & \frac{p_2-p_1}{p_2} & 0 & 0 & \cdots & 0 & 0 & 0 \\ \frac{p_1}{p_3} & \frac{p_2-p_1}{p_3} & \frac{p_3-p_2}{p_3} & 0 & \cdots & 0 & 0 & 0 \\ \cdot & \cdot & \cdot & \cdot & \cdots & \cdot & \cdot & \cdot \\ \cdot & \cdot & \cdot & \cdot & \cdots & \cdot & \cdot & \cdot \\ \cdot & \cdot & \cdot & \cdot & \cdots & \cdot & \cdot & \cdot \\ \frac{p_1}{p_{K-1}} & \frac{p_2-p_1}{p_{K-1}} & \frac{p_3-p_2}{p_{K-1}} & \frac{p_4-p_3}{p_{K-1}} & \cdots & \frac{p_{K-2}-p_{K-1}}{p_{K-1}} & \frac{p_{K-1}-p_{K-2}}{p_{K-1}} & 0 \\ p_1 & p_2-p_1 & p_3-p_2 & p_4-p_3 & \cdots & p_{K-2}-p_{K-3} & p_{K-1}-p_{K-2} & 1-p_{K-1} \end{pmatrix}.$$

Let $\boldsymbol{\theta} = (\theta_1, \theta_2, \cdots, \theta_K)'$ and $\bar{\mathbf{Y}}_1 = (\bar{Y}_{11}, \bar{Y}_{12}, \cdots, \bar{Y}_{1K})'$. Then $\boldsymbol{\delta} = \mathbf{A}\boldsymbol{\theta}$, $\boldsymbol{\theta} = \mathbf{B}\boldsymbol{\delta}$, $\bar{\mathbf{X}}_1 = \mathbf{A}\bar{\mathbf{Y}}_1$ and $\bar{\mathbf{Y}}_1 = \mathbf{B}\bar{\mathbf{X}}_1$. Consequently, $\bar{\mathbf{Y}}_1 \sim MVN(\boldsymbol{\theta}, \Sigma_Y)$, where $\Sigma_Y = \mathbf{B}\Sigma_X\mathbf{B}^T$.

2 Selection rule

2.1 In terms of subpopulation sample means $\bar{y}_{11}, \ldots \bar{y}_{1,K-1}$

We use three examples to demonstrate the condition for selecting a subpopulation.

Case of selecting S_{K-1}
Subpopulation S_{K-1} is selected if $\bar{y}_{1,K-1} \geq b$ and $\bar{y}_{1K} < b$. The latter can be re-expressed as follows

$$\bar{y}_{1K} = \frac{p_{K-1}\bar{y}_{1,K-1} + (p_K - p_{K-1})\bar{x}_{1K}}{p_K} < b \Rightarrow \bar{y}_{1,K-1} < \frac{p_K b - (p_K - p_{K-1})\bar{x}_{1K}}{p_{K-1}}$$

so that S_{K-1} is selected if

$$b \leq \bar{y}_{1,K-1} < \frac{p_K b - (p_K - p_{K-1})\bar{x}_{1K}}{p_{K-1}}.$$

Case of selecting S_{K-2}
Subpopulation S_{K-2} is selected if $\bar{y}_{1,K-2} \geq b$, $\bar{y}_{1,K-1} < b$ and $\bar{y}_{1K} < b$. Now

$$\bar{y}_{1K} = \frac{p_{K-2}\bar{y}_{1,K-2} + \sum_{i=s+1}^{K}(p_i - p_{i-1})\bar{x}_{1i}}{p_K} < b \Rightarrow \bar{y}_{1,K-2} < \frac{p_K b - \sum_{i=K-1}^{K}(p_i - p_{i-1})\bar{x}_{1i}}{p_{K-2}}$$

and

$$\bar{y}_{1,K-1} = \frac{p_{K-2}\bar{y}_{1,K-2} + (p_{K-1} - p_{K-2})\bar{x}_{1,K-1}}{p_{K-1}} < b \Rightarrow \bar{y}_{1,K-2} < \frac{p_{K-1} b - (p_{K-1} - p_{K-2})\bar{x}_{1,K-1}}{p_{K-2}}$$

so that S_{K-2} is selected if

$$b \leq \bar{y}_{1,K-2} < \min\left\{\frac{p_{K-1} b - (p_{K-1} - p_{K-2})\bar{x}_{1,K-1}}{p_{K-2}}, \frac{p_K b - \sum_{i=s+1}^{K}(p_i - p_{i-1})\bar{x}_{1i}}{p_{K-2}}\right\}.$$

Case of selecting S_{K-3}
Subpopulation S_{K-3} is selected if $\bar{y}_{1,K-3} \geq b$, $\bar{y}_{1,K-2} < b$, $\bar{y}_{1,K-1} < b$ and $\bar{y}_{1K} < b$. Now

$$\bar{y}_{1K} = \frac{p_{K-3}\bar{y}_{1,K-3} + \sum_{i=K-2}^{K}(p_i - p_{i-1})\bar{x}_{1i}}{p_K} < b \Rightarrow \bar{y}_{1,K-3} < \frac{p_K b - \sum_{i=K-2}^{K}(p_i - p_{i-1})\bar{x}_{1i}}{p_{K-3}},$$

$$\bar{y}_{1,K-1} = \frac{p_{K-3}\bar{y}_{1,K-3} + \sum_{i=K-1}^{K-2}(p_i - p_{i-1})\bar{x}_{1i}}{p_{K-1}} < b \Rightarrow \bar{y}_{1,K-3} < \frac{p_{K-1} b - \sum_{i=K-1}^{K-2}(p_i - p_{i-1})\bar{x}_{1i}}{p_{K-3}}$$

and

$$\bar{y}_{1,K-2} = \frac{p_{K-3}\bar{y}_{1,K-3} + (p_{K-2} - p_{K-3})\bar{x}_{1,K-2}}{p_{K-2}} < b \Rightarrow \bar{y}_{1,K-3} < \frac{p_{K-2} b - (p_{K-2} - p_{K-3})\bar{x}_{1,K-2}}{p_{K-3}}$$

so that if u^* is given by

$$\min\left\{\frac{p_{K-2}b-(p_{K-2}-p_{K-3})\bar{x}_{1,K-2}}{p_{K-3}},\frac{p_{K-2}b-(p_{K-2}-p_{K-3})\bar{x}_{1,K-2}}{p_{K-3}},\frac{p_Kb-\sum_{i=K-2}^{K}(p_i-p_{i-1})\bar{x}_{1i}}{p_{K-3}}\right\}$$

S_{K-3} is selected if $b\le\bar{y}_{1,K-3}<u^*$.

From these cases of selecting S_{K-1}, S_{K-2} and S_{K-3}, it is clear that the selection rule is as given in the main paper.

2.2 In terms of partition sample means $\bar{x}_{11},\ldots\bar{x}_{1,K-1}$

We demostrate the selection rule based on selection of S_{K-2}. Subpopulation S_{K-2} is selected if $\bar{y}_{1K}<b$, $\bar{y}_{1,K-1}<b$ and $\bar{y}_{1,K-2}\ge b$. For i $(i=1,\ldots,K)$

$$\bar{y}_{1K}=\frac{(p_i-p_{i-1})\bar{x}_{1i}+\sum_{\substack{j=1\\j\neq i}}^{K}(p_j-p_{j-1})\bar{x}_{1j}}{p_K}<b\;\Rightarrow\;\bar{x}_{1i}<\frac{p_Kb-\sum_{\substack{j=1\\j\neq i}}^{K}(p_j-p_{j-1})\bar{x}_{1j}}{p_i-p_{i-1}}.$$

For i $(i=1,\ldots,K-1)$,

$$\bar{y}_{1,K-1}=\frac{(p_i-p_{i-1})\bar{x}_{1i}+\sum_{\substack{j=1\\j\neq i}}^{K-1}(p_j-p_{j-1})\bar{x}_{1j}}{p_{K-1}}<b\;\Rightarrow\;\bar{x}_{1i}<\frac{p_{K-1}b-\sum_{\substack{j=1\\j\neq i}}^{K-1}(p_j-p_{j-1})\bar{x}_{1j}}{p_i-p_{i-1}}.$$

For i $(i=1,\ldots,K-2)$,

$$\bar{y}_{1,K-2}=\frac{(p_i-p_{i-1})\bar{x}_{1i}+\sum_{\substack{j=1\\j\neq i}}^{K-2}(p_j-p_{j-1})\bar{x}_{1j}}{p_{K-2}}\ge b\;\Rightarrow\;\bar{x}_{1i}\ge\frac{p_{K-2}b-\sum_{\substack{j=1\\j\neq i}}^{K-2}(p_j-p_{j-1})\bar{x}_{1j}}{p_i-p_{i-1}}.$$

The three conditions mean that subpopulation S_{K-2} is selected if for all $i'\{1,\ldots,K-1\}$, $v_{i'}\le\bar{x}_{1i'}<w_{i'}$, where $v_{i'}=\frac{1}{p_{i'}-p_{i'-1}}\left(p_{K-2}\cdot b-\sum_{\substack{i=1\\i\neq i'}}^{K-2}(p_i-p_{i-1})\bar{x}_{1i}\right)$ and

$$w_{i'}=\min\left\{\frac{p_{K-1}b-\sum_{\substack{i=1\\i\neq i'}}^{K-1}(p_{i'}-p_{i'-1})\bar{x}_{1i}}{p_{i'}-p_{i'-1}},\frac{p_Kb-\sum_{\substack{i=1\\i\neq i'}}^{K}(p_{i'}-p_{i'-1})\bar{x}_{1i}}{p_{i'}-p_{i'-1}}\right\}.$$

3 Expression for bias based on the density for Z

We define $\mathbf{Z} = (Z_1,..,Z_K)'$, where $Z_1 = \bar{X}_{11}$ and $Z_{i'} = \sum_{i=1}^{i'}(p_i - p_{i-1})\bar{X}_{1i}$ $(i' = 2,..,K)$. Note that S_1 is selected if $z_1 \geq b$ and for all i' $(i' = 2,...,K)$, $z_{i'} < p_{i'}b$. For $i' \geq 2$ $(i' = 2,....,K)$, $S_{i'}$ is selected for any value of z_i $(i < i')$ and if $z_{i'} \geq b$ and for i $(i > i')$, $z_i < p_i b$. Since $\mathbf{Z}$ is a linear transformation of $\bar{\mathbf{X}}_1$, $\mathbf{Z}$ is multivariate normal with mean vector $(\delta_1, \sum_{i=1}^{2}(p_i - p_{i-1})\delta_i, \sum_{i=1}^{3}(p_i - p_{i-1})\delta_i, ..., \sum_{i=1}^{K}(p_i - p_{i-1})\delta_i)'$ and variance covariance matrix

$$\Sigma_{\mathbf{Z}} = \begin{pmatrix} \tau_{11}^2 & p_1\tau_{11}^2 & p_1\tau_{11}^2 & . & . & p_1\tau_{11}^2 \\ p_1\tau_{11}^2 & \sum_{i=1}^{2}(p_i - p_{i-1})^2\tau_{1i}^2 & \sum_{i=1}^{2}(p_i - p_{i-1})^2\tau_{1i}^2 & . & . & \sum_{i=1}^{2}(p_i - p_{i-1})^2\tau_{1i}^2 \\ p_1\tau_{11}^2 & \sum_{i=1}^{2}(p_i - p_{i-1})^2\tau_{1i}^2 & \sum_{i=1}^{3}(p_i - p_{i-1})^2\tau_{1i}^2 & . & . & \sum_{i=1}^{3}(p_i - p_{i-1})^2\tau_{1i}^2 \\ . & . & . & . & & . \\ . & . & . & . & & . \\ p_1\tau_{11}^2 & \sum_{i=1}^{2}(p_i - p_{i-1})^2\tau_{1i}^2 & \sum_{i=1}^{3}(p_i - p_{i-1})^2\tau_{1i}^2 & . & . & \sum_{i=1}^{K}(p_i - p_{i-1})^2\tau_{1i}^2 \end{pmatrix}.$$

Let $f(\mathbf{z})$ denote the density for $\mathbf{Z}$. Then, for example,

$$\text{Prob}(S_1) = \int_b^\infty \int_{-\infty}^{p_2 b} \int_{-\infty}^{p_3 b} \cdots \int_{-\infty}^{p_{K-1}b} \int_{-\infty}^{b} f(\mathbf{z}) dz_K dz_{K-1} \cdots dz_3 dz_2 dz_1$$

and

$$\text{Prob}(S_2) = \int_{-\infty}^\infty \int_{p_2 b}^{\infty} \int_{-\infty}^{p_3 b} \cdots \int_{-\infty}^{p_{K-1}b} \int_{-\infty}^{b} f(\mathbf{z}) dz_K dz_{K-1} \cdots dz_3 dz_2 dz_1.$$

The integrals can be solved in R (R: A Language and Environment for Statistical Computing. R Foundation for Statistical ComputingVienna, Austria 2016) by using the "pmvnorm" function in package "mvtnorm" (Genz et al. mvtnorm: Multivariate Normal and t Distributions 2016. R package version 1.0-5).

Note that $E[Z_s] = \sum_{i=1}^{s}(p_i - p_{i-1})E[\bar{X}_{1i}]$. Therefore, we can use the density for $\mathbf{Z}$ to obtain the term $\sum_{i=1}^{s}(p_i - p_{i-1})E[\bar{X}_{1i}\mathbf{1}_{[S_s]}]$ (in expression (2) in main paper). For example, if $s = 1$,

$$\begin{aligned} \sum_{i=1}^{1}(p_i - p_{i-1})E\left[\bar{X}_{1i}\mathbf{1}_{[S_1]}\right] &= p_1 E\left[\bar{X}_{11}\mathbf{1}_{[S_1]}\right] = p_1 E\left[Z_1 \mathbf{1}_{[S_1]}\right] \\ &= p_1 \int_b^\infty \int_{-\infty}^{p_2 b} \int_{-\infty}^{p_3 b} \cdots \int_{-\infty}^{p_{K-1}b} \int_{-\infty}^{b} z_1 f(\mathbf{z}) dz_K dz_{K-1} \cdots dz_3 dz_2 dz_1 \end{aligned}$$

and if $s = 2$,

$$\begin{aligned} \sum_{i=1}^{2}(p_i - p_{i-1})E\left[\bar{X}_{1i}\mathbf{1}_{[S_2]}\right] &= p_1 E\left[\bar{X}_{11}\mathbf{1}_{[S_2]}\right] + (p_2 - p_1)E\left[\bar{X}_{12}\mathbf{1}_{[S_2]}\right] = E\left[Z_2 \mathbf{1}_{[S_2]}\right] \\ &= \int_{-\infty}^\infty \int_{p_2 b}^{\infty} \int_{-\infty}^{p_3 b} \cdots \int_{-\infty}^{p_{K-1}b} \int_{-\infty}^{b} z_2 f(\mathbf{z}) dz_K dz_{K-1} \cdots dz_3 dz_2 dz_1. \end{aligned}$$

Kan and Robotti (http://papers.ssrn.com/sol3/papers.cfm?abstract_id=2748900) give an analytic iterative expression to give such truncated multivariate normal moments (see an example in the next section). Computationally, using such expressions to obtain solutions to the above integrals is very quick compared to using K dimensional numerical integration methods.

4 Solutions for moments

Following Kan and Robotti, we provide a computationally easier to evaluate expression for $E\left[Z_1\mathbf{1}_{[S_1]}\right]$ when $K=4$. Let θ^{Z_i} ($i=1,...,4$) denote the true mean for Z_i. The exact expression for each θ^{Z_i} is given in the main paper (Section 2.3). Let $\mathbf{c}$ denote the vector $(1,0,0,0)'$.

For the variance-covariance matrix for $\mathbf{Z}$ (matrix $\Sigma_{\mathbf{Z}}$), let Σ_{ij} be the ij^{th} entry, $\Sigma_{-i,-i}$ the resulting matrix after excluding the ith row and i^{th} column, $\Sigma_{-i,i}$ the single column matrix that is obtained after excluding the ith entry in column i, $\Sigma_{i,-i}$ the single row matrix that is obtained after excluding the ith entry in row i, and define

$$\tilde{\Sigma}_i = \Sigma_{-i,-i} - \frac{1}{\Sigma_{ii}}\Sigma_{-i,i}\Sigma_{i,-i}.$$

Let $\theta^Z = (\theta^{Z_1}, \theta^{Z_2}, \theta^{Z_3}, \theta^{Z_4})'$ and θ^Z_{-i} denote the vector obtained after excluding the ith entry in θ^Z. Let $\theta^1_{adj} = \frac{b-\theta^{Z_1}}{\Sigma_{ii}}\Sigma_{-1,1}$ and for l ($l=2,3,4$) let $\theta^l_{adj} = \frac{p_l b-\theta^{Z_l}}{\Sigma_{ll}}\Sigma_{-l,l}$ and for i ($i=1,...,4$), define

$$\tilde{\theta}^Z_{-i} = \theta^Z_{-i} + \theta^i_{adj}.$$

Let $\tilde{\mathbf{Z}}^{(i)}$ be the multivariate normal random variable with mean vector $\tilde{\theta}^Z_{-i}$ and variance-covariance matrix $\tilde{\Sigma}_i$. We denote the jth entry of $\tilde{\mathbf{Z}}^{(i)}$ by $\tilde{Z}^{(i)}_j$ and the density for $\tilde{\mathbf{Z}}^{(i)}$ by $f(\tilde{\mathbf{z}}^{(i)})$. Define

$$\tilde{a}_1 = \int_{-\infty}^{p_2 b}\int_{-\infty}^{p_3 b}\int_{-\infty}^{b} f(\tilde{\mathbf{z}}^{(1)})d\tilde{z}^{(1)}_3 d\tilde{z}^{(1)}_2 d\tilde{z}^{(1)}_1, \qquad \tilde{a}_2 = \int_{b}^{\infty}\int_{-\infty}^{p_3 b}\int_{-\infty}^{b} f(\tilde{\mathbf{z}}^{(2)})d\tilde{z}^{(2)}_3 d\tilde{z}^{(2)}_2 d\tilde{z}^{(2)}_1,$$
$$\tilde{a}_3 = \int_{b}^{\infty}\int_{-\infty}^{p_2 b}\int_{-\infty}^{b} f(\tilde{\mathbf{z}}^{(3)})d\tilde{z}^{(3)}_3 d\tilde{z}^{(3)}_2 d\tilde{z}^{(3)}_1 \quad \text{and} \quad \tilde{a}_4 = \int_{b}^{\infty}\int_{-\infty}^{p_2 b}\int_{-\infty}^{p_3 b} f(\tilde{\mathbf{z}}^{(4)})d\tilde{z}^{(4)}_3 d\tilde{z}^{(4)}_2 d\tilde{z}^{(4)}_1.$$

Let $\mathbf{e}$ be the vector whose first entry is $\frac{\tilde{a}_1}{\Sigma_{11}}\phi\left(\frac{b-\theta^{Z_1}}{\Sigma_{11}}\right)$ and the ith entry ($i=2,3,4$) is $\frac{\tilde{a}_i}{\Sigma_{ii}}\phi\left(\frac{p_i b-\theta^{Z_i}}{\Sigma_{ii}}\right)$. Let d_{11} denote the first entry in the matrix $\mathbf{c}^T\Sigma_{\mathbf{Z}}\mathbf{e}$ then

$$E\left[Z_1\mathbf{1}_{[S_1]}\right] = \theta^{Z_1}\text{Prob}(S_1) + d_{11} = \delta_1\text{Prob}(S_1) + d_{11}.$$

Note that the above expression only involves computing multivariate normal probabilities which can be done using "pmvnorm" function in package "mvtnorm" (Genz *et al.* 2016; *mvtnorm: Multivariate Normal and t Distributions.* R package version 1.0-5).

5 Computing the multiple iterations bias adjusted estimator

Here we give details of computing the multiple iterations bias adjusted estimator. Specifically we give the expressions for the bias function. Recall $\hat{\delta}_i$ ($i = 1,...,K$) denotes the naive estimator for δ_i and $\hat{\boldsymbol{\delta}} = (\hat{\delta}_1,...,\hat{\delta}_K)'$. The biases for the naive estimators depend on $\boldsymbol{\delta}$ and we denote bias for $\hat{\delta}_i$ ($i = 1,...,K$) by $b_i(\boldsymbol{\delta})$ and the vector $(b_1(\boldsymbol{\delta}),...,b_K(\boldsymbol{\delta}))$ by $\boldsymbol{b}(\boldsymbol{\delta})$. The second adjusted estimator, which we refer to as multiple iterations bias adjusted estimator is obtained by solving $\tilde{\boldsymbol{\delta}} = \hat{\boldsymbol{\delta}} - \boldsymbol{b}(\tilde{\boldsymbol{\delta}})$ iteratively. The initial value of $\tilde{\boldsymbol{\delta}}$ could be set to be $\hat{\boldsymbol{\delta}}$. In each iteration, an updated estimate $\boldsymbol{b}(\tilde{\boldsymbol{\delta}})$, which involve evaluating expected values $E\left[\bar{X}_{1i}|S_s\right] = E\left[\bar{X}_{1i}\mathbf{1}_{[S_s]}\right]/\text{Prob}(S_s)$ ($i = 1,...,K$), is calculated. Because $E\left[\bar{X}_{11}\mathbf{1}_{[S_s]}\right] = E\left[Z_1\mathbf{1}_{[S_s]}\right]$, $E\left[\bar{X}_{12}\mathbf{1}_{[S_s]}\right] = \left(E\left[Z_2\mathbf{1}_{[S_s]}\right] - p_1 E\left[Z_1\mathbf{1}_{[S_s]}\right]\right)/(p_2 - p_1)$ and for i ($i = 3,...,K$), $E\left[\bar{X}_{1i}\mathbf{1}_{[S_s]}\right] = \left(E\left[Z_i\mathbf{1}_{[S_s]}\right] - E\left[Z_{i-1}\mathbf{1}_{[S_s]}\right]\right)/(p_i - p_{i-1})$, the expectations can be obtained using the density for $\mathbf{Z}$, which is quicker. It can be shown that for i ($i = 1,...,s$), $b_i(\hat{\boldsymbol{\delta}}) = t_s\left(E\left[\bar{X}_{1i}|S_s\right] - \delta_i\right)$ and for i ($i = s+1,...,K$), $b_i(\hat{\boldsymbol{\delta}}) = E\left[\bar{X}_{1i}|S_s\right] - \delta_i$. Suppose that the solution for $\tilde{\boldsymbol{\delta}} = \hat{\boldsymbol{\delta}} - \boldsymbol{b}(\tilde{\boldsymbol{\delta}})$ is achieved at iteration r, the bias for $\hat{\delta}_i$ when $\boldsymbol{\delta}$ is taken to be $\tilde{\boldsymbol{\delta}}_{\boldsymbol{r}}$ is denoted by $b_i(\tilde{\boldsymbol{\delta}}_{\boldsymbol{r}})$.

6 More details for the second shrinkage estimator

As mentioned in Section 3.3.3 in the main paper, the second shrinkage estimator involves using the entire parameter vector $\boldsymbol{\theta}$. Where I_K is a $K \times K$ identity matrix, the prior $MVN(\boldsymbol{\mu}, \nu^2 I_K)$ distribution for $\boldsymbol{\theta}$ is updated with data $\bar{\mathbf{Y}}_1$ whose density is $MVN(\boldsymbol{\theta}, \Sigma_Y)$ (See Section 2.2 in the main paper and Section 1 of this document for Σ_Y). Consequently, the posterior mean for $\boldsymbol{\theta}$ is $C\bar{\mathbf{Y}}_1 + (I_K - C)\mu$, where $C = I_K - \Sigma_Y(\nu^2 I_K + \Sigma)^{-1}$. We replace the unknown μ with $\bar{Y}_{1K}$, the pooled mean from all patients. For C, following Morris (Morris, 1983. Journal of the American Statistical Association ***78***, 47-55) and Brüncker *et al.* (Brüncker *et al.*, 2017. Statistics in Medicine ***36***, 3137-3153), we use an iterative procedure to estimate ν^2.

The marginal variance covariance matrix based on the posterior for $\boldsymbol{\theta}$ is $\nu^2 I_K + \Sigma_Y$. Since Σ_Y is symmetric, using single value decomposition, the diagonal matrix D consisting of eigenvalues for Σ_Y is such that $D = U\Sigma_Y U'$, where matrix U is an orthogonal matrix. Consequently, the marginal covariance based on the posterior for $U\boldsymbol{\theta}$ is $\nu^2 I_K + D$. Since $\nu^2 I_K + D$ is a diagonal matrix, it is computational faster to compute an estimate for ν^2 using the density for $U\boldsymbol{\theta}$ than the density for $\boldsymbol{\theta}$. The iterative procedure is given by Brüncker *et al.* and it is; start with an initial guess for ν^2 $(\hat{\nu}^2)$ and then repeat the following steps until convergence.

- Define weights $w_i = (\hat{\nu}^2 + D_{ii}^2)^{-1}$ $(i = 1, ..., K)$.
- Calculate an updated estimate for ν^2 as $\hat{\nu}^2 = \frac{\sum_{i=1}^{K} w_i[(\bar{y}_{1i} - \bar{y}_{1K}) - D_{ii}^2]}{\sum_{i=1}^{K} w_i}$.

Let $\tilde{\nu}^2$ denote the solution for the iteration procedure, and define $\tilde{\nu}_+^2 = \max\{0, \tilde{\nu}^2\}$ and $\tilde{C}_+ = I_K - \Sigma(\tilde{\nu}_+^2 I_K + \Sigma_Y)^{-1}$. The shrinkage estimator for $\boldsymbol{\theta}$ is

$$\hat{\boldsymbol{\theta}}_{L_2} = \tilde{C}_+ \bar{\mathbf{Y}}_1 + (I_K - \tilde{C}_+)\bar{Y}_{1K},$$

so that the second shrinkage estimator for θ_s that we denote by $\hat{\theta}_{s,L_2}$ is the sth element of $\hat{\boldsymbol{\theta}}_{L_2}$.

7 Worked example in greater detail

7.1 Description of computing estimates

To demonstrate how to compute the various estimates using trial data, we construct an example following Uher *et al.* (2012) (Uher *et al.*, 2012. Pharmacogenomics ***13(2)***, 233-240), who consider trials for depression. One of the outcome measures for trials for depression is the HRSD score. We assume a trial that consists of a prespecified number of weeks of treatment with the outcome measure being the HRSD score at the final follow up visit. The primary analysis is based on the mean difference of the HRSD scores between two interventions. Based on two trials (Rush *et al.*, 2004. Controlled clinical trials ***25(1)***, 119-142; Uher *et al.*, 2009. The British Journal of Psychiatry ***194***, 252-259), a standard deviation of 7 for HRSD scores is reasonable (Uher *et al.*; 2012) and so we take $\sigma = 7$. Uher *et al.* (2012) report that the UK NICE guidelines recommend an intervention for depression should demonstrate a difference of at least 3 HRSD points to be considered superior to its comparator and so at interim we take the futility boundary to be 2 points, that is $b = 2$. Uher *et al.* (2012) give examples of continuous biomarkers in depression such as protein levels in blood and an electrophysiological measure. We assume a case where one of these biomarkers is considered in a trial and quartiles are used to define four partitions so that $K = 4$ with $p_1 = 0.25$, $p_2 = 0.5$, $p_3 = 0.75$ and $p_4 = 1$.

For a single stage pairwise comparison, with $\sigma = 7$, to detect a difference of 3 HRSD scores with 90% power, 115 patients are required in each intervention arm. We aim for a two-stage adaptive threshold enrichment clinical trial that has sufficient power if one partition is selected. Therefore we take the number of patients in each partition in stage 1 to be 90 (45 in each intervention arm), that is $n_{11} = n_{12} = n_{13} = n_{14} = 90$, and take the total stage 2 sample size to be 240. With this sample size, if one partition is selected, the combined stage 1 and 2 sample size for the selected partition is 330, with 165 in each intervention arm. The 50 patients above the 115 patients required in a single stage trial allows for loss of power due to adaptation. Note that $\tau_{11}^2 = ... = \tau_{14}^2 = (4 \times 7^2)/90 = 2.178$. By definition $m_{1l} = \sum_{i=1}^{l} n_{1i}$ so that $m_{11} = 90$, $m_{12} = 180$, $m_{13} = 270$ and $m_{14} = 360$. Consequently, $\sigma_{11}^2 = (4 \times 7^2)/90 = 2.178$, $\sigma_{12}^2 = (4 \times 7^2)/180 = 1.089$, $\sigma_{13}^2 = (4 \times 7^2)/270 = 0.726$ and $\sigma_{14}^2 = (4 \times 7^2)/360 = 0.544$.

Suppose that in stage 1 the observed sample mean differences in partitions 1 to 4 are $\bar{x}_{11} = 3$, $\bar{x}_{12} = 2$, $\bar{x}_{13} = 0.8$ and $\bar{x}_{14} = 0$. Subpopulations 1 to 4 (S_1, S_2, S_3 and S_4) sample mean differences are $\bar{y}_{11} = 3$, $\bar{y}_{12} = 2.5$, $\bar{y}_{13} = 1.93$ and $\bar{y}_{14} = 1.45$. Using the selection rule given in Section 2.2 in the main paper, subpopulation 2 (S_2) would be selected since it is the largest subpopulation with observed mean difference of at least 2 points. The aim of the study would be to estimate θ_2. Since S_2 is selected, the number of patients in each partition in stage 2 is 120. Consequently, $\tau_{21}^2 = \tau_{22}^2 = \sigma_{21}^2 = (4 \times 7^2)/120 = 1.633$ and $\sigma_{22}^2 = (4 \times 7^2)/240 = 0.817$.

Suppose that in stage 2 the observed sample mean differences in partitions 1 and 2 are $\bar{x}_{21} = 3.0$ and $\bar{x}_{22} = 2.4$. Consequently the stage 2 observed sample mean difference for S_2 is $\bar{y}_{22} = 2.7$. The ***naive estimate*** is the two stage sample mean difference for S_2 given by $\hat{\theta}_{2,N} = [(180 \times 2.5) + (240 \times 2.7)]/420 = \mathbf{2.614}$.

For the UMVCUE given by expression (3) in the main paper, since $s = 2$, then the first term is $\hat{\theta}_{2,N} = 2.614$. Further $\sigma_{1s}^2 = \sigma_{12}^2 = 1.089$ and $\sigma_{2s}^2 = \sigma_{22}^2 = 0.817$ so that $\frac{\sigma_{2s}^2}{\sqrt{\sigma_{1s}^2 + \sigma_{2s}^2}} = \frac{\sigma_{22}^2}{\sqrt{\sigma_{12}^2 + \sigma_{22}^2}} = 0.592$ and $\frac{\sqrt{\sigma_{1s}^2 + \sigma_{2s}^2}}{\sigma_{1s}^2} = \frac{\sqrt{\sigma_{12}^2 + \sigma_{22}^2}}{\sigma_{12}^2} = 1.268$. Since $(p_i - p_{i-1}) = 0.25$ for all $i = 1, ..., 4$, then u (the observed

Table 1: Worked example data and estimates

Data and summary measures								Estimating θ_2	
		Stage 1 partitions				Stage 2 partitions			
Measure	Subgroup	1	2	3	4	1	$s=2$	Estimator	Estimate
Sample size	Partition	$n_{11}=90$	$n_{12}=90$	$n_{13}=90$	$n_{14}=90$	$n_{21}=120$	$n_{22}=120$	$\hat{\theta}_{2,N}$	2.614
	Subgroup	$m_{11}=90$	$m_{12}=180$	$m_{13}=270$	$m_{14}=360$	$m_{21}=120$	$m_{22}=240$	$\hat{\theta}_{2,UMV}$	2.839
Sample variance	Partition	$\tau^2_{11}=2.178$	$\tau^2_{12}=2.178$	$\tau^2_{13}=2.178$	$\tau^2_{14}=2.178$	$\tau^2_{21}=1.633$	$\tau^2_{22}=1.633$	$\hat{\theta}_{2,U}$	2.965
	Subgroup	$\sigma^2_{11}=2.178$	$\sigma^2_{12}=1.089$	$\sigma^2_{13}=0.726$	$\sigma^2_{14}=0.544$	$\sigma^2_{21}=1.633$	$\sigma^2_{22}=0.817$	$\hat{\theta}_{2,SI}$	2.633
Sample mean	Partition	$\bar{x}_{11}=3$	$\bar{x}_{12}=2$	$\bar{x}_{13}=0.8$	$\bar{x}_{14}=0$	$\bar{x}_{21}=3$	$\bar{x}_{22}=2.4$	$\hat{\theta}_{2,MI}$	2.666
	Subgroup	$\bar{y}_{11}=3$	$\bar{y}_{12}=2.5$	$\bar{y}_{13}=1.93$	$\bar{y}_{14}=1.45$	$\bar{y}_{21}=3$	$\bar{y}_{22}=2.7$	$\hat{\theta}_{2,L_1}$	2.164
								$\hat{\theta}_{2,L_2}$	2.194

value for U in the expression for the UMVCUE) is given by

$$\begin{aligned} u &= \min\left\{\frac{p_3 b-(p_3-p_2)\bar{X}_{13}}{p_2}, \frac{p_4 b-\sum_{i=3}^{4}(p_i-p_{i-1})\bar{X}_{1i}}{p_2}\right\} \\ &= \min\left\{\frac{(0.75\times 2)-(0.25\times 0.8)}{0.5}, \frac{2-[0.25\times(0.8+0)]}{0.5}\right\} = \min\{2.6, 3.6\} = 2.6. \end{aligned}$$

Note that $f(b) = 1.268\times(2.614-2) = 0.779$ and $f(u) = 1.268\times(2.614-2.6) = 0.018$. Now we have all components to substitute in the expression for the UMVCUE so that the estimate corresponding to ***UMVCUE*** is $\hat{\theta}_{2,UMV} = \mathbf{2.839}$.

Next we compute the estimate corresponding to the unbiased estimator given by expression (4) in the main paper. The naive estimates for partitions 1 and 2 are $\hat{\delta}_{1,N} = [(90\times 3)+(120\times 3)]/210 = 3$ and $\hat{\delta}_{2,N} = [(90\times 2)+(120\times 2.4)]/210 = 2.229$, respectively. Note that $\frac{\tau_{21}^2}{\sqrt{\tau_{11}^2+\tau_{21}^2}} = \frac{\tau_{22}^2}{\sqrt{\tau_{21}^2+\tau_{22}^2}} = 0.837$ and $\frac{\sqrt{\tau_{11}^2+\tau_{21}^2}}{\tau_{11}^2} = \frac{\sqrt{\tau_{21}^2+\tau_{22}^2}}{\tau_{21}^2} = 0.896$. Since $p_i - p_{i-1} = 0.25$ $(i = 1,\ldots,4)$, $v_1 = \frac{1}{0.25}\left(p_2 b - 0.25\sum_{\substack{i=1\\i\neq 1}}^{2}\bar{x}_{1i}\right)$ $= 4\times[(0.5\times 0.2)-(0.25\times 2)] = 2$ and $v_2 = \frac{1}{0.25}\left(p_2 b - 0.25\sum_{\substack{i=1\\i\neq 2}}^{2}\bar{x}_{1i}\right) = 4\times[(0.5\times 0.2)-(0.25\times 3)] = 1$, respectively. For partition 1,

$$\begin{aligned} w_1 &= \min\left\{\frac{p_3 b - 0.25\sum_{\substack{i=1\\i\neq 1}}^{3}\bar{x}_{1i}}{0.25}, \frac{p_4 b - 0.25\sum_{\substack{i=1\\i\neq 1}}^{4}\bar{x}_{1i}}{0.25}\right\} \\ &= \min\left\{\frac{(0.75\times 2)-0.25\times(2+0.8)}{0.25}, \frac{2-0.25\times(2+0.8+0)}{0.25}\right\} = 3.2. \end{aligned}$$

Similarly, for partition 2, $w_2 = 2.2$. Then for partition 1, $f(v_1) = 0.896\times(3-2) = 0.896$ and $f(w_1) = 0.896\times(3-3.2) = -0.179$ and for partition 2, $f(w_2) = 0.896\times(2.229-1) = 1.101$ and $f(w_2) = 0.896\times(2.229-2.2) = 0.026$. Now we have all components required to obtain UMVCUEs for effects in partitions 1 and 2, which give $\hat{\delta}_{1,UMVCUE} = 3.272$ and $\hat{\delta}_{2,UMVCUE} = 2.657$, respectively. The ***unbiased estimate*** corresponding to expression (4) in the main paper is the weighted UMVCUEs in the partitions giving $\hat{\theta}_{2,U} = \mathbf{2.965}$.

For the single iteration bias adjusted estimator, we first note that the naive estimates for partitions 1 to 4 are 3, 2.229, 0.8 and 0, respectively. These are taken to be the true values for δ_1, δ_2, δ_3 and δ_4 while computing bias. Also, the naive estimate for θ_2, ($\hat{\theta}_{2,N} = 2.614$) is taken as the true value while computing bias. Following expression (2) in the main paper, since $t_s = t_2 = m_{12}/(m_{12}+m_{22}) = 180/420 = 0.429$ and $p_i - p_{i-1} = 0.25$ $(i = 1,\ldots,4)$, bias is given by

$$0.429\left\{\frac{0.25\,(E\,[\bar{X}_{11}\mathbf{1}_{S_2}] + E\,[\bar{X}_{12}\mathbf{1}_{S_2}])}{0.5\times \text{Prob}(S_2)} - \hat{\theta}_{2,N}\right\}.$$

As described in Section 2.3 in the main paper and Section 3 in this document, to compute $0.25\,(E\,[\bar{X}_{11}\mathbf{1}_{S_2}] + E\,[\bar{X}_{12}\mathbf{1}_{S_2}])$ and Prob(S_2), we use a new vector $\mathbf{Z}$. In this example, the components of $\mathbf{Z}$ are $\bar{X}_{11}$, $0.25\sum_{i=1}^{2}\bar{X}_{1i}$, $0.25\sum_{i=1}^{3}\bar{X}_{1i}$ and $0.25\sum_{i=1}^{4}\bar{X}_{1i}$. Using the naive estimates, the components for the mean vector for $\mathbf{Z}$ are 3, $0.25\times(3+2.229) = 1.307$, $0.25\times(3+2.229+0.8) = 1.507$ and $0.25\times(3+2.229+0.8+0) =$

1.507. Since $p_i - p_{i-1} = 0.25$ ($i = 1,...,4$) and $\tau_{1i}^2 = 2.178$ ($i = 1,...,4$), the column entries for the variance covariance matrix for $\boldsymbol{Z}$ is

$$\Sigma_Z = \begin{pmatrix} 2.178 & 0.545 & 0.545 & 0.545 \\ 0.545 & 0.272 & 0.272 & 0.272 \\ 0.545 & 0.272 & 0.408 & 0.408 \\ 0.545 & 0.272 & 0.408 & 0.545 \end{pmatrix}.$$

To show the limits of integration, for example,

$$\text{Prob}(S_2) = \int_{-\infty}^{\infty}\int_{p_2 b}^{\infty}\int_{-\infty}^{p_3 b}\int_{-\infty}^{b} f(z)dz_4dz_3dz_2dz_1 = \int_{-\infty}^{\infty}\int_{1}^{\infty}\int_{-\infty}^{1.5}\int_{-\infty}^{2} f(z)dz_4dz_3dz_2dz_1,$$

which using function "pmvnorm" in package "mvtnorm" gives $\text{Prob}(S_2) = 0.232$. As described in Section 3, $0.25\left(E\left[\bar{X}_{11}\mathbf{1}_{S_2}\right] + E\left[\bar{X}_{12}\mathbf{1}_{S_2}\right]\right) = E\left[Z_2\mathbf{1}_{S_2}\right]$ and is the above expression after inserting z_2 infront of $f(z)$. Following Kan and Robotti, we have written a program in R to compute $E\left[Z_2\mathbf{1}_{S_2}\right]$ with the solution being 0.298 (see next section). Thus the estimated bias is $0.429\left\{\frac{0.298}{0.5\times0.232} - 2.614\right\} = -0.019$ so that the ***single iteration bias adjusted estimate*** is 2.613+0.019 = **2.633**.

For the multiple iterations adjusted estimate we solved $\tilde{\delta} = \hat{\delta} - b(\tilde{\delta})$. From Section 3.3 in the main paper, the ith ($i = 1,2$) component in the bias function is $b_i(\hat{\delta}) = t_2\left(E\left[\bar{X}_{1i}|S_2\right] - \delta_i\right)$, the ith ($i = 3,4$) component in the bias function is $b_i(\hat{\delta}) = E\left[\bar{X}_{1i}|S_2\right] - \delta_i$ and for i ($i = 1,...,4$), $E\left[\bar{X}_{1i}|S_2\right] = E\left[\bar{X}_{1i}\mathbf{1}_{S_2}\right]/\text{Prob}(S_2)$. Further $E\left[\bar{X}_{11}\mathbf{1}_{S_2}\right] = E\left[Z_1\mathbf{1}_{S_2}\right]$, $E\left[\bar{X}_{12}\mathbf{1}_{S_2}\right] = \left(E\left[Z_2\mathbf{1}_{S_2}\right] - 0.25E\left[Z_1\mathbf{1}_{S_2}\right]\right)/0.25$, $E\left[\bar{X}_{13}\mathbf{1}_{S_2}\right] = \left(E\left[Z_3\mathbf{1}_{S_2}\right] - E\left[Z_2\mathbf{1}_{S_2}\right]\right)/0.25$ and $E\left[\bar{X}_{14}\mathbf{1}_{S_2}\right] = \left(E\left[Z_4\mathbf{1}_{S_2}\right] - E\left[Z_3\mathbf{1}_{S_2}\right]\right)/0.25$. We have written a program in R to perform these calculations. Using the program the ***multiple iterations bias adjusted estimate*** is $\hat{\theta}_{2,MI} = \mathbf{2.666}$.

For the first shrinkage estimator, we note that $\hat{C} = 1 - 2(K-3)\sigma^2/[n\sum_{j=1}^{K}(\bar{x}_{1j} - \bar{y}_{1K})^2 = 1 - 2\sigma^2/[n\sum_{j=1}^{4}(\bar{x}_{1j} - \bar{y}_{14})^2]$. Here n is the number of patients in each arm in a partition and so is equal to 45. Substituting values, $\hat{C} = -2.373$ so that $\hat{C}_+ = \max\{0, \hat{C}\} = 0$. Hence, the shrinkage estimates for partitions 1 and 2 are $\hat{\delta}_{1,L} = t_2[\hat{C}_+\bar{x}_{11} + (1-\hat{C}_+)\bar{y}_{14}] + (1-t_2)\bar{x}_{21} = 2.335$ and $\hat{\delta}_{2,L} = t_2[\hat{C}_+\bar{x}_{12} + (1-\hat{C}_+)\bar{y}_{14}] + (1-t_2)\bar{x}_{22} = 1.992$ so that the ***first shrinkage estimate*** given by $(0.25\hat{\delta}_{1,L} + 0.25\hat{\delta}_{2,L})/0.5$ is $\hat{\theta}_{2,L_1} = \mathbf{2.164}$.

For the second shrinkage estimator, the variance covariance matrix for Y is

$$\Sigma_Y = \begin{pmatrix} 2.178 & 1.089 & 0.726 & 0.545 \\ 1.089 & 1.089 & 0.726 & 0.545 \\ 0.726 & 0.726 & 0.726 & 0.545 \\ 0.545 & 0.545 & 0.545 & 0.545 \end{pmatrix}.$$

The algorithm for obtaining the second shrinkage estimator is given Section 3.4 in the main paper and we wrote an R programme to implement it. Using the program the ***second shrinkage estimate*** is $\hat{\theta}_{2,L_2} = \mathbf{2.194}$.

The unbiased estimates ($\hat{\theta}_{2,UMV}$ and $\hat{\theta}_{2,U}$) and the bias adjusted estimates ($\hat{\theta}_{2,SI}$ and $\hat{\theta}_{2,MI}$) are greater than the naive estimate. This may be explained by the observation in Section 5.2 in the main paper that in some scenarios the naive estimator is negatively biased. The single iteration bias adjusted estimate ($\hat{\theta}_{2,SI}$) is slightly smaller than the multiple iterations bias adjusted estimate ($\hat{\theta}_{2,MI}$). This may be explained by an observation in Section 5.2 in the main paper that for all scenarios in the simulation study, on average the single iterations bias adjusted estimator gives a smaller estimate than the multiple iterations estimator.

7.2 R code used to compute estimates

7.2.1 Loading the required package

```
library(mvtnorm)
```

7.2.2 Naive estimate

```
naive.estimate <- round(((180*2.5)+(240*2.7))/420,3)
naive.estimate
```

7.2.3 UMVCUE estimate

Futility boundary

```
b <- 2

sigma.12 <- round(4*7*7/180, 3)
sigma.22 <- round(4*7*7/240, 3)

scaling1 <- round(sigma.22/sqrt(sigma.12+sigma.22), 3)
scaling2 <- round(sqrt(sigma.12+sigma.22)/sigma.12, 3)

u <- min(((0.75*2) - (0.25*0.8))/0.5, (2 - (0.25*(0.8+0)))/0.5)

f.b <- round(scaling2*(naive.estimate - b), 3)
f.u <- round(scaling2*(naive.estimate - u), 3)

UMVCUE.estimate <- round(naive.estimate - (scaling1 * ((dnorm(f.b)-dnorm(f.u))/(pnorm(f.b)-pnorm(f.u)))), 3)
UMVCUE.estimate
```

7.2.4 Unbiased estimate

Partition 1 UMVCUE for δ_1

```
b <- 2

naive.estimate1 <- round(((90*3)+(120*3))/210,3)
tau.11 <- round(4*7*7/90, 3)
tau.21 <- round(4*7*7/120, 3)

scaling1 <- round(tau.21/sqrt(tau.11+tau.21), 3)
scaling2 <- round(sqrt(tau.11+tau.21)/tau.11, 3)

v.1 <- 4 * ( (0.5*b) - (0.25*2) )
w.1 <- min(c(((0.75*b) - (0.25 * (2+0.8)))/0.25, (b - (0.25 * (2+0.8+0)))/0.25))
f.v1 <- round(scaling2*(naive.estimate1 - v.1), 3)
f.w1 <- round(scaling2*(naive.estimate1 - w.1), 3)

delta1.estimate <- round(naive.estimate1 - (scaling1 * ((dnorm(f.v1)-dnorm(f.w1))/(pnorm(f.v1)-pnorm(f.w1)))), 3)
delta1.estimate
```

Partition 2 UMVCUE for δ_2

```
b <- 2

naive.estimate2 <- round(((90*2)+(120*2.4))/210,3)
tau.12 <- round(4*7*7/90, 3)
tau.22 <- round(4*7*7/120, 3)

scaling1 <- round(tau.22/sqrt(tau.12+tau.22), 3)
scaling2 <- round(sqrt(tau.12+tau.22)/tau.12, 3)

v.2 <- 4 * ( (0.5*b) - (0.25*3) )
w.2 <- min(c(((0.75*b) - (0.25 * (3+0.8)))/0.25, (b - (0.25 * (3+0.8+0)))/0.25))
f.v2 <- round(scaling2*(naive.estimate2 - v.2), 3)
f.w2 <- round(scaling2*(naive.estimate2 - w.2), 3)

delta2.estimate <- round(naive.estimate2 - (scaling1 * ((dnorm(f.v2)-dnorm(f.w2))/(pnorm(f.v2)-pnorm(f.w2)))), 3)
delta2.estimate
```

Unbiased estimate for θ_2

```
unbiased.estimate <- round(((0.25 * delta1.estimate) + (0.25 * delta2.estimate))/(0.25+0.25), 3)
unbiased.estimate
```

7.2.5 Functions to obtain expected values that are required while computing single iteration and multiple iterations bias adjusted estimates

Function to compute $E[Z_2 1_{S_2}]$

```
expectedsel2 <- function(zmeans, zmatrix1, b1, prevVector, ProbSel1){
    z1mean <- zmeans[1]
    z2mean <- zmeans[2]
    z3mean <- zmeans[3]
    z4mean <- zmeans[4]

    zmatrix <- zmatrix1

    b <- b1

    p2 <- prevVector[2]
    p3 <- prevVector[3]

    ProbSel <- ProbSel1

    a1 <- z2mean*ProbSel

    vector1 <- matrix(c(0, 1, 0, 0), nrow=1)
    vector2 <- rep(99, 4)

    vector2[1] <- 0

    zmatrix_tilde2 <- zmatrix[-2,-2]
    test <- matrix(zmatrix[-2,2], ncol=1)
    test1 <- matrix(zmatrix[2,-2], nrow=1)
    zmatrix_tilde2_adj <- (1/zmatrix[2,2]) * (test %*% test1)
    zmatrix_tilde2 <- zmatrix_tilde2 - zmatrix_tilde2_adj
    mu2_tilde <- c(z1mean, z3mean, z4mean)
    mu2_tilde_adj <- (((p2*b)-z2mean)/zmatrix[2,2]) * zmatrix[-2,2]
    mu2_tilde <- mu2_tilde + mu2_tilde_adj
    cc1 <- pmvnorm(lower=c(-Inf, -Inf, -Inf), upper=c(Inf, p3*b, b), mean=mu2_tilde, sigma=zmatrix_tilde2, algorithm = GenzBretz())
    cc1 <- cc1[1]
    vector2[2] <- (1/sqrt(zmatrix[2,2])) * dnorm(((p2*b)-z2mean)/sqrt(zmatrix[2,2])) * cc1

    zmatrix_tilde3 <- zmatrix[-3,-3]
    test <- matrix(zmatrix[-3,3], ncol=1)
    test1 <- matrix(zmatrix[3,-3], nrow=1)
    zmatrix_tilde3_adj <- (1/zmatrix[3,3]) * (test %*% test1)
    zmatrix_tilde3 <- zmatrix_tilde3 - zmatrix_tilde3_adj
    mu3_tilde <- c(z1mean, z2mean, z4mean)
    mu3_tilde_adj <- (((p3*b)-z3mean)/zmatrix[3,3]) * zmatrix[-3,3]
    mu3_tilde <- mu3_tilde + mu3_tilde_adj
    cc1 <- pmvnorm(lower=c(-Inf, p2*b, -Inf), upper=c(Inf, Inf, b), mean=mu3_tilde, sigma=zmatrix_tilde3, algorithm = GenzBretz())
    cc1 <- cc1[1]
    vector2[3] <- (-1/sqrt(zmatrix[3,3])) * dnorm(((p3*b)-z3mean)/sqrt(zmatrix[3,3])) * cc1

    zmatrix_tilde4 <- zmatrix[-4,-4]
    test <- matrix(zmatrix[-4,4], ncol=1)
    test1 <- matrix(zmatrix[4,-4], nrow=1)
    zmatrix_tilde4_adj <- (1/zmatrix[4,4]) * (test %*% test1)
    zmatrix_tilde4 <- zmatrix_tilde4 - zmatrix_tilde4_adj
    mu4_tilde <- c(z1mean, z2mean, z3mean)
    mu4_tilde_adj <- ((b-z4mean)/zmatrix[4,4]) * zmatrix[-4,4]
    mu4_tilde <- mu4_tilde + mu4_tilde_adj
    cc1 <- pmvnorm(lower=c(-Inf, p2*b, -Inf), upper=c(Inf, Inf, p3*b), mean=mu4_tilde, sigma=zmatrix_tilde4, algorithm = GenzBretz())
    cc1 <- cc1[1]
    vector2[4] <- (-1/sqrt(zmatrix[4,4])) * dnorm((b-z4mean)/sqrt(zmatrix[4,4])) * cc1

    vector2 <- matrix(vector2, ncol=1)
```

```
    secondterm <- vector1 %*% zmatrix %*% vector2
    a1 <- a1 + secondterm[1,1]

    a1
}
```

Function to compute $E[Z_1 1_{S_2}]$

```
ExpectedSel2_Z1 <- function(zmeans, zmatrix1, b1, prevVector, ProbSel1){
    z1mean <- zmeans[1]
    z2mean <- zmeans[2]
    z3mean <- zmeans[3]
    z4mean <- zmeans[4]

    zmatrix <- zmatrix1

    b <- b1

    p2 <- prevVector[2]
    p3 <- prevVector[3]

    ProbSel <- ProbSel1

    a1 <- z1mean*ProbSel

    vector1 <- matrix(c(1, 0, 0, 0), nrow=1)
    vector2 <- rep(99, 4)

    vector2[1] <- 0

    zmatrix_tilde2 <- zmatrix[-2,-2]
    test <- matrix(zmatrix[-2,2], ncol=1)
    test1 <- matrix(zmatrix[2,-2], nrow=1)
    zmatrix_tilde2_adj <- (1/zmatrix[2,2]) * (test %*% test1)
    zmatrix_tilde2 <- zmatrix_tilde2 - zmatrix_tilde2_adj
    mu2_tilde <- c(z1mean, z3mean, z4mean)
    mu2_tilde_adj <- (((p2*b)-z2mean)/zmatrix[2,2]) * zmatrix[-2,2]
    mu2_tilde <- mu2_tilde + mu2_tilde_adj
    cc1 <- pmvnorm(lower=c(-Inf, -Inf, -Inf), upper=c(Inf, p3*b, b), mean=mu2_tilde, sigma=zmatrix_tilde2, algorithm = GenzBretz())
    cc1 <- cc1[1]
    vector2[2] <- (1/sqrt(zmatrix[2,2])) * dnorm(((p2*b)-z2mean)/sqrt(zmatrix[2,2])) * cc1

    zmatrix_tilde3 <- zmatrix[-3,-3]
    test <- matrix(zmatrix[-3,3], ncol=1)
    test1 <- matrix(zmatrix[3,-3], nrow=1)
    zmatrix_tilde3_adj <- (1/zmatrix[3,3]) * (test %*% test1)
    zmatrix_tilde3 <- zmatrix_tilde3 - zmatrix_tilde3_adj
    mu3_tilde <- c(z1mean, z2mean, z4mean)
    mu3_tilde_adj <- (((p3*b)-z3mean)/zmatrix[3,3]) * zmatrix[-3,3]
    mu3_tilde <- mu3_tilde + mu3_tilde_adj
    cc1 <- pmvnorm(lower=c(-Inf, p2*b, -Inf), upper=c(Inf, Inf, b), mean=mu3_tilde, sigma=zmatrix_tilde3, algorithm = GenzBretz())
    cc1 <- cc1[1]
    vector2[3] <- (-1/sqrt(zmatrix[3,3])) * dnorm(((p3*b)-z3mean)/sqrt(zmatrix[3,3])) * cc1

    zmatrix_tilde4 <- zmatrix[-4,-4]
    test <- matrix(zmatrix[-4,4], ncol=1)
    test1 <- matrix(zmatrix[4,-4], nrow=1)
    zmatrix_tilde4_adj <- (1/zmatrix[4,4]) * (test %*% test1)
    zmatrix_tilde4 <- zmatrix_tilde4 - zmatrix_tilde4_adj
    mu4_tilde <- c(z1mean, z2mean, z3mean)
    mu4_tilde_adj <- ((b-z4mean)/zmatrix[4,4]) * zmatrix[-4,4]
    mu4_tilde <- mu4_tilde + mu4_tilde_adj
    cc1 <- pmvnorm(lower=c(-Inf, p2*b, -Inf), upper=c(Inf, Inf, p3*b), mean=mu4_tilde, sigma=zmatrix_tilde4, algorithm = GenzBretz())
    cc1 <- cc1[1]
    vector2[4] <- (-1/sqrt(zmatrix[4,4])) * dnorm((b-z4mean)/sqrt(zmatrix[4,4])) * cc1

    vector2 <- matrix(vector2, ncol=1)

    secondterm <- vector1 %*% zmatrix %*% vector2
    a1 <- a1 + secondterm[1,1]
```

```
    a1
}
```

Function to compute $EZ_3 1_{S_2}]$

```
ExpectedSel2_Z3 <- function(zmeans, zmatrix1, b1, prevVector, ProbSel1){
    z1mean <- zmeans[1]
    z2mean <- zmeans[2]
    z3mean <- zmeans[3]
    z4mean <- zmeans[4]

    zmatrix = zmatrix1

    b <- b1

    p2 <- prevVector[2]
    p3 <- prevVector[3]

    ProbSel <- ProbSel1

    a1 <- z3mean*ProbSel

    vector1 <- matrix(c(0, 0, 1, 0), nrow=1)
    vector2 <- rep(99, 4)

    vector2[1] <- 0

    zmatrix_tilde2 <- zmatrix[-2,-2]
    test <- matrix(zmatrix[-2,2], ncol=1)
    test1 <- matrix(zmatrix[2,-2], nrow=1)
    zmatrix_tilde2_adj <- (1/zmatrix[2,2]) * (test %*% test1)
    zmatrix_tilde2 <- zmatrix_tilde2 - zmatrix_tilde2_adj
    mu2_tilde <- c(z1mean, z3mean, z4mean)
    mu2_tilde_adj <- (((p2*b)-z2mean)/zmatrix[2,2]) * zmatrix[-2,2]
    mu2_tilde <- mu2_tilde + mu2_tilde_adj
    cc1 <- pmvnorm(lower=c(-Inf, -Inf, -Inf), upper=c(Inf, p3*b, b), mean=mu2_tilde, sigma=zmatrix_tilde2, algorithm = GenzBretz())
    cc1 <- cc1[1]
    vector2[2] <- (1/sqrt(zmatrix[2,2])) * dnorm(((p2*b)-z2mean)/sqrt(zmatrix[2,2])) * cc1

    zmatrix_tilde3 <- zmatrix[-3,-3]
    test <- matrix(zmatrix[-3,3], ncol=1)
    test1 <- matrix(zmatrix[3,-3], nrow=1)
    zmatrix_tilde3_adj <- (1/zmatrix[3,3]) * (test %*% test1)
    zmatrix_tilde3 <- zmatrix_tilde3 - zmatrix_tilde3_adj
    mu3_tilde <- c(z1mean, z2mean, z4mean)
    mu3_tilde_adj <- (((p3*b)-z3mean)/zmatrix[3,3]) * zmatrix[-3,3]
    mu3_tilde <- mu3_tilde + mu3_tilde_adj
    cc1 <- pmvnorm(lower=c(-Inf, p2*b, -Inf), upper=c(Inf, Inf, b), mean=mu3_tilde, sigma=zmatrix_tilde3, algorithm = GenzBretz())
    cc1 <- cc1[1]
    vector2[3] <- (-1/sqrt(zmatrix[3,3])) * dnorm(((p3*b)-z3mean)/sqrt(zmatrix[3,3])) * cc1

    zmatrix_tilde4 <- zmatrix[-4,-4]
    test <- matrix(zmatrix[-4,4], ncol=1)
    test1 <- matrix(zmatrix[4,-4], nrow=1)
    zmatrix_tilde4_adj <- (1/zmatrix[4,4]) * (test %*% test1)
    zmatrix_tilde4 <- zmatrix_tilde4 - zmatrix_tilde4_adj
    mu4_tilde <- c(z1mean, z2mean, z3mean)
    mu4_tilde_adj <- ((b-z4mean)/zmatrix[4,4]) * zmatrix[-4,4]
    mu4_tilde <- mu4_tilde + mu4_tilde_adj
    cc1 <- pmvnorm(lower=c(-Inf, p2*b, -Inf), upper=c(Inf, Inf, p3*b), mean=mu4_tilde, sigma=zmatrix_tilde4, algorithm = GenzBretz())
    cc1 <- cc1[1]
    vector2[4] <- (-1/sqrt(zmatrix[4,4])) * dnorm((b-z4mean)/sqrt(zmatrix[4,4])) * cc1

    vector2 <- matrix(vector2, ncol=1)

    secondterm <- vector1 %*% zmatrix %*% vector2
    a1 <- a1 + secondterm[1,1]

    a1
}
```

Function to compute $EZ_4 1_{S_2}]$

```
ExpectedSel2_Z4 <- function(zmeans, zmatrix1, b1, prevVector, ProbSel1){
    z1mean <- zmeans[1]
    z2mean <- zmeans[2]
    z3mean <- zmeans[3]
    z4mean <- zmeans[4]

    zmatrix <- zmatrix1

    b <- b1

    p2 <- prevVector[2]
    p3 <- prevVector[3]

    ProbSel <- ProbSel1

    a1 <- z4mean*ProbSel

    vector1 <- matrix(c(0, 0, 0, 1), nrow=1)
    vector2 <- rep(99, 4)


    vector2[1] <- 0

    zmatrix_tilde2 <- zmatrix[-2,-2]
    test <- matrix(zmatrix[-2,2], ncol=1)
    test1 <- matrix(zmatrix[2,-2], nrow=1)
    zmatrix_tilde2_adj <- (1/zmatrix[2,2]) * (test %*% test1)
    zmatrix_tilde2 <- zmatrix_tilde2 - zmatrix_tilde2_adj
    mu2_tilde <- c(z1mean, z3mean, z4mean)
    mu2_tilde_adj <- (((p2*b)-z2mean)/zmatrix[2,2]) * zmatrix[-2,2]
    mu2_tilde <- mu2_tilde + mu2_tilde_adj
    cc1 <- pmvnorm(lower=c(-Inf, -Inf, -Inf), upper=c(Inf, p3*b, b), mean=mu2_tilde, sigma=zmatrix_tilde2, algorithm = GenzBretz())
    cc1 <- cc1[1]
    vector2[2] <- (1/sqrt(zmatrix[2,2])) * dnorm(((p2*b)-z2mean)/sqrt(zmatrix[2,2])) * cc1

    zmatrix_tilde3 <- zmatrix[-3,-3]
    test <- matrix(zmatrix[-3,3], ncol=1)
    test1 <- matrix(zmatrix[3,-3], nrow=1)
    zmatrix_tilde3_adj <- (1/zmatrix[3,3]) * (test %*% test1)
    zmatrix_tilde3 <- zmatrix_tilde3 - zmatrix_tilde3_adj
    mu3_tilde <- c(z1mean, z2mean, z4mean)
    mu3_tilde_adj <- (((p3*b)-z3mean)/zmatrix[3,3]) * zmatrix[-3,3]
    mu3_tilde <- mu3_tilde + mu3_tilde_adj
    cc1 <- pmvnorm(lower=c(-Inf, p2*b, -Inf), upper=c(Inf, Inf, b), mean=mu3_tilde, sigma=zmatrix_tilde3, algorithm = GenzBretz())
    cc1 <- cc1[1]
    vector2[3] <- (-1/sqrt(zmatrix[3,3])) * dnorm(((p3*b)-z3mean)/sqrt(zmatrix[3,3])) * cc1

    zmatrix_tilde4 <- zmatrix[-4,-4]
    test <- matrix(zmatrix[-4,4], ncol=1)
    test1 <- matrix(zmatrix[4,-4], nrow=1)
    zmatrix_tilde4_adj <- (1/zmatrix[4,4]) * (test %*% test1)
    zmatrix_tilde4 <- zmatrix_tilde4 - zmatrix_tilde4_adj
    mu4_tilde <- c(z1mean, z2mean, z3mean)
    mu4_tilde_adj <- ((b-z4mean)/zmatrix[4,4]) * zmatrix[-4,4]
    mu4_tilde <- mu4_tilde + mu4_tilde_adj
    cc1 <- pmvnorm(lower=c(-Inf, p2*b, -Inf), upper=c(Inf, Inf, p3*b), mean=mu4_tilde, sigma=zmatrix_tilde4, algorithm = GenzBretz())
    cc1 <- cc1[1]
    vector2[4] <- (-1/sqrt(zmatrix[4,4])) * dnorm((b-z4mean)/sqrt(zmatrix[4,4])) * cc1

    vector2 <- matrix(vector2, ncol=1)

    secondterm <- vector1 %*% zmatrix %*% vector2
    a1 <- a1 + secondterm[1,1]

    a1
}
```

7.2.6 Single iteration bias adjusted estimate

Prorportion of stage 1 data for S_2

```
t <- round(180/(180+240), 3)
```

Prevalence of partitions and subpopulations

```
prop <- c(0.25, 0.25, 0.25, 0.25)

p1 <- prop[1]
p2 <- sum(prop[1:2])
p3 <- sum(prop[1:3])
p4 <- sum(prop[1:4])
```

Futility boundary

```
b <- 2
```

Variance covariance matrix for Z

```
zmatrix <- matrix(NA, ncol=4, nrow=4)
zmatrix[,1] <- c(2.178, rep(0.545,3))
zmatrix[,2] <- c(0.545, rep(0.272, 3))
zmatrix[,3] <- c(0.545, 0.272, rep(0.408, 2))
zmatrix[,4] <- c(0.545, 0.272, 0.408, 0.545)
```

Mean vector for Z

```
z1mean <- 3
z2mean <- 2.229
z3mean <- 0.8
z4mean <- 0
mu <- c(z1mean, z2mean, z3mean, z4mean) z2mean <- (prop[1] * mu[1]) + (prop[2] * mu[2])

z3mean <- (prop[1] * mu[1]) + (prop[2] * mu[2]) + (prop[3] * mu[3])
z4mean <- (prop[1] * mu[1]) + (prop[2] * mu[2]) + (prop[3] * mu[3]) + (prop[4] * mu[4])
```

Probability for selecting S_2

```
ProbSel <- pmvnorm(lower=c(-Inf, p2*b, -Inf, -Inf), upper=c(Inf, Inf, p3*b, b), mean=c(z1mean, z2mean, z3mean, z4mean), sigma=zmatrix,
          algorithm = GenzBretz())
ProbSel <- round(ProbSel[1], 3)
```

Evaluating $EZ_2 1_{S_2}]$

```
ExpSel <- expectedsel2(zmeans=c(z1mean, z2mean, z3mean, z4mean), zmatrix1=zmatrix, b1=b, prevVector=c(p1, p2, p3, p4), ProbSel1=ProbSel)
ExpSel <- round(ExpSel, 3)
ExpSel
```

Calculating bias

```
ExpSel <- (ExpSel)/p2
TwoSubsBias1 <- round(t * ((ExpSel/ProbSel) - 2.614), 3)
TwoSubsBias1
```

Calculating bias adjusted estimate

```
BiasAdjustedEstimate <- 2.614 - TwoSubsBias1
BiasAdjustedEstimate
```

7.2.7 Additional function required to compute multiple iterations bias adjusted estimate

```
twosubs.selected <- function(zmatrix2, NaiveEstimates, b2, prob2, prevVector1, r){
    y <- c(NaiveEstimates[1], NaiveEstimates[2], NaiveEstimates[3], NaiveEstimates[4])
    zrminus <- c(NaiveEstimates[1], NaiveEstimates[2], NaiveEstimates[3], NaiveEstimates[4])

     prop <- prob2

     p2 <- prevVector1[2]
    p3 <- prevVector1[3]

    for (k in 1:200){
          zr <- rep(NA, 4)
```

```
        z1mean <- zrminus[1]
        z2mean <- (prop[4] * zrminus[1]) + (prop[3] * zrminus[2])
        z3mean <- (prop[4] * zrminus[1]) + (prop[3] * zrminus[2]) + (prop[2] * zrminus[3])
        z4mean <- (prop[4] * zrminus[1]) + (prop[3] * zrminus[2]) + (prop[2] * zrminus[3]) + (prop[1] * zrminus[4])

        ProbSel <- pmvnorm(lower=c(-Inf, p2*b2, -Inf, -Inf), upper=c(Inf, Inf, p3*b2, b2), mean=c(z1mean, z2mean, z3mean, z4mean), sigma=zmatrix2,
            algorithm = GenzBretz())
        ProbSel <- ProbSel[1]

        ExpSel <- expectedsel2(zmeans=c(z1mean, z2mean, z3mean, z4mean), zmatrix1=zmatrix2, b1=b2, prevVector=prevVector1,
            ProbSel1=ProbSel)
        ExpSel2_Z2 <- ExpSel/ProbSel
        ExpSel <- (ExpSel)/(prop[3] + prop[4])

        ExpSel2_Z1 <- (ExpectedSel2_Z1(zmeans=c(z1mean, z2mean, z3mean, z4mean), zmatrix1=zmatrix2, b1=b2, prevVector=prevVector1,
            ProbSel1=ProbSel))/ProbSel
        ExpSel2_Z3 <- (ExpectedSel2_Z3(zmeans=c(z1mean, z2mean, z3mean, z4mean), zmatrix1=zmatrix2, b1=b2, prevVector=prevVector1,
            ProbSel1=ProbSel))/ProbSel
        ExpSel2_Z4 <- (ExpectedSel2_Z4(zmeans=c(z1mean, z2mean, z3mean, z4mean), zmatrix1=zmatrix2, b1=b2, prevVector=prevVector1,
            ProbSel1=ProbSel))/ProbSel

        Sel2_Z1 <- ExpSel2_Z1
        Sel2_Z2 <- (ExpSel2_Z2 - (prop[1] * ExpSel2_Z1))/(prop[2])
        Sel2_Z3 <- (ExpSel2_Z3 - ExpSel2_Z2)/(prop[3])
        Sel2_Z4 <- (ExpSel2_Z4 - ExpSel2_Z3)/(prop[4])

        zr[1] <- y[1] - (r*(Sel2_Z1-y[1]))
        zr[2] <- y[2] - (r*(Sel2_Z2-y[2]))
        zr[3] <- y[3] - (Sel2_Z3-y[3])
        zr[4] <- y[4] - (Sel2_Z4-y[4])

        euc.dis <- sqrt(sum((zrminus - zr)*(zrminus - zr)))
        if (euc.dis <= 0.001) zrminus = zr; break()
        else {zrminus = zr}
    }
    return(c(zrminus,k))
}
```

7.2.8 Multiple iterations bias adjusted estimate

```
prop <- c(0.25, 0.25, 0.25, 0.25)
prop <- rev(prop)
test <- twosubs.selected(zmatrix2=zmatrix, NaiveEstimates=mu, b2=b, prob2=prop, prevVector1=c(p1, p2, p3, p4), r=t)
MultipleIterationsBiasAdjustedEstimate <- round((((prop[4] * test[1]) + (prop[3] * test[2]))/(prop[3] + prop[4]), 3)
MultipleIterationsBiasAdjustedEstimate
```

7.2.9 First shrinkage estimate

Proportion of stage 1 data for S_2

```
t <- round(180/(180+240), 3)
```

Calculating $\hat{C}_+$ defined in Section 3.4 of the main paper

```
c.hat <- 45 * ((3 - 1.45)*(3 - 1.45)) * ((2-1.45)*(2-1.45)) * ((0.8-1.45)*(0.8-1.45)) * ((0-1.45)*(0-1.45))
c.hat <- (2*7*7)/c.hat
c.hat <- 1 - c.hat
c.hat <- round(c.hat, 3)
c.hat <- max(0, c.hat)
c.hat
```

Shrinkage estimate for partition 1

```
lind.estimates1 <- (t * ((c.hat*3)+((1-c.hat)*1.45))) + ((1-t)*3.0)
lind.estimates1 <- round(lind.estimates1, 3)
lind.estimates1
```

Shrinkage estimate for partition 2

```
lind.estimates2 <- (t * ((c.hat*2)+((1-c.hat)*1.45))) + ((1-t)*2.4)
lind.estimates2 <- round(lind.estimates2, 3)
```

```
lind.estimates2
```

Shrinkage estimate for effect in S_2

```
LindEstimate1 <- round(((0.25 * lind.estimates1) + (0.25 * lind.estimates2))/0.5, 3)
LindEstimate1
```

7.2.10 Second shrinkage estimate

Function for iteratively estimating $\tilde{C}_+$ defined in Section 3.4 of the main paper

```
EstimatingTau <- function(EigenValues, NaiveEstimates){
    y <- c(NaiveEstimates[1], NaiveEstimates[2], NaiveEstimates[3], NaiveEstimates[4])

    d1 <- (EigenValues[1])*(EigenValues[1])
    d2 <- (EigenValues[2])*(EigenValues[2])
    d3 <- (EigenValues[3])*(EigenValues[3])
    d4 <- (EigenValues[4])*(EigenValues[4])

    e1 <- ((y[1] - y[4])*(y[1] - y[4])) - d1
    e2 <- ((y[2] - y[4])*(y[2] - y[4])) - d2
    e3 <- ((y[3] - y[4])*(y[3] - y[4])) - d3
    e4 <- ((y[4] - y[4])*(y[4] - y[4])) - d4

    zrminus <- 0.05

    for (k in 1:200){
        zr <- NA

        w1 <- 1/(zrminus + d1)
        w2 <- 1/(zrminus + d2)
        w3 <- 1/(zrminus + d3)
        w4 <- 1/(zrminus + d4)

        w <- w1 + w2 + w3 + w4

        ww1 <- (w1 * e1) + (w2 * e2) + (w3 * e3) + (w4 * e4)

        zr <- ww1/w

        euc.dis <- sqrt((zrminus - zr)*(zrminus - zr))
        if (euc.dis <= 0.0001) {zrminus <- zr; break()}
            else {zrminus <- zr}
    }
    zrminus <- max(0,zrminus)
    return(c(zrminus,k))
}
```

Calculating the multiple iterations bas adjusted estimate

```
ymatrix <- matrix(NA, ncol=4, nrow=4)
ymatrix[1,]
ymatrix[1,] <- c(2.178, 1.089, 0.726, 0.545)
ymatrix[2,] <- c(1.089, 1.089, 0.726, 0.545)
ymatrix[3,] <- c(0.726, 0.726, 0.726, 0.545)
ymatrix[4,] <- c(0.545, 0.545, 0.545, 0.545)
ymatrix

test <- svd(ymatrix)
EigenValues1 <- test$d
round(EigenValues1, 3)
EigenValues1

nu.squared <- EstimatingTau(EigenValues1, c(3, 2.5, 1.93, 1.45))[1]
new.matrix <- nu.squared * diag(4) + ymatrix
new.matrix <- solve(new.matrix)
new.matrix <- diag(4) - ymatrix %*% new.matrix
new.matrix <- round(new.matrix, 3)

LindEstimate2 <- new.matrix %*% matrix(c(3, 2.5, 1.93, 1.45), ncol=1) + ((diag(4) - new.matrix) %*% matrix(rep(1.45,4), ncol=1))
LindEstimate2 <- as.vector(LindEstimate2)
```

```
LindEstimate2 <- round(LindEstimate2, 3)

OverallLindsayEstimate <- (t * LindEstimate2[2]) + ((1-t)*2.7)
OverallLindsayEstimate <- round(OverallLindsayEstimate, 3)
OverallLindsayEstimate
```

8 All simulation results

8.1 Justifying sample size of 800 in the simulations

The sample size of 800 was informed by ensuring sufficient power for main effect in S_2 when S_2 is selected to be tested in stage 2. For a single-population and a one-stage trial comparing two arms, 468 patients are required to detect a difference of 0.3 with 90% power for main effect of the treatment. Thus, with 800 patients and stage 1 consisting of 600 patients (150 patients in each partition), when S_2 is selected, the total number of patients from the subpopulation is 500, giving the desired power. When S_1 is selected, the power is 80% while the power would be greater than 90% if S_3 or F are selected because more patients from stage 1 are used in the comparisons. We will consider two other cases where stage 1 consists of 200 and 400 patients so that stage 2 consists of 600 and 400 patients, respectively. For these cases, power is at least 90% for all the possible selections. Note that these power estimates do not take into account the observed effect sizes in stage 1 and so are approximate.

8.2 Explaining the negative and positive biases

To describe the negative bias of the naive estimator when S_3 (Column 2 in Figure 1 below) in Scenarios 1 and 2, note that for S_3 to be selected, $\bar{x}_{14} < b$ and $b \leq \bar{y}_{13} < (4b - \bar{x}_{14})/3$. Thus the value of θ_3 and the distribution of $\bar{x}_{14}$ values when S_3 is selected explain the mean bias. For example, as $\bar{x}_{14} \longrightarrow b$, $(4b - \bar{x}_{14})/3 \longrightarrow b$ so that for the cases where S_3 is selected and $\bar{x}_{14}$ is close to b, $\bar{y}_{13}$ will be close to b. Consequently, for a scenario where θ_3 is substantially greater than b and $\bar{x}_{14}$ tends to be close to b whenever S_3 is selected, the naive estimator for θ_3 is negatively biased because it is a function of $\bar{Y}_{13}$. This is approximately the case for Scenarios 1 and 2. Using Scenario 1 (Top row) for explicit explanation, we examined $\bar{x}_{14}$ values when S_3 was selected. In 5021 simulations out of the 5029 simulations where S_3 was selected, $\bar{x}_{14}$ values were ≥ -0.9. Noting that $b = 0 \leq \bar{y}_{13} < 0.3 = \theta_3$ for $\bar{x}_{14}$ values above -0.9, it means that in almost all simulations, stage 1 estimates ($\bar{y}_{13}$) underestimate θ_3, explaining the negative bias in Scenario 1.

Explanation of positive mean bias is straightforward when S_1 is selected in Scenario 3 (Column 4, Top plot in Figure 1 below). Note that S_1 is selected when $\bar{x}_{12} + \bar{x}_{13} + \bar{x}_{14} < 3b$ and $b \leq \bar{x}_{11} < \{4b - (\bar{x}_{12} + \bar{x}_{13} + \bar{x}_{14})\}$. Thus θ_1 will be overestimated since S_1 is selected when $\bar{x}_{11} \geq 0$ which leads to positive bias since $\theta_1 = 0$.

8.3 Biases including case of $n_1 = 400$

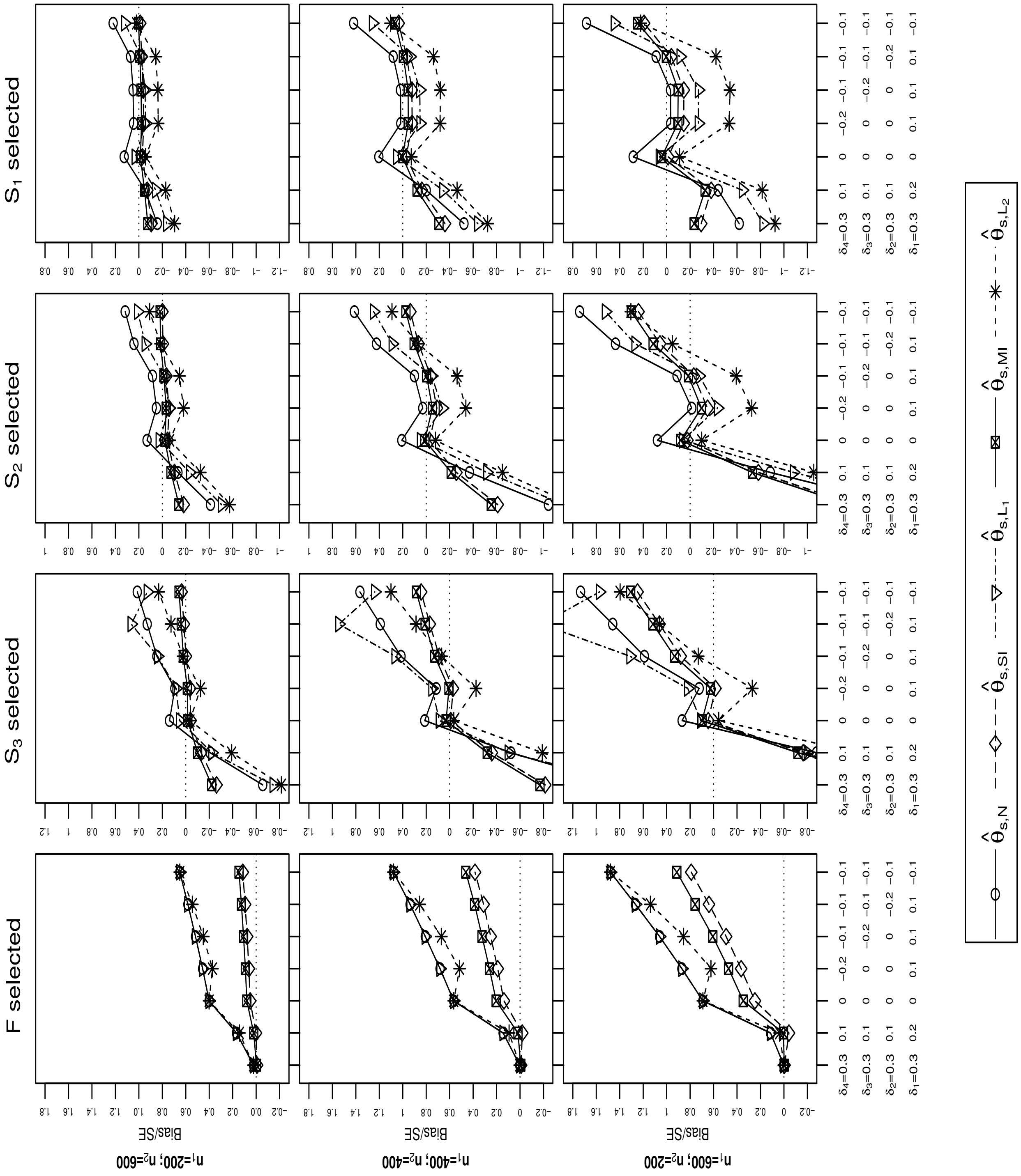

Figure 1: This is an extension of Figure 2 in the main paper to include the case of $n_1 = 400$. Biases in units of approximate standard error for different configurations. The dotted line is the point of no bias. Other line types correspond to different estimators.

8.4 Mean squared error including case of $n_1 = 400$

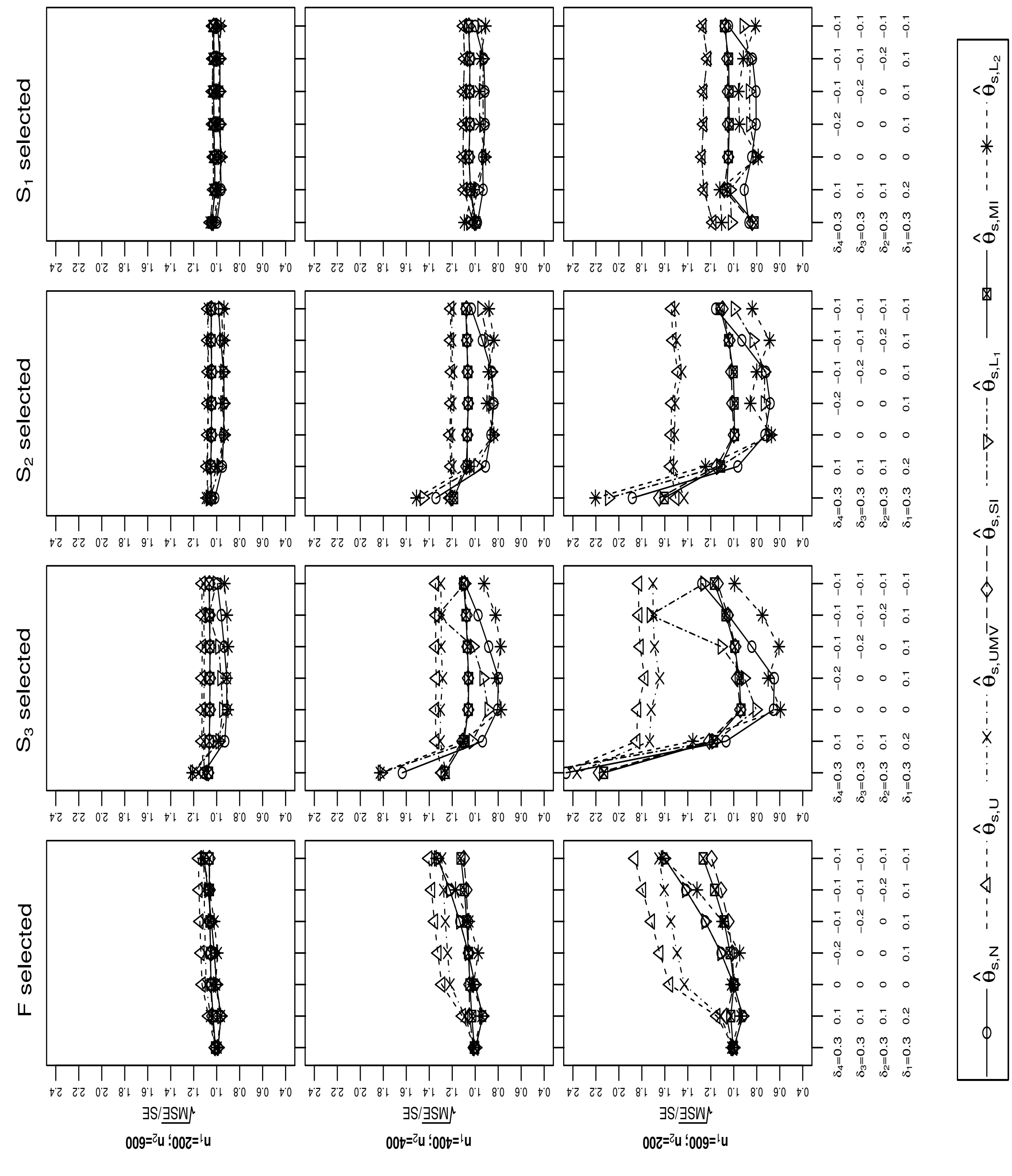

Figure 2: This is an extension of Figure 3 in the main paper to include the case of $n_1 = 400$. Root mean squares in units of approximate standard error for different configurations. Different line types correspond to different estimators.

8.5 Boxplots of estimates when $n_1 = 200$ and F or S_3 are selected

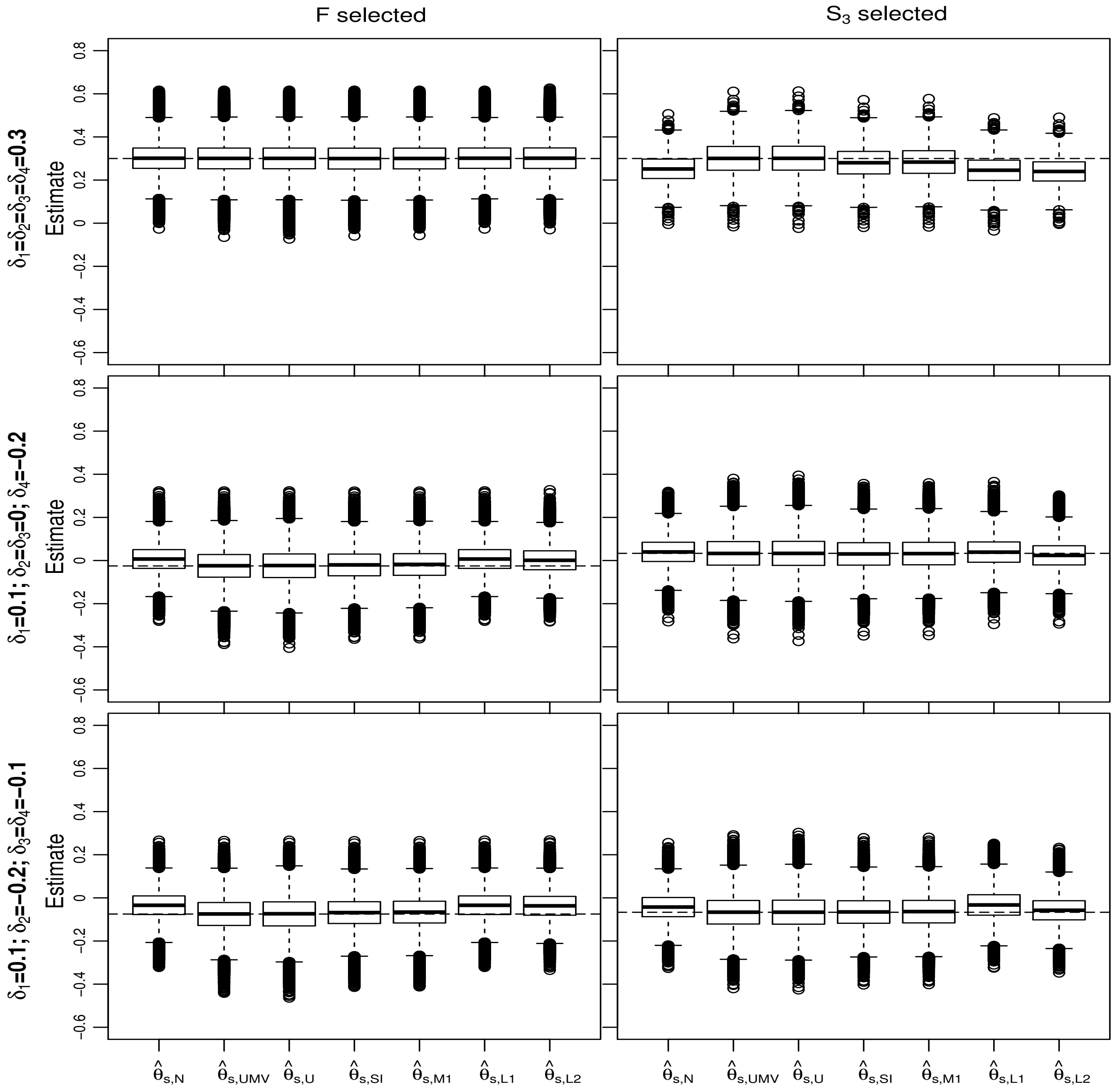

Figure 3: This is a copy of Figure 4 in the main paper. Boxplots of estimates for different estimators when $n_1 = 200$. Results have been chosen when F and S_3 were selected and for Scenarios 1 (Top panels), 4 (Middle panels) and 6 (Bottom panels). The dashed lines correspond to the true means in the selected subpopulation.

8.6 Boxplots of estimates when $n_1 = 200$ and S_2 or S_1 are selected

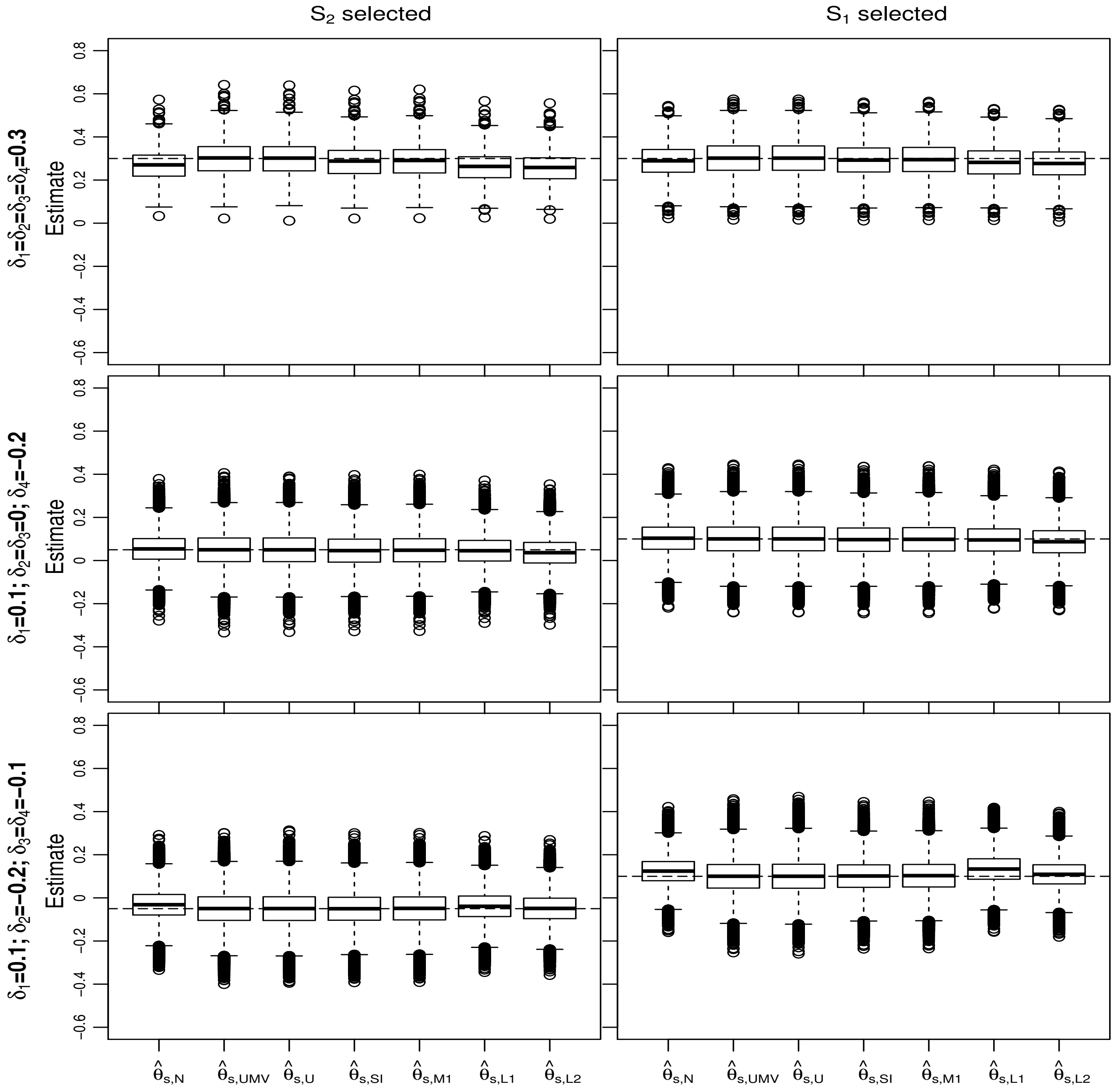

Figure 4: Boxplots of estimates for different estimators when $n_1 = 200$. Results have been chosen when S_2 and S_1 were selected and for Scenarios 1 (Top panels), 4 (Middle panels) and 6 (Bottom panels). The dashed lines correspond to the true means in the selected subpopulation.

8.7 Boxplots of estimates when $n_1 = 600$ and F or S_3 are selected

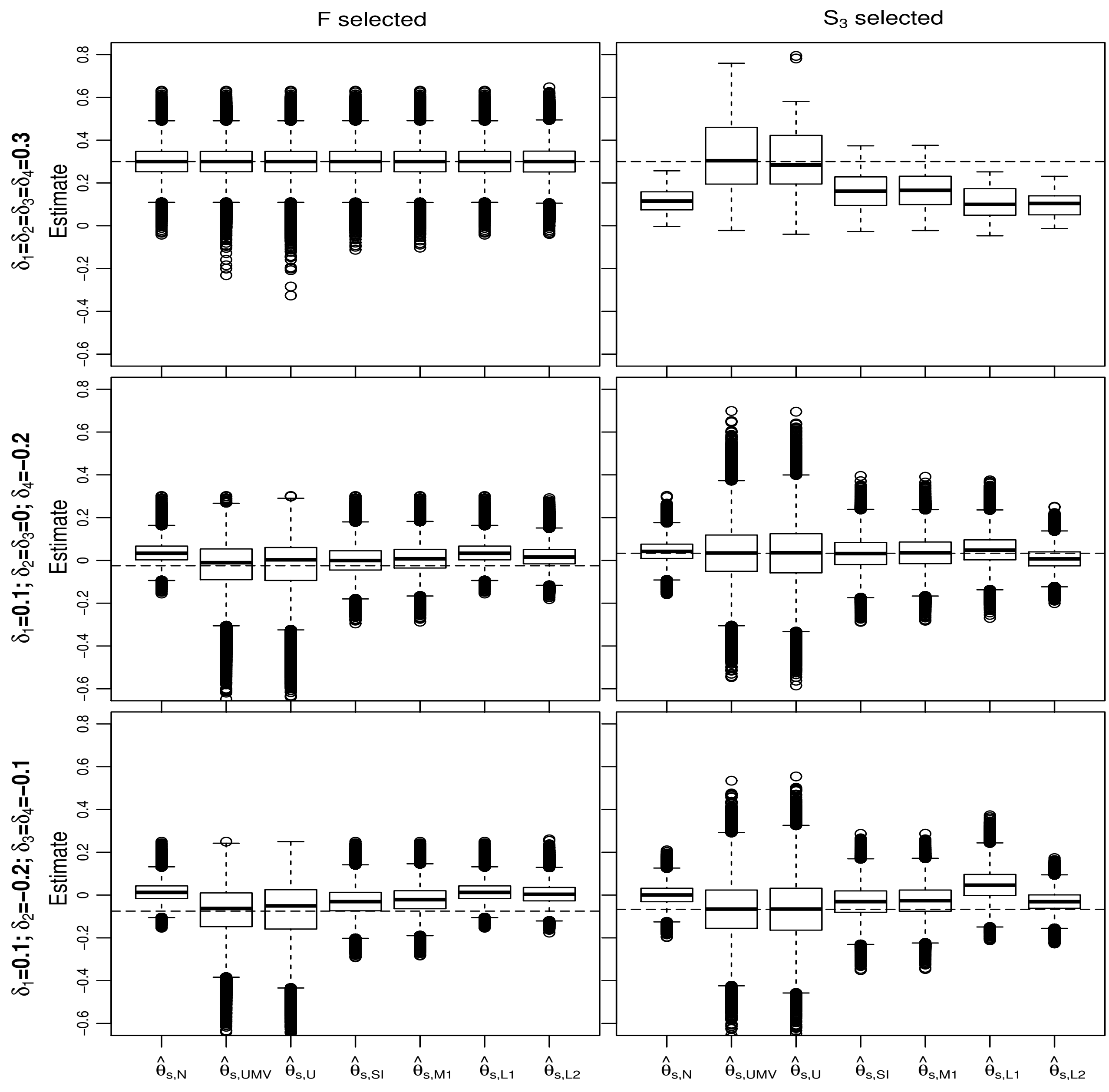

Figure 5: This is a copy of Figure 5 in the main paper. Boxplots of estimates for different estimators when $n_1 = 600$. Results have been chosen when F and S_3 were selected and for Scenario 1 (Top panels), 4 (Middle panels) and 6 (Bottom panels). The dashed lines correspond to the true means in the selected subpopulation.

8.8 Boxplots of estimates when $n_1 = 600$ and S_2 or S_1 are selected

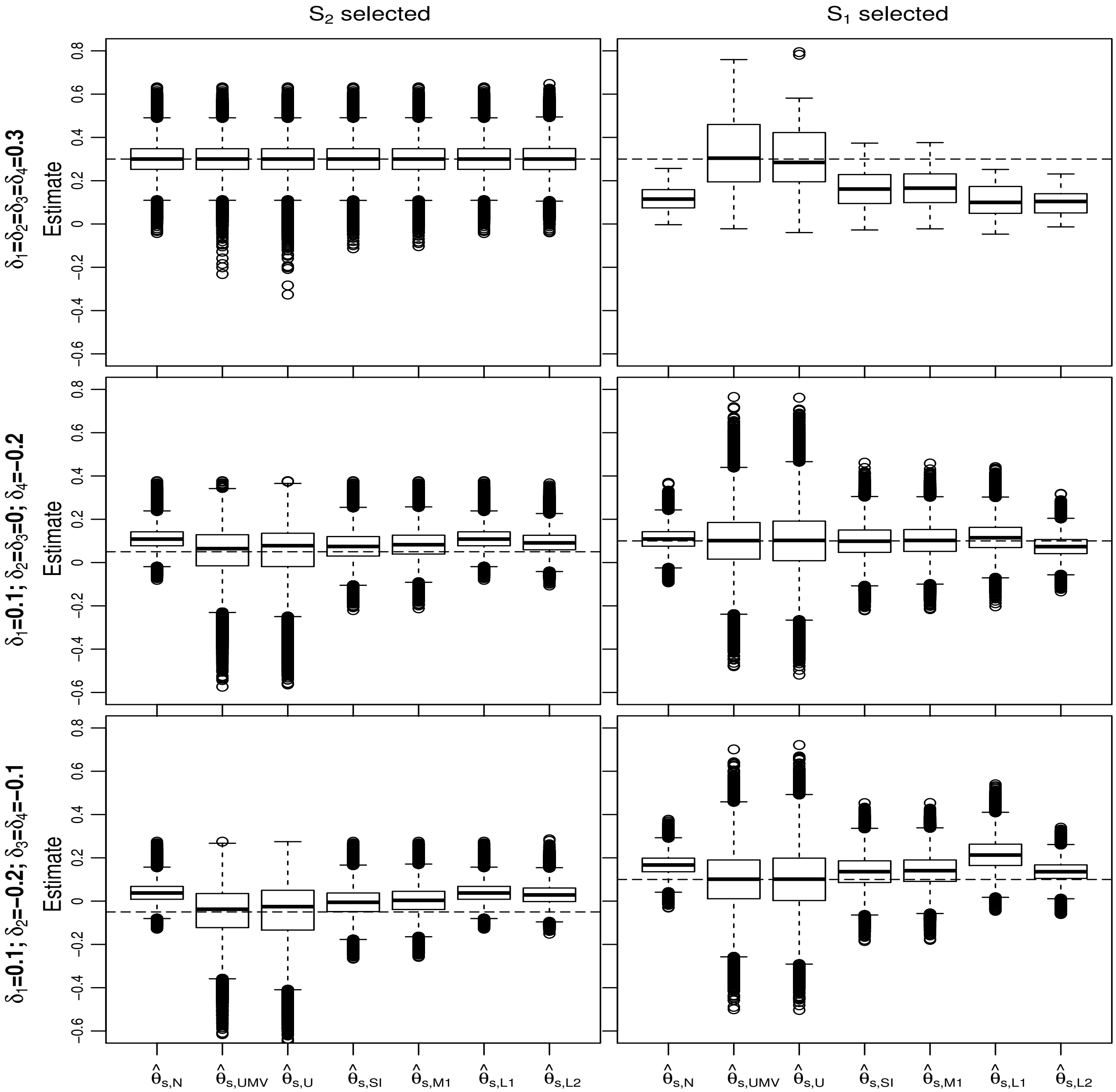

Figure 6: Boxplots of estimates for different estimators when $n_1 = 600$. Results have been chosen when S_2 and S_1 were selected and for Scenario 1 (Top panels), 4 (Middle panels) and 6 (Bottom panels). The dashed lines correspond to the true means in the selected subpopulation.

8.9 Boxplots of estimates when $n_1 = 400$ and F or S_3 are selected

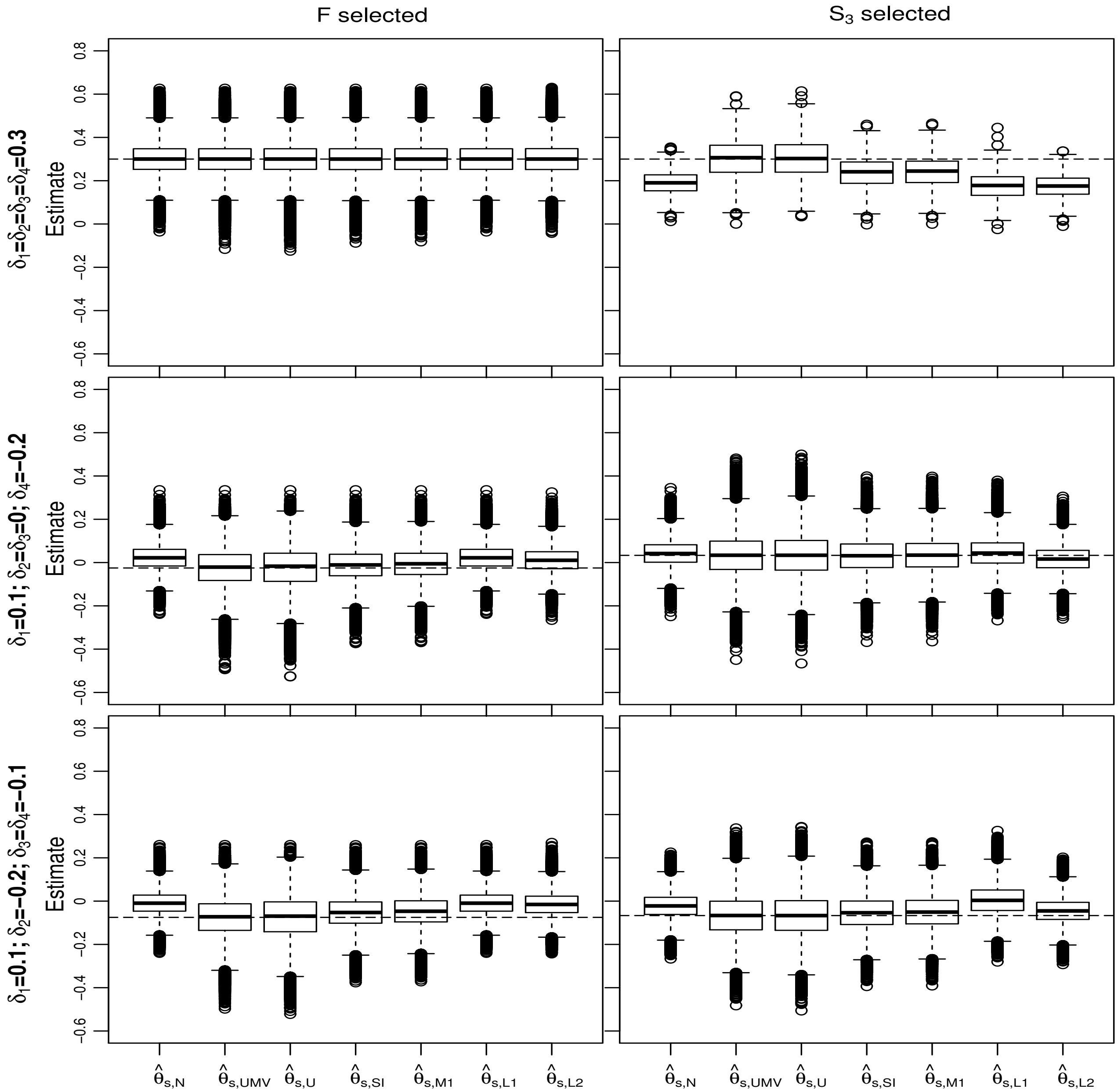

Figure 7: Boxplots of estimates for different estimators when $n_1 = 400$. Results have been chosen when F and S_3 were selected and for Scenario 1 (Top panels), 4 (Middle panels) and 6 (Bottom panels). The dashed lines correspond to the true means in the selected subpopulation.

8.10 Boxplots of estimates when $n_1 = 400$ and S_2 or S_1 are selected

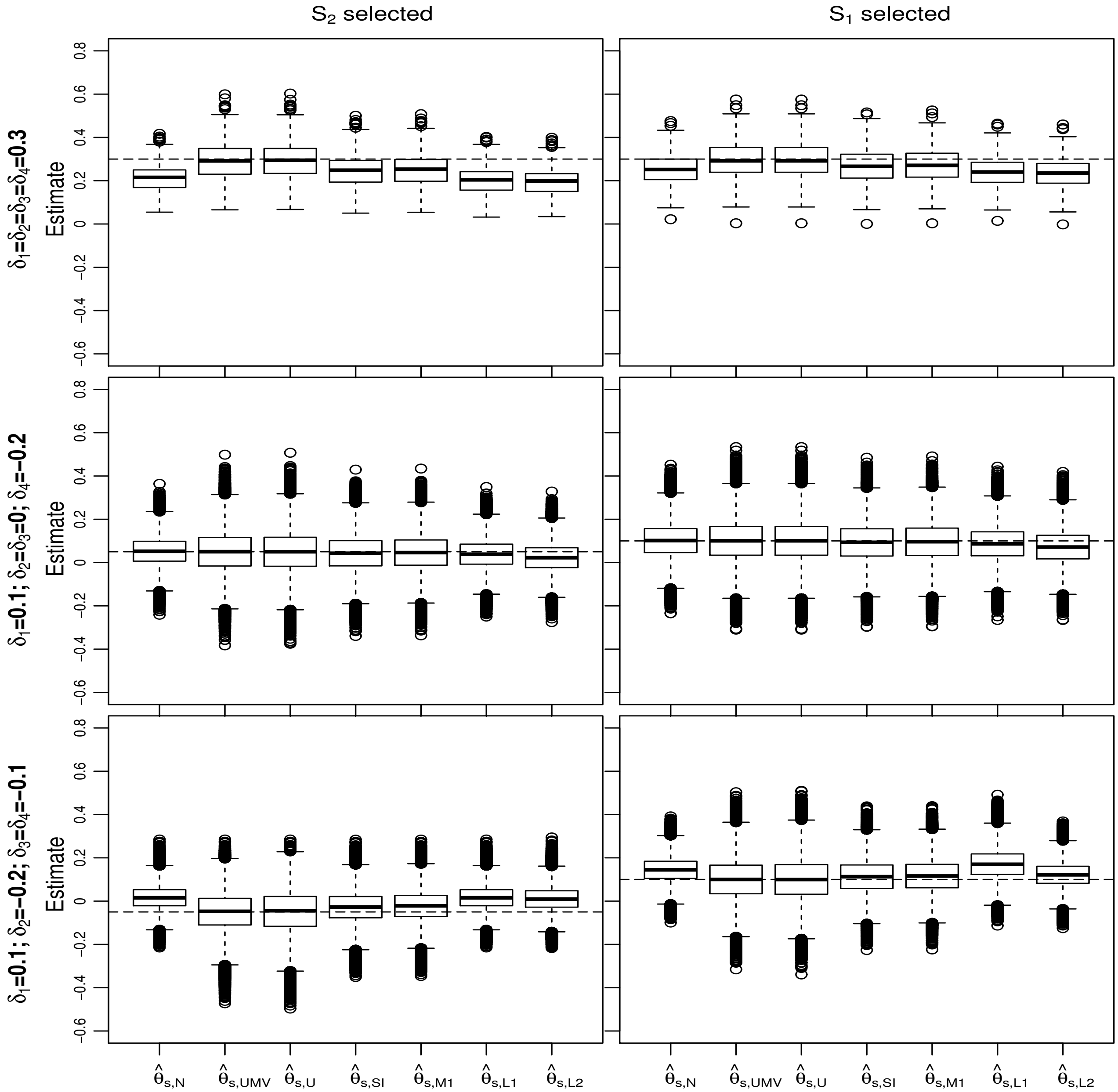

Figure 8: Boxplots of estimates for different estimators when $n_1 = 400$. Results have been chosen when S_2 and S_1 were selected and for Scenario 1 (Top panels), 4 (Middle panels) and 6 (Bottom panels). The dashed lines correspond to the true means in the selected subpopulation.

9 Simulations when the number of partitions is eight

To assess the effect of the number of partitions, we performed simulations when $n_1 = 400$ and there are 8 candidate partitions, that is, $K = 8$. So that the results are approximately comparable to previous settings, we have considered scenarios that are such that quartiles have the same treatment effect sizes as the scenarios in Table 3 in the main paper. When $K = 8$, F, S_6, S_4 and S_2 have equal prevalences as F, S_3, S_2 and S_1 when $K = 4$. Hence when $K = 8$, we only provide results when F, S_6, S_4 and S_2 are selected to compare with results in Sections 5.2.1 to 5.2.3 in the main paper. Considering these subpopulations only saved considerable computation time.

Table 2 summarises the true treatment effects and the probabilities of selecting subpopulations F, S_6, S_4 and S_2. These are compared to results in Table 4 in the main paper when $n_1 = 400$. As expected, the probabilities of selecting F are the same in both tables. Comparing S_3, S_2 and S_1 in Table 4 in the main paper to S_6, S_4 and S_2 in Table 2, respectively, the probabilities in Table 2 are approximately half of those in Table 4 in the main paper. This is expected as there are more subpopulations that can be selected to continue to stage 2 for the case of $K = 8$.

Figure 9 shows biases of various estimators for the case of $K = 8$ when F, S_6, S_4 and S_2 are selected. Due to increased computation time, we simulated $N = 100,000$ trials. We compare the

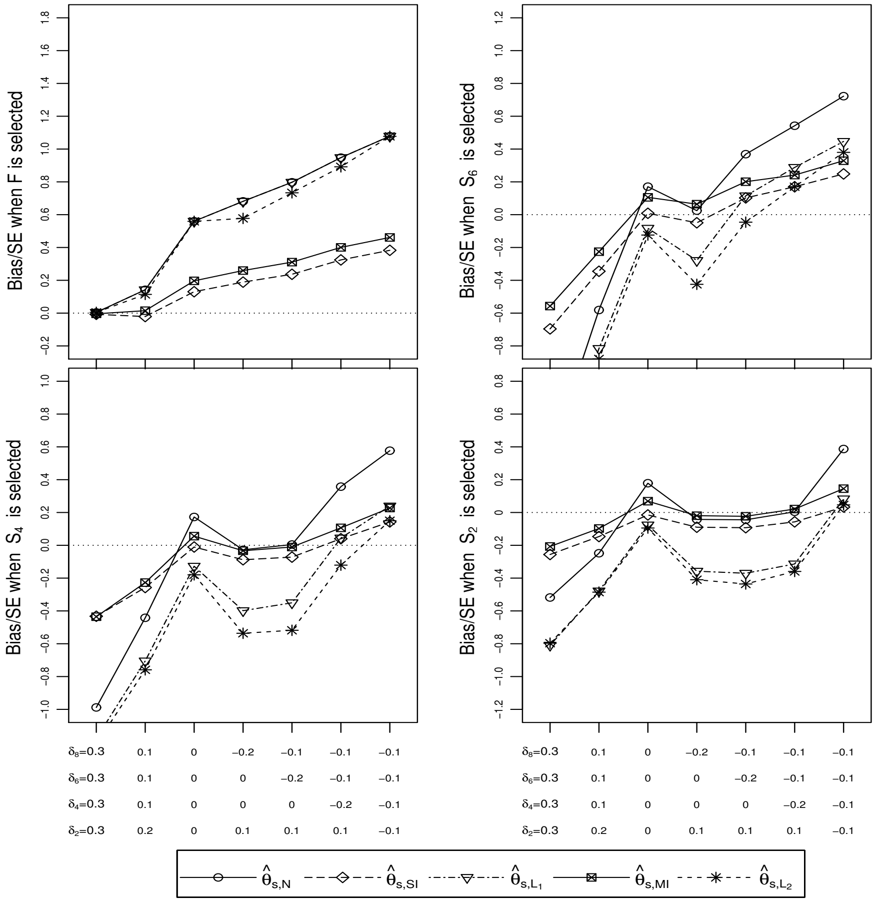

Figure 9: Biases in units of approximate standard error when full population has eight candidate partitions. In all plots $\delta_1 = \delta_2$, $\delta_3 = \delta_4$, $\delta_5 = \delta_6$ and $\delta_7 = \delta_8$. Different line types correspond to different estimators.

Table 2: Treatment effect for different scenarios for a case with eight partitions when

Scenario	Treatment effect								Ideal selection	Probability of a decision ($n_1 = 400$)			
	δ_1	δ_2	δ_3	δ_4	δ_5	δ_6	δ_7	δ_8		F	S_6	S_4	S_2
1	0.3	0.3	0.3	0.3	0.3	0.3	0.3	0.3	F	0.9987	0.0002	0.0001	0.0001
2	0.2	0.2	0.1	0.1	0.1	0.1	0.1	0.1	F	0.8944	0.0146	0.0101	0.0097
3	0	0	0	0	0	0	0	0	F	0.5000	0.0438	0.0371	0.0405
4	0.1	0.1	0	0	0	0	-0.2	-0.2	S_7	0.4013	0.1209	0.0494	0.0501
5	0.1	0.1	0	0	-0.2	-0.2	-0.2	-0.2	S_5	0.3085	0.0673	0.1303	0.0647
6	0.1	0.1	-0.2	-0.2	-0.1	-0.1	-0.1	-0.1	S_1	0.2266	0.0554	0.0656	0.1677
7	-0.1	-0.1	-0.1	-0.1	-0.1	-0.1	-0.1	-0.1	Stop	0.1587	0.0421	0.0493	0.0700

biases in this figure to those in the middle row in Figure 1. As expected, when F is selected, biases for the naive estimator are equal. Comparing results for S_3, S_2 and S_1 in Figure 1 to results for S_6, S_4 and S_2 in Figure 9, respectively, biases of the naive estimator are slightly larger in Figure 9. When F is selected, biases for $\hat{\theta}_{s,SI}$ in Figures 1 and 9 are equal. Similar results are observed for $\hat{\theta}_{s,MI}$. Comparing results for S_3, S_2 and S_1 in Figure 1 to results for S_6, S_4 and S_2 in Figure 9, respectively, magnitude of the biases for $\hat{\theta}_{s,SI}$ are approximately equal in both figures. On the other hand, $\hat{\theta}_{s,MI}$ seems to perform better when there are more candidate partitions.

Figure 10 shows MSEs of various estimators for the case of $K = 8$ when F, S_6, S_4 and S_2 are selected. We compare the MSEs in this figure to those in the middle row in Figure 2. Making comparisons such as those made for biases, we note that MSEs for estimators $\hat{\theta}_{s,N}$, $\hat{\theta}_{s,SI}$, $\hat{\theta}_{s,MI}$ and $\hat{\theta}_{s,UMV}$ in Figures 1 and 9 are approximately equal. However, the MSE for $\hat{\theta}_{s,U}$ is higher when $K = 8$ than when $K = 4$.

We note that based on the comparison of results when $K = 4$ and when $K = 8$, the recommendation for how the choice of estimator is made when $K = 8$ is similar to when $K = 4$. Since in practice we would not expect very many candidate partitions, the recommendation generalises to most plausible settings.

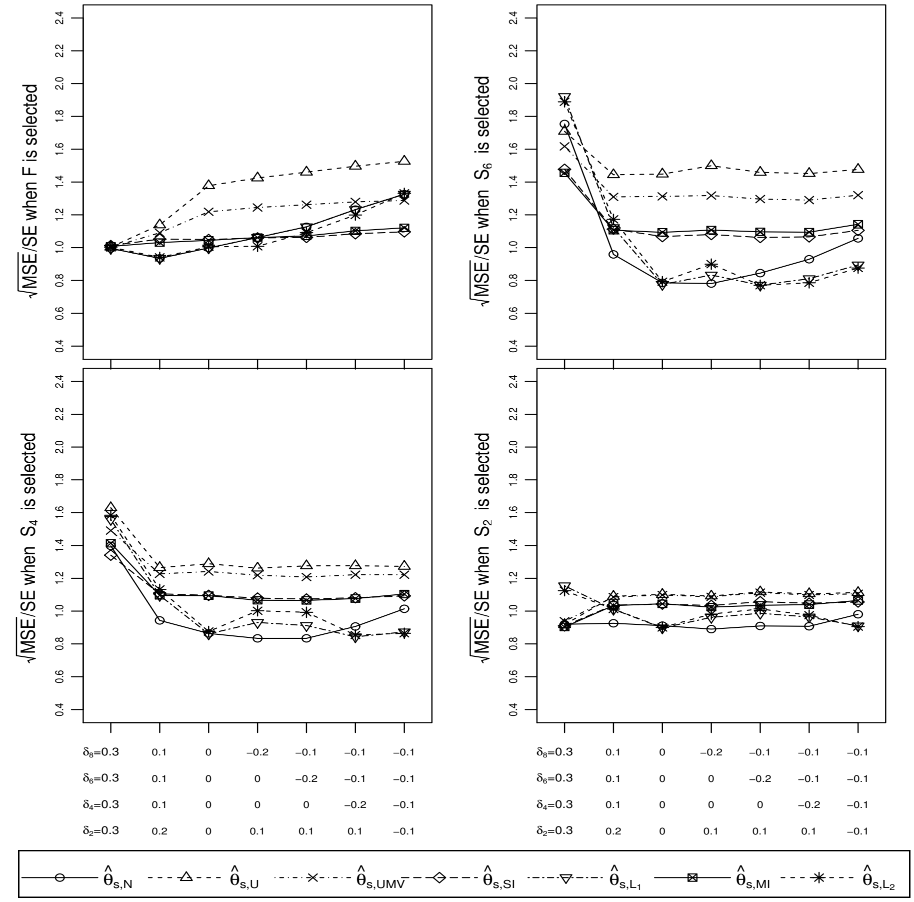

Figure 10: Root mean squares in units of approximate standard error when full population has eight candidate partitions. In all plots $\delta_1 = \delta_2$, $\delta_3 = \delta_4$, $\delta_5 = \delta_6$ and $\delta_7 = \delta_8$. Different line types correspond to different estimators.

10 Simulation result when "shrinking" is to $(\bar{y}_{11}+\bar{y}_{12}+\bar{y}_{13}+\bar{y}_{14})/4$

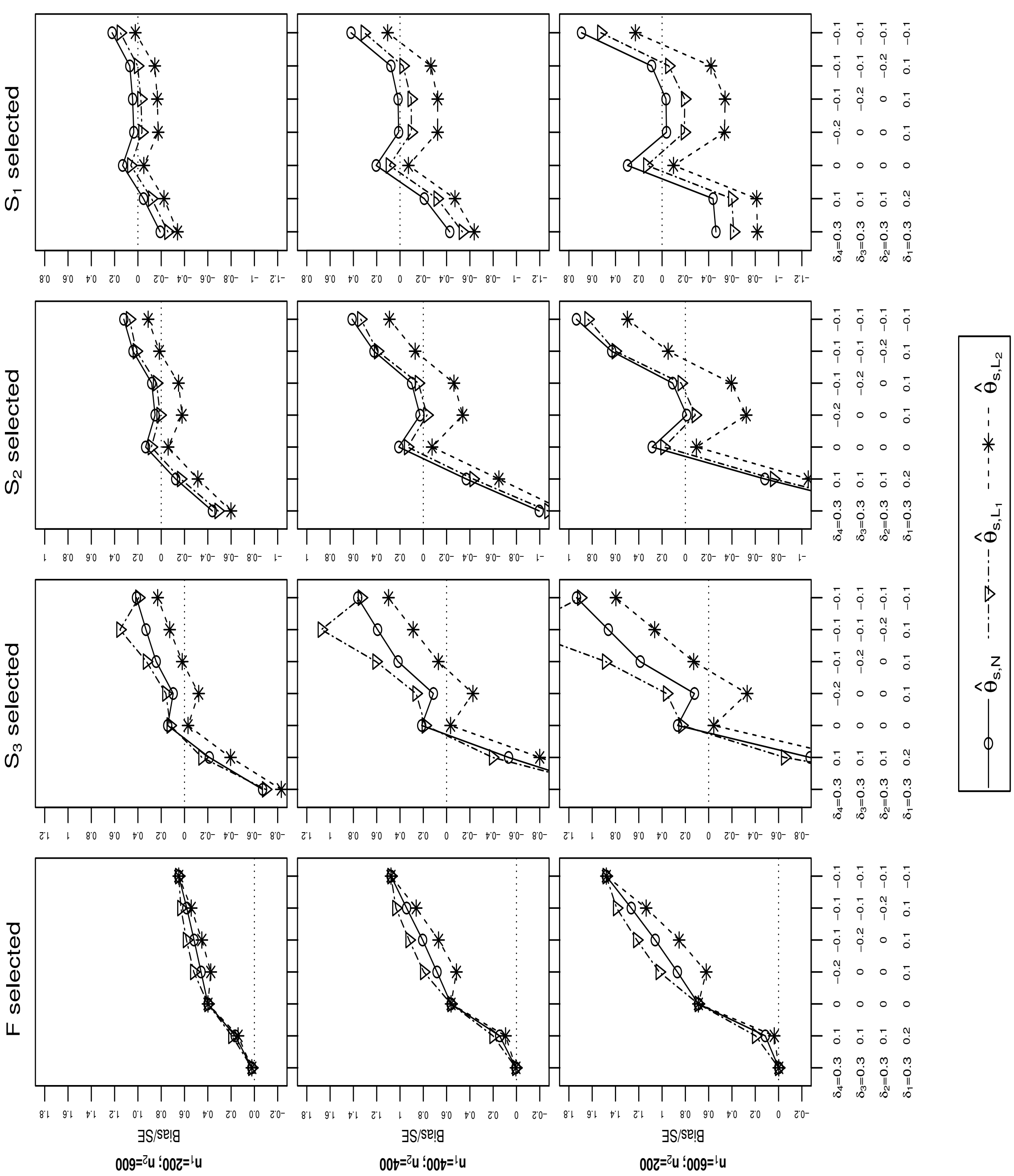

Figure 11: Biases in units of approximate standard error for different configurations. Continuous, short & long dashed and dashed lines correspond to the naive, first shrinkage and second shrinkage estimators. The shrinkage estimators in this figure and Figure 1 are compared to assess effect of shrinking to different quantities.

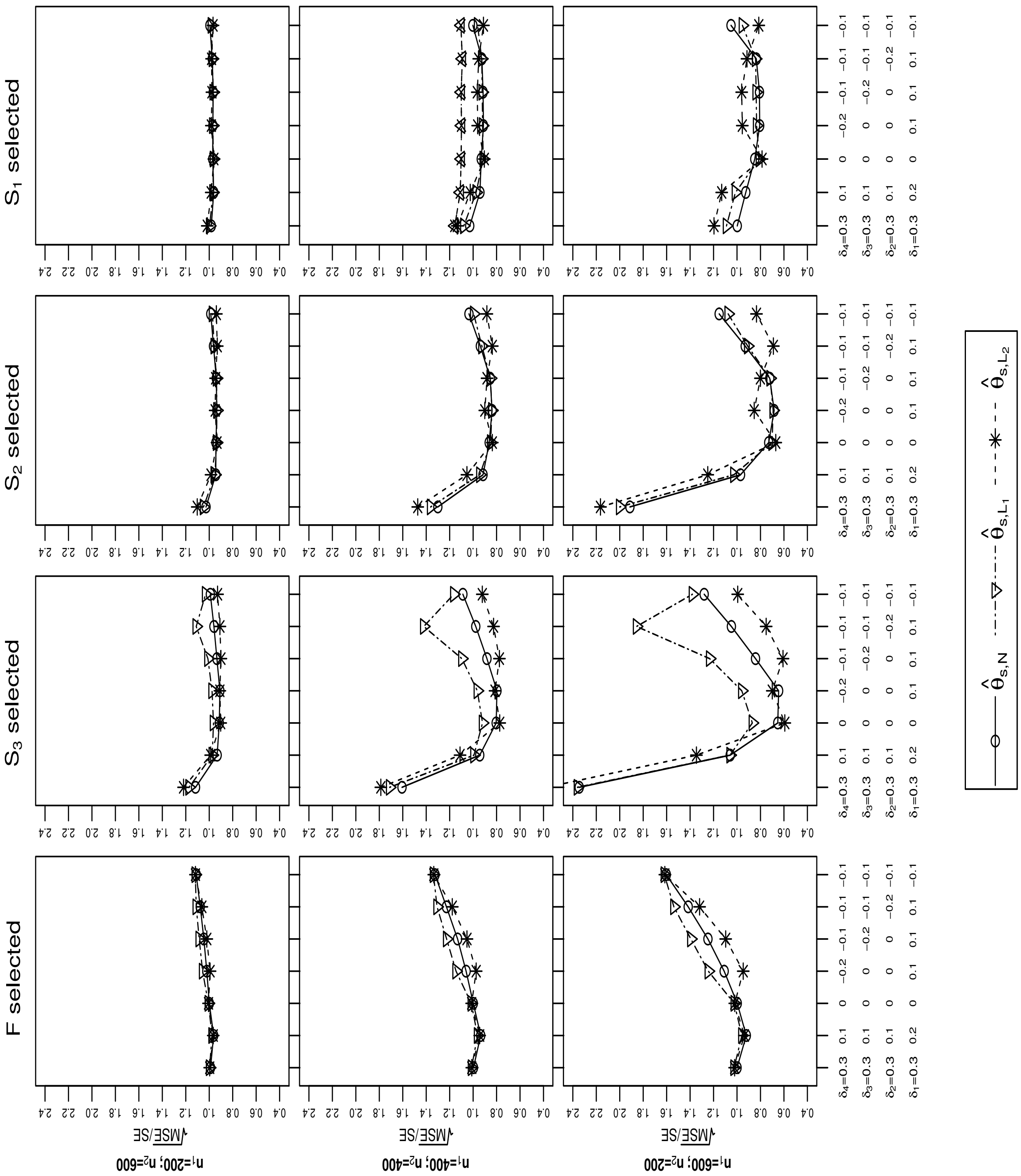

Figure 12: Root mean squares in units of approximate standard error for different configurations. Continuous, short & long dashed and dashed lines correspond to the naive, first shrinkage and second shrinkage estimators. The shrinkage estimators in this figure and Figure 2 are compared to assess effect of shrinking to different quantities.
